# A selective RPL15 PROTAC degrader enhances anti-PD-1 immunotherapy in a murine melanoma tumor model

**DOI:** 10.1038/s41388-025-03641-4

**Published:** 2025-11-23

**Authors:** Runa Takahashi, Kazuki Yamamoto, Hikaru Toya, Haruka Shoji, Kohei Kawanishi, Kyoka Momosaki, Miyuki Yabe, Ken Takashima, Ryuta Muromoto, Satoshi Ichikawa, Tadashi Matsuda, Yuichi Kitai

**Affiliations:** 1 Department of Immunology, Graduate School of Pharmaceutical Sciences, Hokkaido University, Hokkaido, Japan; 2 Faculty of Pharmaceutical Sciences, Hokkaido University, Sapporo, Japan; 3 RNA Biology Laboratory, Faculty of Pharmaceutical Sciences, Hokkaido University, Sapporo, Japan; 4 Department of Immunology, Faculty of Life Sciences, Graduate School of Medical Sciences, Kumamoto University, Kumamoto, Japan; 5 Laboratory of Microbiology and Immunology, Graduate School of Pharmaceutical Sciences, Health Sciences University of Hokkaido, Hokkaido, Japan; 6 Center for Research and Education on Drug Discovery, Faculty of Pharmaceutical Sciences, Hokkaido University, Sapporo, Japan; 7 Global Station for Biosurfaces and Drug Discovery, Global Institution for Collaborative Research and Education (GI-CoRE), Hokkaido University, Sapporo, Japan; 8 Institute of Medical, Pharmaceutical and Health Sciences, Kanazawa University, Kakuma-machi, Kanazawa, Japan

**Keywords:** Tumour immunology, Innate immunity

## Abstract

Damage-associated molecular patterns (DAMPs) are secreted from damaged or dying cells and activate innate immune signaling via pattern-recognition receptors such as Toll-like receptors and cGAS. We previously showed that topotecan, a chemotherapeutic drug and topoisomerase I (TOP1) inhibitor, binds to ribosomal protein RPL15 and induces the secretion of DAMPs from cancer cells, which activate cGAS-STING signaling in dendritic cells. RPL15-knockdown B16-F10 melanoma tumors were sensitized to anti-PD-1 antibody, suggesting that RPL15 inhibition may have the potential to improve immune checkpoint inhibitor efficacy. However, topotecan and its derivatives, including SN-38, are highly cytotoxic because of their TOP1 inhibitory activity. Here, we synthesized SN-38-conjugated pomalidomide (SN38-PROTAC) and showed that SN38-PROTAC induced ubiquitin-mediated degradation of RPL15, but not TOP1. SN38-PROTAC treatment induced DAMP secretion from cancer cells, which activated cGAS-STING signaling in dendritic cells. The cytotoxicity of SN38-PROTAC in MCF7 cells was 100-fold lower than SN-38. SN38-PROTAC treatment increased the CTL/Treg ratio in tumors and sensitized B16-F10 tumors to anti-PD-1 antibody in a mouse model. The enhanced antitumor effects of SN38-PROTAC and anti-PD-1 antibody combination were abolished in STING-deficient mice. Our results indicate that SN38-PROTAC, which induces RPL15 degradation, has the potential to enhance ICI efficacy in PD-1-resistant cancer with low cytotoxicity.

## Introduction

Immune checkpoint proteins, such as PD-1 and CTLA-4, repress aberrant activation of immune cells and autoimmunity. Cancer cells use these proteins to escape from attack by cytotoxic T lymphocytes (CTLs) [1–3]. Immune checkpoint inhibitors (ICI) block the binding between checkpoint proteins and their partners, resulting in the activation of CTL-mediated antitumor immune responses. ICIs are used for treatment against melanoma, breast cancer, and lung cancer. However, the therapeutic efficacy varies among patients because CTL-mediated killing of cancer cells is affected by various factors, including oncogenes, metabolism, and immune cell composition of the tumor, which are highly individual [4–7]. Tumor-infiltrating regulatory T cells (Treg) suppress CTL activation against cancer cells, and the Treg population inversely correlates with prognosis after ICI therapy [8, 9]. Thus, the tumor microenvironment strongly influences the efficacy of ICI against cancer.

ICI resistance is a common problem in ICI therapy against several cancers. In melanoma, the overall response rate was 44% in the patients treated with anti-PD-1 therapy; however, another report on patients with melanoma treated with anti-PD-1 therapy demonstrated that 39% of responders had progressed at 5 years’ follow-up [10, 11]. Moreover, 64% of patients who initially respond to ICI develop acquired resistance against ICI therapy in non-small cell lung cancer [12]. It is required for the development of methods that alter ICI-resistant cancer to ICI-sensitive cancer by regulating the tumor microenvironment.

Damage-associated molecular patterns (DAMPs) are secreted from damaged or dying cells and include proteins, DNA, or ATP [13–15]. DAMPs activate innate immune signaling via pattern-recognition receptors such as Toll-like receptors, cGAS, and the NLRP3 inflammasome. cGAS recognizes pathogen- and dead cell-derived DNA and then synthesizes cyclic GMP-AMP from GTP and ATP, which acts as a ligand for STING. Following its binding to cyclic GMP-AMP, STING activates the transcription factors NF-κB and IRF3 to induce the expression of inflammatory cytokines and type I IFNs. DAMPs thereby act as an alarm to the immune system during infectious disease and tissue injury [16]. Some anticancer drugs reshape the tumor microenvironment and elicit antitumor immune responses via DAMP secretion. For example, doxorubicin and cisplatin induce HMGB1 and ATP secretion as DAMPs from cancer cells, resulting in alteration of immune cell composition and immune cell activation in the tumor, including CTL, Treg, and myeloid-derived suppressor cells [17–19].

Camptothecin and its derivatives, such as topotecan, irinotecan, and SN-38, inhibit topoisomerase I (TOP1) and induce cell death by triggering DNA double-strand breaks and DNA damage responses. Topotecan and irinotecan are used as chemotherapy against ovarian and small-cell lung cancer [20]. Our recent studies showed that topotecan, a TOP1 inhibitor, binds to ribosomal protein RPL15, resulting in the induction of ribosomal stress and the secretion of DNA-containing exosomes as DAMPs from cancer cells, which activate STING-dependent antitumor immune responses [21, 22]. Moreover, RPL15 knockdown in B16-F10 murine melanoma cells sensitized B16-F10 tumors to anti-PD-1 antibody treatment, suggesting that targeting RPL15 may improve ICI efficacy. However, the TOP1 inhibitor topotecan causes cytotoxicity against non-cancer cells. Therefore, camptothecin derivatives, including topotecan, irinotecan, and SN-38, that lack TOP1 inhibitory activity and act as an RPL15-specific inhibitor, have the potential to enhance ICIs via DAMP secretion.

Proteolysis targeting chimera (PROTAC) is are engineered molecules that target specific proteins for degradation. PROTAC comprises an E3 ubiquitin ligase ligand, linker, and the ligand of the target protein; it recruits the target protein to the E3 ubiquitin ligase, resulting in degradation of the target protein by the ubiquitin-proteasome pathway. Because PROTACs inhibit target proteins by inducing their degradation, they are effective inhibitors against undruggable proteins, such as scaffold proteins and transcription factors [23]. For instance, VZ185 degrades BRD7/9, which are co-activators of p53, and ARV-471, comprising an estrogen receptor ligand and thalidomide, promotes the degradation of estrogen receptor and inhibits the growth of estrogen receptor-positive breast cancer cells [24, 25]. Thalidomide and its analogues pomalidomide and lenalidomide bind to the E3 ubiquitin ligase cereblon (CRBN), resulting in an alteration of its substrate recognition in ubiquitination [26, 27]. Our previous study showed that both topotecan and SN-38 bind to RPL15, suggesting that topotecan- or SN-38-conjugated thalidomide derivatives act as a RPL15 degrader PROTAC [22].

Here, we synthesized SN-38-conjugated pomalidomide (SN38-PROTAC) and examined its anti-tumor effects and mechanism. We showed that SN38-PROTAC selectively degraded RPL15, but not TOP1, dependent on human CRBN expression. SN38-PROTAC treatment induced DAMP secretion from cancer cells, which activated cGAS-STING signaling in bone marrow-derived dendritic cells (BMDCs). The cytotoxicity of SN38-PROTAC in MCF7 cells was 100-fold lower than SN-38. SN38-PROTAC treatment increased the CTL/Treg ratio in tumors and sensitized human CRBN-expressing B16-F10 tumor-bearing mice to anti-PD-1 treatment. The enhanced antitumor effects of the SN38-PROTAC and anti-PD-1 antibody combination were absent in STING-deficient mice, indicating that SN38-PROTAC altered the tumor microenvironment dependent on STING signaling. Our results indicate that SN38-PROTAC degrades RPL15 protein and shows the potential to enhance ICI therapeutic efficacy in PD-1-resistant cancer with low cytotoxicity.

## Materials and methods

### Animals

All animal experiments were approved by the Institutional Animal Care and Use Committee of Hokkaido University (Approval No. 18-0024). All animals were maintained under pathogen-free conditions and in compliance with national and institutional guidelines. All protocols were approved by the Hokkaido University animal ethics committee and performed in accordance with relevant guidelines and regulations and ARRIVE guidelines. C57BL/6 mice were purchased from Sankyo Labo Service Corporation, Inc. (Tokyo, Japan). STING-deficient mice were purchased from RINEN BRC [28]. Myd88-deficient mice were prepared by iGONAD methods, as described previously, using Myd88-targeted crRNA (5′-UCCCACGUUAAGCGCGACCA-3′, Integrated DNA Technologies, Coralville, IA, USA), Cas9 tracrRNA (Integrated DNA Technologies), and recombinant HiFi Cas9 (Integrated DNA Technologies) [29]. Myd88-deficient mice were backcrossed five times to C57BL/6 mice, and the frameshift mutation in the Myd88 coding region was verified by DNA sequencing (Supplementary Fig. 1).

### Reagents and cells

SN-38 was purchased from Toronto Research Chemicals (Toronto, Canada). Pomalidomide-(PEG)_n_-azide was purchased from Sigma (St. Louis, MO, USA). Bortezomib was purchased from Adipogen Life Sciences (San Diego, CA, USA). MG132 was purchased from Millipore (Burlington, MA, USA). SP600125 was purchased from ChemScene (Monmouth Junction, NJ, USA). All cell lines were cultured in Dulbecco’s modified Eagle’s medium (DMEM; Sigma) supplemented with 10% fetal bovine serum (FBS; BioSera, Nuaille, France) and 0.05 mM 2-mercaptoethanol (Nacalai Tesque, Kyoto, Japan) at 37 °C in a humidified 5% CO_2_/95% air atmosphere. The preparation of TOP1-deficient MCF7 cells was previously described [22].

### Synthesis of SN38-PROTACs

The procedures of the synthesis of SN38-PROTACs are described in Supplemental Materials and Methods.

### Antibodies

The following antibodies were used for immunoblotting: anti-Myc antibody (M4439, 9E10, ×1000 dilution, Sigma), anti-HA antibody (#3724, C29F4, ×1000 dilution, Cell Signaling Technology, Danvers, MA, USA), anti-FLAG antibody (F3165, M2, ×1000 dilution, Sigma), anti-CRBN (#71810, D8H3S, ×1000 dilution, CST), anti-Histone H3 (sc-517576, 1G1, ×500 dilution, Santa Cruz Biotechnology, Dallas, TX, USA), anti-β-actin antibody (sc-69879, AC-15, ×2000 dilution, Santa Cruz Biotechnology), anti-eIF2α antibody (#9722, ×1000 dilution,Cell Signaling Technology), anti-phospho-eIF2α S51 antibody (#3398, D9G8, ×1000 dilution, Cell Signaling Technology), anti-JNK1/2 antibody (#9252, ×1000 dilution, Cell Signaling Technology), anti-phospho-JNK1/2 antibody T183/Y185 (#9251, ×1000 dilution, Cell Signaling Technology), anti-ZAK antibody (A301-994A, ×1000 dilution, Bethyl Laboratories, Waltham, MA, USA), anti-RPL15 antibody (VPA00489KT, ×1000 dilution, Bio-Rad Laboratories, Hercules, CA, USA), anti-RPS3 antibody (#9538, D50G7, ×1000 dilution, CST), anti-p53 antibody (sc-6243, ×500 dilution, Santacruz), anti-p21 antibody (F-5, sc-6246, ×300 dilution, Santacruz), anti-TOP1 antibody (EPR5375, ×1000 dilution, Abcam, Cambridge, UK), anti-mouse IgG-HRP antibody (NA931, ×9000 dilution, Cytiva, Tokyo, Japan), and anti-rabbit IgG-HRP antibody (NA934, ×9000 dilution, Cytiva).

### Plasmid construction

RPL15 cDNA was amplified from HeLa cells' cDNA by PCR and inserted into the pCMV-Myc vector (Takara Bio Inc., Shiga, Japan). For the construction of the lentiviral expression vector of human CRBN, Flag-tagged CRBN cDNA was amplified from HeLa cells' cDNA by PCR using the primer pairs containing FLAG sequences and inserted into the plenti-CAG-IRES-EGFP vector (#69047, Addgene). An expression vector of HA-Ub was constructed by inserting human UBB cDNA into the pCMV-HA vector (Takara Bio Inc.), which was amplified from HeLa cells' cDNA using the following primer sets: 5’- ATGCAGATCTTCGTGAAAACCCTTACC-3’; 5’- TTAACAGCCACCCCTCAGGCGCAGG-3’. An expression vector of Flag-TOP1 was constructed by inserting human TOP1 cDNA into the pFlag-CMV-2 vector (Sigma), which was amplified from HeLa cells' cDNA using the following primer sets: 5’- ATGAGTGGGGACCACCTCCACAACGA-3’; 5’- CTAAAACTCATAGTCTTCATCAGCCA-3’.

### siRNA knockdown

Human CRBN siRNA (HSS121807) and ZAK siRNA (s28651) were purchased from Thermo Fisher Scientific (Waltham, MA, USA). Human RACK1 and TP53 siRNA were purchased from GenePharma (Shanghai, China). RACK1 siRNA, 5’-CCAUCAAGCUAUGGAAUACTT-3’; TP53 siRNA, 5’- GAGUGCAUUGUGAGGGUUATT-3’. Stealth RNAi siRNA Negative Control, Med GC (Thermo Fisher Scientific) was used as negative control. siRNA was transfected into MCF7 cells using Lipofectamine RNAiMAX (Thermo Scientific) by reverse transfection following the manufacturer’s instructions.

### Ubiquitination assay

HEK293T cells were transfected with Myc-RPL15 and HA-Ub expression vectors using PEI MAX (MW: 40,000, Polysciences, Warrington, PA, USA) in accordance with the manufacturer’s instructions. The cells were treated with 2 µM MG132 and 5 µM SN38-PROTAC for 12 h at 36 h post-transfection and then lysed in RIPA buffer (20 mM Tris-HCl, pH 8.0, 150 mM NaCl, 1 mM EDTA, 1% NP-40, 0.5% deoxycholate, and 0.1% SDS). Lysates were immunoprecipitated with anti-Myc antibody and analyzed by immunoblotting.

### Cell viability assay

Parent and TOP1 KO MCF7 cells were seeded into 96-well plates (1.0 × 10^4^ cells per well) and cultured overnight. The cells were treated with PROTACs at the indicated concentrations for 48 h. After treatment, 25 µl of 0.5% NP-40 and 2% Cell Titer Glo 2.0 (Promega, Madison, WI) was added, and the cells were incubated for 10 min at room temperature with gentle shaking. Next, 100 µL of samples were transferred to a well of a 96-well white plate (Greiner Bio-One, Kremsmünster, Austria) and luminescence was measured by an Infinite M200 (TECAN, Männedorf, Switzerland).

### Immunoblotting for ZAK phosphorylation

ZAK phosphorylation was detected using the Phos-tag (NARD, Kobe, Japan) following the manufacturer’s instructions. Briefly, MCF7 cells were treated with SN-38 and SN38-PROTAC at the indicated concentrations and lysed in lysis buffer (50 mM Tris-HCl, pH 8.0, 150 mM NaCl, 1% NP-40) containing protease inhibitor cocktail (EDTA-free, Nacalai), 0.5 mM PMSF, 5 µM NaF, and 1 µM vanadate. The lysate was sonicated and centrifuged, and the supernatant was applied to SDS-PAGE with a separating gel containing 7% acrylamide, 10 µM Phos-tag, and 20 µM MnCl_2_. After electrophoresis, the separating gel was washed with 10 mM EDTA for 15 min and then washed with TBS for 15 min. The protein was transferred to a PVDF membrane, and the membrane was subjected to immunoblotting.

### Nuclear and cytoplasmic fractionation

Nuclear fractions were purified from MCF7 cells using NE-PER Nuclear and Cytoplasmic Extraction Reagents (Thermo Fisher Scientific) following the manufacturer’s instructions with minor modifications. Briefly, cells were cultured in 6-well plates until they reached semi-confluence and then treated with PROTACs at the indicated concentrations. The cells were washed with hypotonic buffer (20 mM HEPES, pH 7.6, 10 mM KCl, 5 mM MgCl_2_) and then suspended in 140 µl of Cytoplasmic Extraction Reagent I containing 0.5 mM PMSF and protease inhibitor cocktail. Next, 7.7 µl of Cytoplasmic Extraction Reagent II was added to the cells with vigorous shaking. After centrifugation, the supernatant was transferred into a new 1.5 ml tube (cytoplasmic fraction). The pellet was washed three times with 300 µl of hypotonic buffer and then suspended in 70 µl of RIPA buffer containing 0.5 mM PMSF and protease inhibitor cocktail and incubated for 40 min on ice with vigorous shaking every 10 min. After centrifugation, the supernatant was transferred into a new 1.5 ml tube (nuclear fraction).

### ELISA

MCF7 cells were seeded into 96-well plates (2.0 × 10^5^ cells per well) and treated with drugs at the indicated concentrations for 48 h at 37 °C. After centrifugation, the supernatant was harvested. Next, 2.0 × 10^5^/50 µl murine BMDCs were cultured with 200 µl of the supernatant for 48 h, and IL-6 and IFNβ production was measured by ELISA (DY406-05, R&D Systems, Minneapolis, MN, and LumiKine™ Xpress mIFN-β 2.0, luex-mifnbv2, Invivogen, San Diego, CA) following the manufacturer’s instructions. Murine BMDCs were prepared as described previously [21].

### qPCR

Total RNA was purified from MCF7 cells using TRI reagent (Sigma) following the manufacturer’s instructions, and cDNA was synthesized from RNA using ReverTra Ace (TOYOBO). mRNA levels of target genes were quantified using KAPA SYBR green mix (KAPA Biosystems, Wilmington, MA, USA) with a CFX connect (Bio-Rad, Hercules, CA, USA). Primer sequences for qPCR analysis were as follows: hGAPDH forward, 5ʹ-GAAATCCCATCACCATCTTCCAGG-3ʹ; hGAPDH reverse, 5ʹ-CAGTAGAGGCAGGGATGATGTTC-3ʹ; hRPL15 forward, 5ʹ-GCAGCCATCAGGTAAGCCAAG-3ʹ; hRPL15 reverse, 5ʹ-AGCGGACCCTCAGAAGAAAGC-3ʹ; hTP53 forward, 5’- CCTCAGCATCTTATCCGAGTGG-3’; hTP53 reverse, 5’- TGGATGGTGGTACAGTCAGAGC-3’; hRACK1 forward, 5’- GCCATACCAAGGATGTGCTGAG-3’; hRACK1 reverse, 5’- CACAAGACACCCACTCTGAGTG-3’; hZAK forward, 5’- GCAGTCCAACTTGCCATTCAGAC-3’; and hZAK reverse, 5’- CCTCAGAGTATCTAACCACTGGC-3’.

### Establishment of human CRBN-expressing cell lines

For preparation of human CRBN (hCRBN)-expressing lentivirus, HEK293T cells were transfected with plenti-CAG-3×Flag-hCRBN-IRES-EGFP, psPAX2, and pMD2.G using PEI MAX. B16-F10 cells were infected with the lentivirus at 48 h post-transfection. EGFP-positive cells were sorted with a SH800 cell sorter (Sony Corporation, Tokyo, Japan) at 72 h post-infection.

### Analysis of tumor-infiltrating immune cells

hCRBN-expressing B16-F10 cells (1.0 × 10^6^ cells) were subcutaneously injected into the left flanks of 8-week-old female C57BL/6 mice. Mice were intraperitoneally administered 30 mg/kg anti-PD-1 antibody (29 F.1A12; Bio X cell) or 30 mg/kg control mouse IgG1 (MOPC-21; BioLegend) on day 9 and 15 mg/kg anti-PD-1 antibody or 15 mg/kg control mouse IgG1 on day 15. SN38-PROTACs were intraperitoneally administered 20 mg/kg on days 8, 11, and 14. Tumor-infiltrating immune cells were analyzed with a SH800 cell sorter (SONY, Tokyo, Japan)　as described previously by using following antibodies: Brilliant Violet 421 anti-CD45 antibody (103133, 30-F11, ×200 dilution, BioLegend), FITC anti-CD4 antibody (100406, GK1.5, ×100 dilution, BioLegend), APC anti-CD8a antibody (100711, 53-6.7, ×100 dilution, BioLegend), PE anti-IFNγ antibody (505807, XMG1.2, ×100 dilution, BioLegend), PE anti-FoxP3 antibody (#12-5773-80, FJK-16s, ×100 dilution, Thermo Fisher Scientific), FITC anti-Gr-1 antibody (108405, RB6-8C5, ×100 dilution, BioLegend) and APC anti-CD11b antibody (101211, M1/70, ×200 dilution, BioLegend) [22].

### Sucrose gradient sedimentation analysis

Analysis of ribosomal profiling was performed by sucrose gradient centrifugation as described previously [22].

## Results

### SN-38-conjugated pomalidomide decreases RPL15 protein levels

**Fig. 1:**
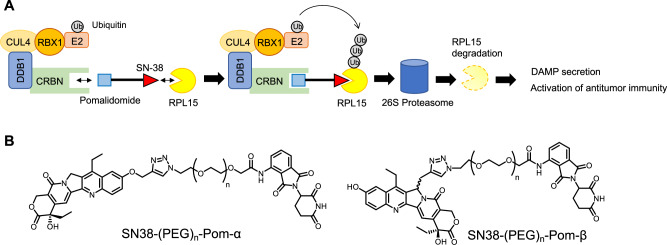
Structure of SN38-PROTACs. **A** Schematic representation of RPL15 degradation by SN38-PROTAC. **B** Structures of SN38-PROTAC-α and SN38-PROTAC-β.

Our previous study showed that camptothecin derivatives, including topotecan, SN-38, and 10-hydroxycamptothecin, bind to RPL15, and RPL15 inhibition induces DAMP secretion and activation of the antitumor immune responses [19, 22]. Therefore, in this study, we designed a PROTAC consisting of pomalidomide, which serves as the ligand for the E3 ligase CRBN, and the camptothecin derivative SN-38, with the aim to promote RPL15 ubiquitination-dependent degradation and activation of DAMP-mediated antitumor immune responses (Fig. 1A).

**Fig. 2:**
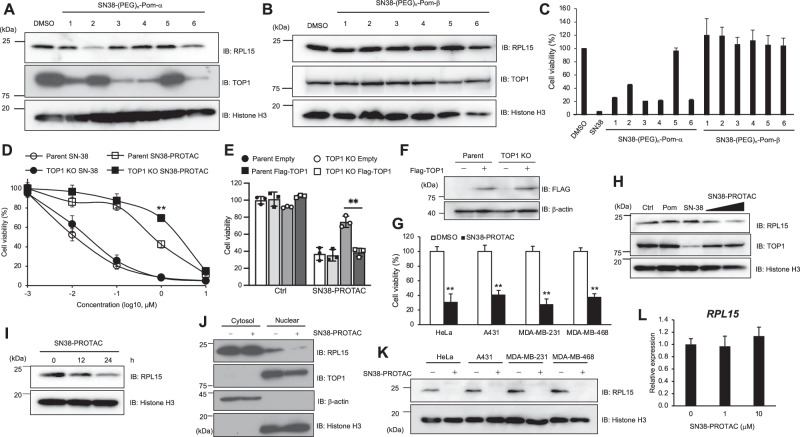
SN38-PROTAC selectively degrades nuclear RPL15. **A**, **B** MCF7 cells were treated with 10 µM SN38-PROTAC derivatives for 12 h; RPL15 and TOP1 in the nuclear lysates were detected by immunoblotting. PEG units of SN38-PROTAC derivatives were 1 to 6. Parent and TOP1 KO MCF7 cells were treated with 10 µM (**C**) or the indicated concentrations (**D**) of SN-38 and SN38-PROTAC derivatives for 48 h; cell viability was measured by CellTiter-Glo. **E** Parental and TOP1 KO MCF7 cells were transfected with an expression vector of Flag-TOP1 and treated with 1 μM SN38-PROTAC for 48 h at 48 h post-transfection. Their cell viability was measured by CellTiter-Glo. **F** Flag-TOP1 in the cell lysates of **E** were immunoblotted. **G** Various cell lines were treated with 10 µM SN38-PROTAC for 48 h, and their cell viability was measured by CellTiter-Glo. MCF7 cells were treated with **H** 1 or 5 µM SN38-PROTAC for 12 h or **I** 5 µM SN38-PROTAC for the indicated times, and immunoblotting of nuclear lysates was performed. **J** MCF7 cells were treated with 5 µM SN38-PROTAC for 12 h, and cytoplasmic and nuclear lysates were analyzed by immunoblotting. **K** Various human cell lines were treated with 10 µM SN38-PROTAC for 12 h, and nuclear lysates were analyzed by immunoblotting. **L** MCF7 cells were treated with 1 or 10 µM SN38-PROTAC for 12 h, and RPL15 mRNA expression was measured by qPCR. *n* = 3, data are shown as mean values and standard deviations. **P < 0.01, Student’s *t*-test.

We conjugated SN-38 with the alkyne-PEG linker-pomalidomide by click chemistry because 7-OH and 12-C in camptothecin derivatives have higher reactivity against azidation reagent, and topotecan has 8-CH_2_-N(CH_3_)_2_, which interferes with the conjugation with the PEG linker. We named SN-38-PEG-pomalidomide with 7-OH or 12-C conjugation as SN38-(PEG)_n_-Pom-α and SN38-(PEG)_n_-Pom-β, respectively, which have 1 to 6 PEG units (Fig. 1B). We investigated whether these compounds degrade RPL15 protein in MCF7 cells. Nuclear RPL15 was degraded by SN38-(PEG)_2_-Pom-α and SN38-(PEG)_6_-Pom-α, while TOP1 was slightly degraded by SN38-(PEG)_2_-Pom-α treatment (Fig. 2A). SN38-(PEG)_n_-Pom-β did not affect RPL15 and TOP1 levels (Fig. 2B). SN38-(PEG)_n_-Pom-β treatment did not affect cell viability in MCF7 cells (Fig. 2C). The cytotoxicity of SN38-(PEG)_n_-Pom-α and SN38-(PEG)_n_-Pom-β was largely dependent on TOP1 degradation (Fig. 2D, E). From these data, we concluded that SN38-(PEG)_2_-Pom-α has the capability to degrade RPL15 and less of a TOP1 inhibitory effect than SN-38. We thus selected SN38-(PEG)_2_-Pom-α as SN38-PROTAC.

SN38-PROTAC showed approximately 100-fold less cytotoxicity than SN-38 in parent MCF7 cells and decreased cell viability in a TOP1-dependent and -independent manner (Fig. 2D–F). Several human cell lines also showed sensitivity to SN38-PROTAC (Fig. 2G). SN38-PROTAC decreased nuclear RPL15 protein levels in a concentration- and time-dependent manner; in contrast, cytoplasmic RPL15 protein levels were not decreased by SN38-PROTAC treatment (Fig. 2H–K). RPL15 mRNA levels were not affected by SN38-PROTAC treatment (Fig. 2L). These data indicate that SN38-PROTAC selectively decreases nuclear RPL15 protein levels via a post-translational mechanism.

### SN38-PROTAC promotes RPL15 ubiquitination and its degradation, dependent on CRBN

**Fig. 3:**
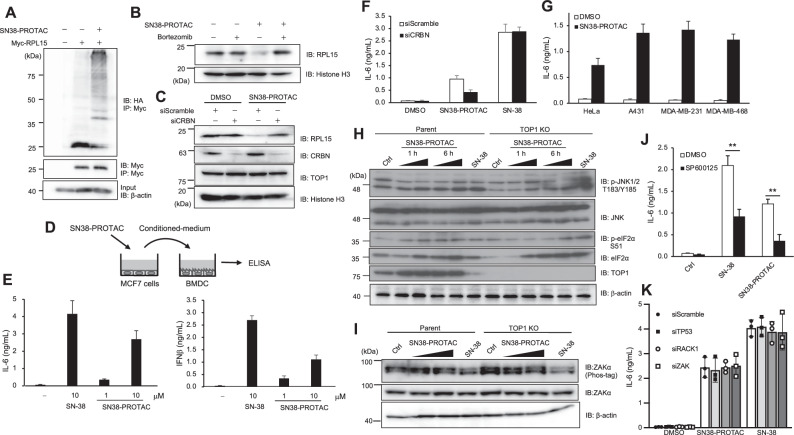
SN38-PROTAC induces RPL15 ubiquitination and JNK-dependent DAMP secretion from cancer cells. **A** HEK293T cells were transfected with Myc-RPL15 and HA-Ubiquitin vectors; at 36 h post-transfection, cells were treated with 10 µM SN38-PROTAC and 2 µM MG-132 for 12 h. The lysates were immunoprecipitated with anti-Myc antibody and analyzed by immunoblotting. **B** MCF7 cells were treated with 10 µM SN38-PROTAC and 1 µM bortezomib for 12 h, and nuclear lysates were analyzed by immunoblotting. **C** MCF7 cells were transfected with CRBN siRNA and treated with 10 µM SN38-PROTAC for 12 h at 48 h post-transfection. The nuclear lysates were analyzed by immunoblotting. **D** Schematic representation of assays evaluating DAMP secretion from SN38-PROTAC-treated MCF7 cells. **E** MCF7 cells were treated with SN-38 or SN38-PROTAC at the indicated concentrations for 48 h, and BMDCs were stimulated with MCF7 cell-conditioned medium for 48 h. IL-6 and IFNβ production from BMDCs was determined by ELISA. **F** MCF7 cells were transfected with CRBN siRNA and treated with 10 µM SN-38 or SN38-PROTAC for 48 h at 48 h post-transfection. BMDCs were stimulated as in (**D**), and cytokine production was measured by ELISA. **G** Various human cell lines were treated as in (**D**), and cytokine production was measured by ELISA. **H** Parent and TOP1 KO MCF7 cells were treated with 10 µM SN-38 for 6 h, or 1 or 10 µM SN38-PROTAC for the indicated times, and cell lysates were analyzed by immunoblotting. **I** MCF7 cells were treated as in (**G**), and phosphorylated ZAKα in the lysates was detected by immunoblotting using Phos-tag. **J** MCF7 cells were treated with 10 µM SN-38 or 10 µM SN38-PROTAC together with 1 µM SP600125 for 48 h, and BMDCs were stimulated with conditioned medium. Cytokine production was measured as in (**D**). **K** MCF7 cells were transfected with TP53, RACK1, and ZAK siRNA and then treated with 10 µM SN-38 or 10 µM SN38-PROTAC for 48 h at 48 h post-transfection. BMDCs were stimulated and their IL-6 production was measured same as **D**. *n* = 3, data are shown as mean values and standard deviations. **P < 0.01, Student’s *t*-test.

Next, we investigated whether SN38-PROTAC decreases RPL15 protein levels by promoting its ubiquitination. SN38-PROTAC promoted RPL15 ubiquitination and degraded nuclear RPL15 dependent on 26S proteasome activity (Fig. 3A, B). Degradation of nuclear RPL15 was induced by SN38-PROTAC in control MCF7 cells but not in CRBN-depleted MCF7 cells, indicating that SN38-PROTAC induces CRBN-mediated ubiquitination and degradation of nuclear RPL15 (Fig. 3C). SN38-PROTAC induced DAMP secretion from MCF7 cells similar as SN-38 (Fig. 3D, E). CRBN knockdown suppressed DAMP secretion from MCF7 cells during SN38-PROTAC treatment but not SN-38 treatment (Fig. 3F). Moreover, SN38-PROTAC induced DAMP secretion from several human cell lines (Fig. 3G).

Ribosomal stress results in rRNA processing errors, ribosome stalling during translation, and downregulation of ribosomal proteins [30–32]. We next investigated whether SN38-PROTAC activates ribosomal stress response signals. SN38-PROTAC treatment induced phosphorylation of JNK and upregulated eIF2α expression independent of TOP1 expression, similar to SN-38; ZAKα phosphorylation was decreased by SN-38 treatment but not SN38-PROTAC (Fig. 3H, I). Since phosphorylated protein moves slowly in Phos-tag-containing acrylamide gel, the upper band in the ZAKα immunoblot indicates phosphorylated ZAK protein. SN38-PROTAC inhibited 80S ribosome formation and decreased ribosomal translation efficiency (Supplementary Fig. 2A, B). SN38-PROTAC-induced DAMP secretion from MCF7 cells was decreased by treatment with SP600125, a JNK inhibitor (Fig. 3J). RNAi-mediated knockdown of p53, RACK1, and ZAK, known ribosomal stress response genes, did not affect SN38-PROTAC-induced JNK activation and DAMP secretion in MCF7 cells (Fig. 3K and Supplementary Fig. 2C–F). These data indicate that SN38-PROTAC promotes CRBN-mediated RPL15 ubiquitination and degradation, which activates JNK via unknown ribosomal stress signals and induces DAMP secretion from cancer cells independent of TOP1.

### SN38-PROTAC induces RPL15 degradation in a human CRBN-expressing murine melanoma cell line

**Fig. 4:**
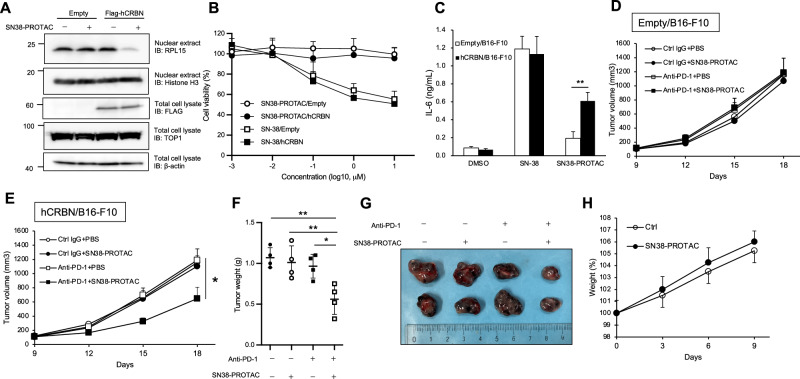
SN38-PROTAC enhances the sensitivity of B16-F10 tumors to anti-PD-1 antibody, dependent on human CRBN expression. **A** Control and FLAG-hCRBN-expressing B16-F10 cells were treated with 10 µM SN38-PROTAC for 12 h, and RPL15 was detected by immunoblotting. **B** Control- and FLAG-hCRBN-expressing B16-F10 cells were treated with SN-38 or SN38-PROTAC at the indicated concentrations for 72 h, and cell viability was measured by CellTiter-Glo. **C** Control- and FLAG-hCRBN-expressing B16-F10 cells were treated with 10 µM SN-38 or SN38-PROTAC for 72 h. BMDCs were stimulated with conditioned medium from B16-F10 cells for 48 h. IL-6 production from BMDC was determined by ELISA. **D**, **E** Control- and FLAG-hCRBN-expressing B16-F10 cells were subcutaneously inoculated into the left flank of C57BL/6 mice. Mice received i.p 20 mg/kg anti-PD-1 antibody at day 9 and 15 and 20 mg/kg SN38-PROTAC at day 8, 11, and 14. Tumor diameter was measured by calipers, and tumor volume was calculated. The mice were sacrificed at day 18, and tumor weights were measured (**F**). **G** Images of the hCRBN-expressing B16-F10 tumors at day 18. **H** C57BL/6 mice were intraperitoneally injected with 20 mg/kg SN38-PROTAC at days 0, 3, and 6, and body weight was measured. Data are shown as mean values and standard deviations. *P < 0.05, **P < 0.01, Student’s *t*-test.

We next investigate whether SN38-PROTAC-induced RPL15 degradation and DAMP secretion contribute to antitumor immune responses in the B16-F10 tumor-bearing mouse model. We prepared human FLAG-CRBN-expressing B16-F10 (hCRBN/B16-F10) cells, because a previous study showed that thalidomide and thalidomide-based PROTACs require human CRBN expression in cells for target protein degradation [33]. SN38-PROTAC degraded nuclear RPL15 in hCRBN/B16-F10 cells but not in control B16-F10 cells; TOP1 was not degraded by SN38-PROTAC treatment in hCRBN/B16-F10 cells, suggesting that SN38-PROTAC does not inhibit murine TOP1 function (Fig. 4A). SN38-PROTAC did not decrease cell viability in control and hCRBN-expressing B16-F10 cells, suggesting that cytotoxicity of SN38-PROTAC is dependent on TOP1 but not RPL15 degradation (Figs. 2D, 4B). hCRBN/B16-F10 cells treated with SN38-PROTAC secrete DAMPs, which induced IL-6 production from BMDCs (Fig. 4C).

We next evaluated whether SN38-PROTAC activated antitumor immune responses in vivo using hCRBN/B16-F10 tumor-bearing mice. Co-treatment of SN38-PROTAC and anti-PD-1 antibody significantly inhibited the growth of hCRBN/B16-F10 tumors but not control B16-F10 tumors (Fig. 4D–G). SN38-PROTAC treatment did not affect mouse bodyweight, suggesting low cytotoxicity of SN38-PROTAC in vivo (Fig. 4H). These results indicate that SN38-PROTAC induces RPL15 degradation and DAMP secretion dependent on hCRBN and enhanced antitumor effects of anti-PD-1 treatment in vivo.

### SN38-PROTAC overcomes anti-PD-1 resistance in B16-F10 tumors dependent on STING

**Fig. 5:**
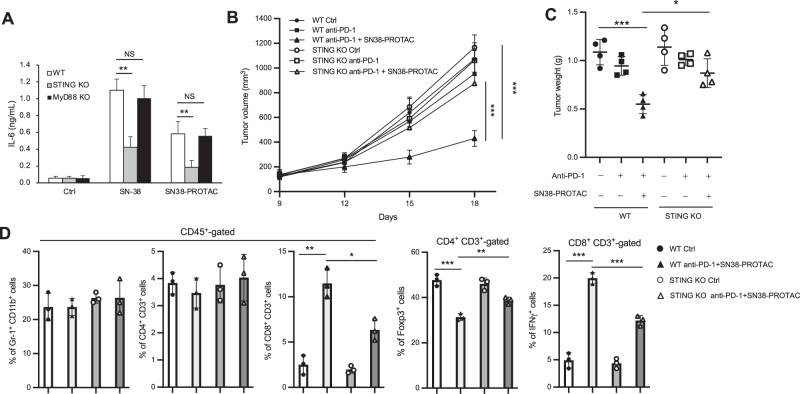
SN38-PROTAC activates STING-mediated antitumor immune responses. **A** FLAG-hCRBN-expressing B16-F10 cells were treated with 10 µM SN-38 or SN38-PROTAC for 72 h; BMDCs from WT, STING KO, and MyD88 KO mice were stimulated with conditioned medium from the B16-F10 cells for 48 h, and IL-6 production was determined by ELISA (*n* = 3). **B**, **C** FLAG-hCRBN-expressing B16-F10 cells were subcutaneously inoculated into the left flank of WT and STING KO C57BL/6 mice; the mice received i.p 20 mg/kg anti-PD-1 antibody at day 9 and 15 and 20 mg/kg SN38-PROTAC at day 8, 11, and 14. Tumor diameter was measured by caliper, and tumor volume was calculated. The mice were sacrificed at day 18, and tumor weight was measured (*n* = 4). **D** Viable CD45+ tumor-infiltrating cells were gated by the indicated markers and analyzed by flow cytometry on day 18 (*n* = 3). Data are shown as mean values and standard deviations. *P < 0.05, **P < 0.01, ***P < 0.001, Student’s *t*-test.

Finally, we investigated whether SN38-PROTAC activation of antitumor immune responses depends on STING. SN38-PROTAC treatment induced DAMP secretion from hCRBN/B16-F10 cells, which activated BMDCs dependent on STING but not MyD88 (Fig. 5A). Co-treatment of SN38-PROTAC and anti-PD-1 antibody inhibited the growth of hCRBN/B16-F10 tumors in wild-type (WT) mice but not STING KO mice (Fig. 5B, C). Infiltration of IFNγ + CD8 + T cells into hCRBN/B16-F10 tumors was increased and Foxp3 + CD4 + T cells (Treg) infiltration to tumors was decreased by co-treatment of SN38-PROTAC and anti-PD-1 antibody compared with only anti-PD-1 antibody treatment; these results were not observed in STING KO mice (Fig. 5D, Supplementary Fig. 3). These results indicates that SN38-PROTAC overcomes anti-PD-1 resistance of melanoma via activation of STING signaling.

## Discussion

Over the last two decades, various PROTACs have been developed as anticancer strategies to induce the degradation of cancer-related factors, immune signaling proteins, and epigenetic-related proteins [25, 34–36]. These PROTACs suppress cancer growth by inhibiting cell proliferation or activating antitumor immune responses. For example, Kolb et al. showed that the BCL-xL degrader PROTAC activates CD8 + T cell-mediated antitumor immune responses via depleting tumor-infiltrating Treg, suggesting that the combination of anticancer PROTACs and ICI may improve cancer treatment [37]. In this study, we developed a novel anticancer PROTAC that activates STING-dependent antitumor immune responses via DAMP secretion from cancer cells and sensitizes B16-F10 tumors to anti-PD-1 antibody treatment.

Several studies showed that the TOP1 inhibitor irinotecan enhanced the antitumor activity of the anti-PD-1 antibody. Our previous study showed that topotecan and camptothecin derivatives inhibit RPL15 function, and this inhibition sensitized B16-F10 tumors to anti-PD-1 antibody treatment [22, 38–40]. Thus, co-treatment of anti-PD-1 antibody and camptothecin derivatives improved therapeutic efficacy in anti-PD-1-resistant tumors. However, camptothecin derivatives have high cytotoxicity because of the inhibitory effect on TOP1 and cause side effects in clinical treatment, such as diarrhea and bone marrow suppression. SN38-PROTAC has the potential to overcome these issues. SN38-PROTAC enhanced the antitumor effects of anti-PD-1 antibody; it showed less cytotoxicity than SN-38 in human cell lines and did not affect mouse body weight in tumor models (Figs. 2D and 4H). We speculate that the reason why SN38-PROTAC treatment alone did not affect tumor growth is because of its low cytotoxicity (Figs. 2D and 4B). Moreover, our results showed that SN38-PROTAC induced DAMP secretion from various human cancer cell lines, not only melanoma cells, suggesting that SN38-PROTAC may act as an “enhancer” of ICI treatment in other tumor models (Fig. 3G). We plan to investigate whether SN38-PROTAC improves the sensitivity of anti-PD-1 antibody with less cytotoxicity in various anti-PD-1-resistant cancers, such as colon cancer and ovarian cancer.

Eukaryotic ribosomal proteins assemble into 40S and 60S ribosomal subunits with ribosomal RNA in the nucleolus; the subunits translocate to the cytoplasm and form the 80S ribosome. While ribosomal proteins exist as non-assembled forms or in a ribosomal protein complex in the nucleus, non-assembled ribosomal proteins were not detected in the cytosolic fraction in our sucrose gradient sedimentation analysis (Supplementary Fig. 2A). Our previous study showed that topotecan inhibits 60S subunit formation in the nucleus independent of TOP1 [22]. We found that SN38-PROTAC degraded nuclear RPL15 protein but not cytoplasmic RPL15 (Fig. 2J). These data suggest that SN38-PROTAC binds to non-assembled RPL15 in the nucleus but not cytoplasmic RPL15 in the 60S and 80S ribosomes, resulting in the induction of JNK and eIF2a phosphorylation, which are some of the ribosomal stress response signals. Our data showed that JNK inhibition decreased DAMP secretion from SN-38- or SN38-PROTAC-treated MCF7 cells (Fig. 3J). Gan et al. showed that lipotoxicity induces mitochondrial injury and HMGB1 secretion as DAMP in hepatocytes, dependent on JNK activation [41]. These studies suggest that JNK activation links ribosomal stress to mitochondrial injury and DAMP secretion, which increases the sensitivity of ICI in cancer. Supporting this idea, some studies showed that JNK activation improved the sensitivity to anti-PD-1 therapy in pancreatic and small lung cancer; however, other studies reported that JNK activation increases anti-PD-1 resistance by upregulating PD-L1 expression in glioblastoma and bladder cancer [42–45]. Further investigation on JNK contribution to ICI therapy and DAMP-mediated antitumor immune responses is required.

The expression levels of CRBN correlate with the sensitivity of anticancer drugs in various cancers. Zhang et al. showed that the objective response rate of antitumor therapy is inversely proportional to CRBN expression in several cancers, and multiple myeloma cells with loss of CRBN expression acquire resistance to lenalidomide and pomalidomide treatment [46, 47]. This suggests cancer cells become resistant to SN38-PROTAC because hCRBN expression is required for RPL15 degradation and DAMP-mediated STING activation (Figs. 4A, C, and 5A). Loss of CRBN expression in cancer cells is a common problem for CRBN-recruited PROTACs; downregulation of CRBN upregulates AMPK activity, suggesting acceleration of immunogenic cell death in these cancer cells [48–50]. Supporting this idea, a previous study showed that the expression level of CRBN correlates with the infiltration of immune cells in several tumors [46]. Antitumor immune activation with CRBN-recruited PROTACs has the possibility to overcome resistance to ICI therapy of cancers.

## Supplementary information

Supplementary figure

Supplementary methods

## Data Availability

All data are contained within the manuscript.
