# Supplementary material for: A selective RPL15 PROTAC degrader enhances anti-PD-1 immunotherapy in a murine melanoma tumor model
Source: Oncogene. 2025 Nov 23;44(50):4846–54. doi: 10.1038/s41388-025-03641-4 (PMC12669025; doi:10.1038/s41388-025-03641-4)
Supplement: Supplementary file 1 — Supplementary figure [file 41388_2025_3641_MOESM1_ESM.pdf]

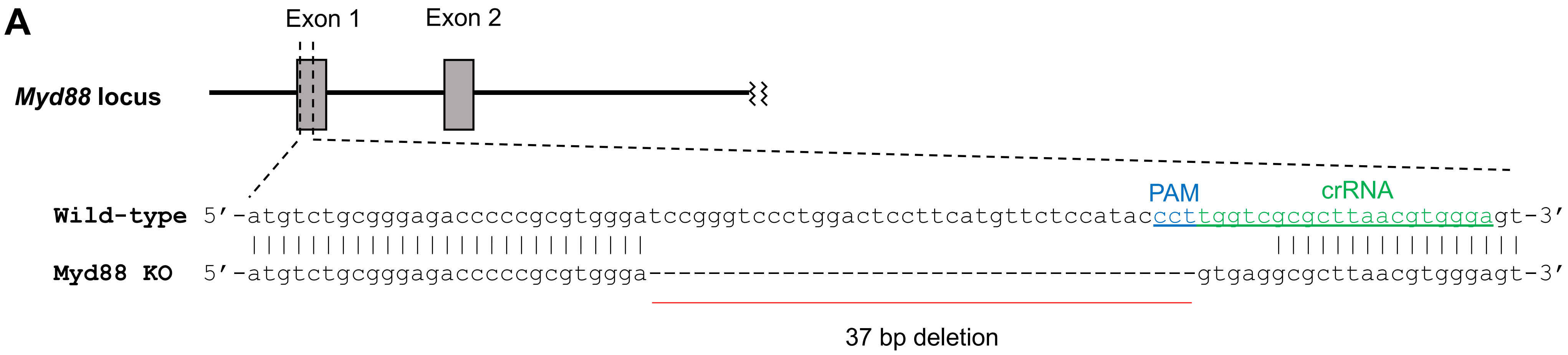

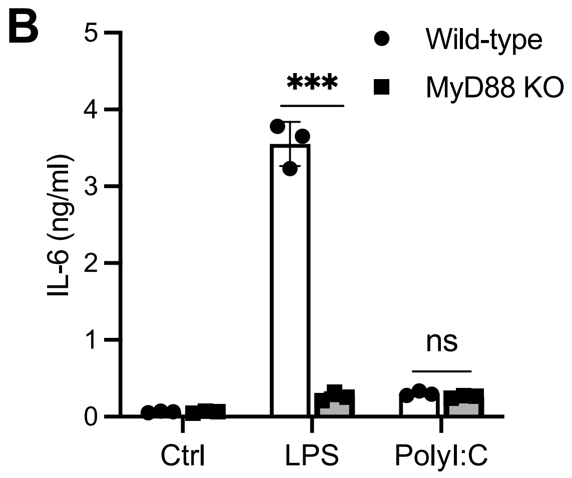

Supplementary figure 1. Construction of MyD88-deficient mice

(A) Schematic representation of the Myd88 gene locus in MyD88-deficient mice. (B) BMDCs from WT and MyD88 KO mice were stimulated with 100 ng/ml of LPS or 1 μg/ml of liposome-coated polyI:C for 24 h, and IL-6 production was measured by ELISA. n = 3, data are shown as mean values and standard deviations. ***P < 0.001, Student's t-test.

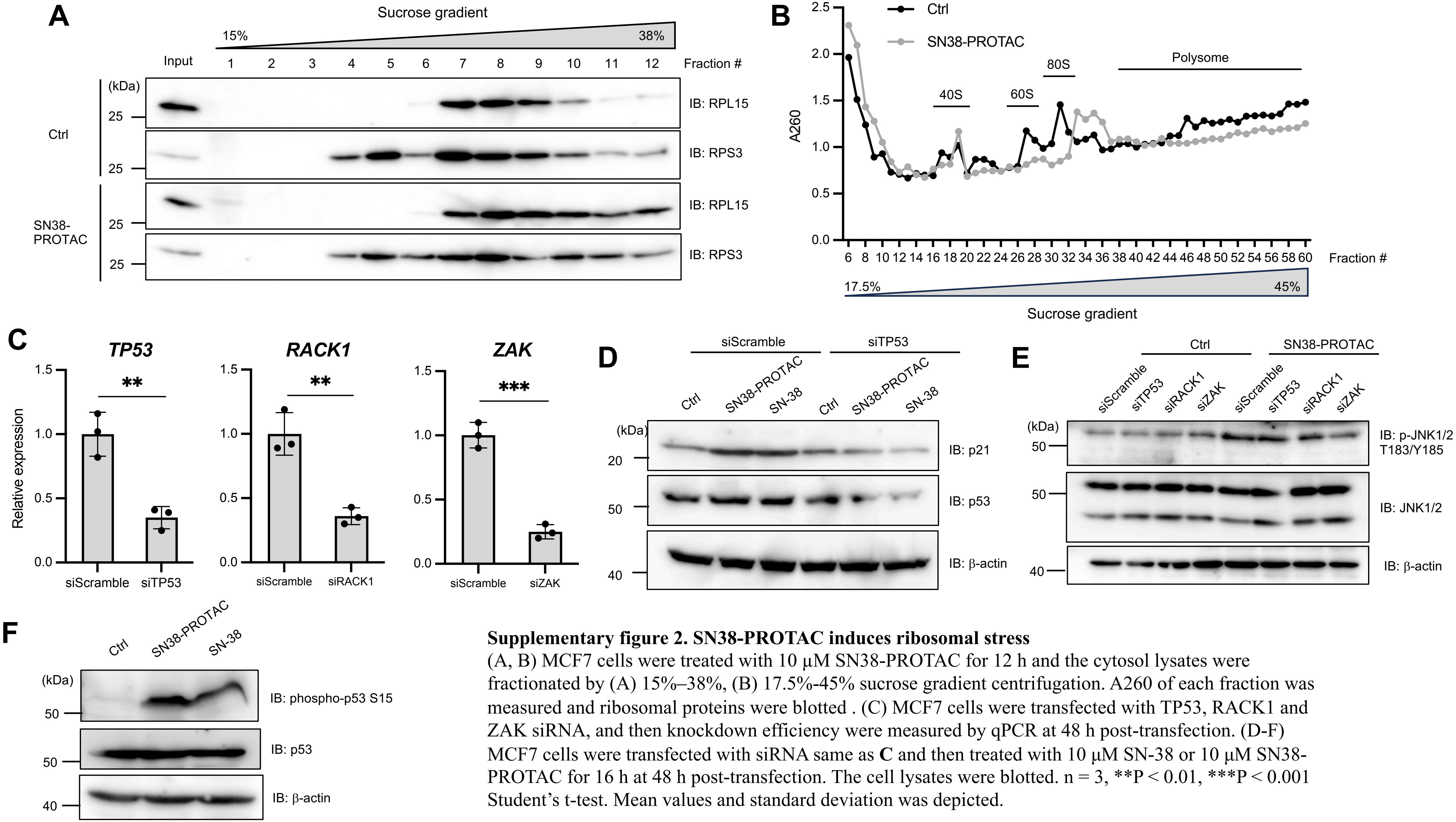

Supplementary figure 2. SN38-PROTAC induces ribosomal stress

(A, B) MCF7 cells were treated with 10 μM SN38-PROTAC for 12 h and the cytosol lysates were fractionated by (A) 15%–38%, (B) 17.5%-45% sucrose gradient centrifugation. A260 of each fraction was measured and ribosomal proteins were blotted . (C) MCF7 cells were transfected with TP53, RACK1 and ZAK siRNA, and then knockdown efficiency were measured by qPCR at 48 h post-transfection. (D-F) MCF7 cells were transfected with siRNA same as **C** and then treated with 10 μM SN-38 or 10 μM SN38-PROTAC for 16 h at 48 h post-transfection. The cell lysates were blotted. n = 3, $**P < 0.01$, $***P < 0.001$ Student’s t-test. Mean values and standard deviation was depicted.

Supplementary figure 3

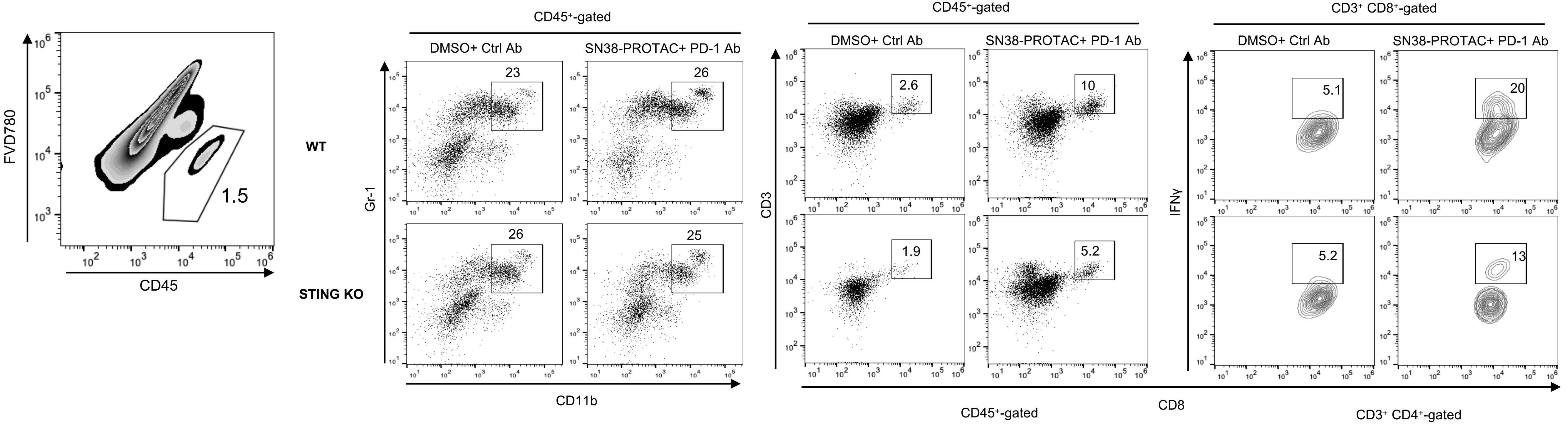

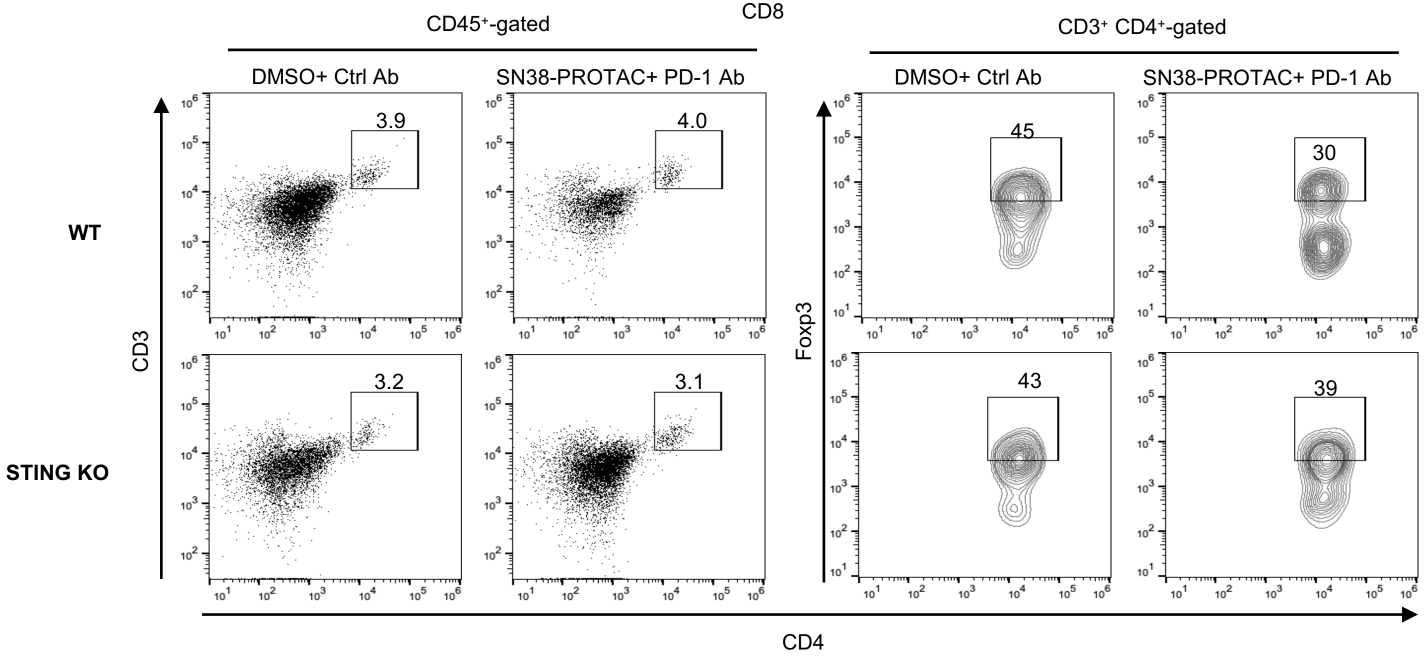

Supplementary figure 3. Flow cytometry analysis for tumor-infiltrating immune cells.

The gating strategy of FACS for Figure 5D is shown. Viable leucocytes in the tumor were gated by FVD780− CD45+ populations, and Foxp3 and IFNγ expression in T cells and myeloid-derived suppressor cells were quantified by FACS.
