# Supplementary material for: A selective RPL15 PROTAC degrader enhances anti-PD-1 immunotherapy in a murine melanoma tumor model
Source: Oncogene. 2025 Nov 23;44(50):4846–54. doi: 10.1038/s41388-025-03641-4 (PMC12669025; doi:10.1038/s41388-025-03641-4)

Supplemental methods

Synthesis of SN38-(PEG)$_n$-Pomalidomide

All reactions except those carried out in the aqueous phase were performed under an argon atmosphere unless otherwise noted. Materials were purchased from commercial suppliers and used without further purification unless otherwise noted. Solvents were distilled according to the standard protocol. Isolated yields were calculated by weighing products. The weight of the starting materials and the products were not calibrated. Analytical thin layer chromatography (TLC) was performed on Merck silica gel 60F_{254} plates. Normal-phase column chromatography was performed on Merck silica gel 5715 or Wakogel 60N. Flash column chromatography was performed on Kanto Chemical Silica Gel 60N (spherical, neutral. 40-50 µm). ^{1}H NMR was measured in $CDCl_3$, DMSO-d_6, or methanol-d_4 solution, and reported in parts per million (δ) relative to tetramethylsilane (0.00 ppm) as an internal standard using JEOL ECS400, ECX400, ECZ400, ECA500, unless otherwise noted. ^{13}C NMR was measured in $CDCl_3$, DMSO-d_6, or methanol-d_4 solution, and referenced to residual solvent peaks of $CDCl_3$ (77.16 ppm) or methanol-d_4 (49.00 ppm) using JEOL ECS400, ECX400, ECZ400, ECA500. Coupling constant (*J*) was reported in hertz (Hz). Abbreviations of multiplicity were as follows; s: singlet, d: doublet, t: triplet, q: quartet, m: multiplet, br: broad. Data were presented as follows; chemical shift (multiplicity, integration, coupling constant). The assignment was based on ^{1}H-^{1}H COSY spectra. Mass spectra were obtained on Waters MICRO MASS LCT-premier and the mass analyzer type used for the HRMS measurements was TOF. Optical rotation was measured on a Rudolph Research Analytical Autopol IV automatic polarimeter.

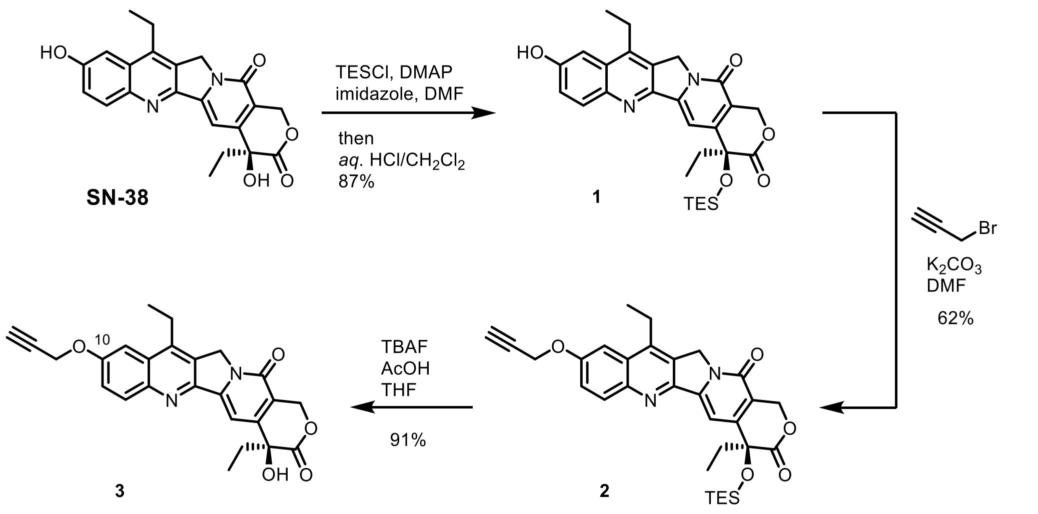

Scheme 1. Synthesis of 10-*O*-propargyl SN-38 (**3**)

TES protection [13-38-1] **Compound 1**

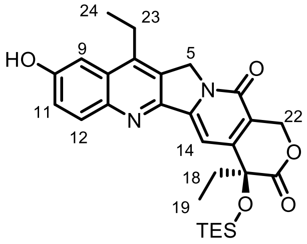

A suspension of SN-38 (100 mg, 0.255 mmol, 1.0 equiv.) in DMF (5 mL) was treated with imidazole (87.1 mg, 1.28 mmol, 5 equiv.), DMAP (31.0 mg, 0.255 mmol, 1 equiv.), and TESCl (171 μL, 1.02 mmol, 4 equiv.) at room temperature. After 20 h, imidazole (87.1 mg) and TESCl (171 μL) were added to the reaction mixture. After 20 h, imidazole (87.1 mg) and TESCl (171 μL) were added again to the reaction mixture. After 7 h, imidazole (87.1 mg) and TESCl (171 μL) were added again to the reaction mixture. Consuming starting material, the reaction mixture was partitioned between CH_2Cl_2 and H_2O. the organic layer was washed with *sat. aq*. NH_4Cl and brine, dried (Na_2SO_4), filtered and concentrated *in vacuo*. The crude compound was treated with 0.1 M *aq*. HCl and CH_2Cl_2 (v/v 1/1, 1 mL) at room temperature. According to TLC analysis, bis-TES ether was consumed, and new spot was appeared. The reaction mixture was partitioned between CH_2Cl_2 and 0.1 M *aq*. HCl. The organic layer was washed with brine, dried (Na_2SO_4), filtered, and concentrated *in vacuo*. The residue was purified by silica gel column chromatography (ϕ2.1×9 cm; hexane/EtOAc = 1/4 → 1/9) to afford **1** (113 mg, 0.223 mmol, 87%) as a pale yellow solid. This is a known compound (WO 2008/011994).

^{1}H NMR (400 MHz, $CDCl_3$) δ 8.16 (d, 1H, H-12, $J_{12,11}$ = 9.0 Hz), 7.54 (s, 1H, H-14), 7.47-7.39 (m, 3H, H-9, H-11, OH), 5.70 (d, 1H, H-22, J_{gem} = 16.6 Hz), 5.27 (d, 1H, H-22, J_{gem} = 16.6 Hz), 5.25 (s, 2H, H-5), 3.08 (q, 2H, H-23, $J_{23,24}$ = 7.6 Hz), 1.98-1.83 (m, 2H, H-19), 1.35 (t, 3H, H-24, $J_{24,23}$ = 7.6 Hz), 1.01-0.91 (m, 12H, H-18, C$H_3$$CH_2$Si×3), 0.77-0.71 (m, 6H, CH_3CH_2Si×3); LRMS-ESI 529.4, calcd. 529.2 [$(M+Na)^+$].

Propargylation [13-39-1] **Compound 2**

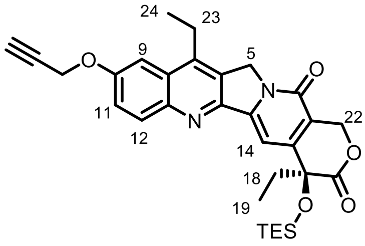

A solution of **1** (50 mg, 98.7 μmol, 1.0 equiv.) in DMF (1 mL) was treated with K_2CO_3 (16.4 mg, 118 μmol, 1.2 equiv.) and propargyl bromide (11.8 μL, 109 μmol, 1.1 equiv.) at room temperature for 6 h. Propargyl bromide (5.5 μL) was added to the reaction mixture. After 3 h, the reaction was quenched with 1 M *aq*. HCL at 0 °C, and the resulting mixture was extracted with hexane/EtOAc (v/v 1/4), and the organic layer was washed with H_2O and brine, dried (Na_2SO_4), filtered, and concentrated *in vacuo*. The residue was purified by Hi-Flash silica gel column chromatography (M size: ϕ1.9×7 cm; EtOAc/hexane = 77% → 98%) to afford **2** (33.4 mg, 61.3 μmol, 62%) as a pale yellow solid.

^{1}H NMR (400 MHz, $CDCl_3$) δ 8.19 (m, 1H, H-12), 7.52-7.49 (m, 3H, H-9, H-11, H-14), 5.68 (d, 1H, H-22, J_{gem} = 16.5 Hz), 5.26 (d, 1H, H-22, J_{gem} = 16.5 Hz), 4.90 (d, 2H, C≡CCH_2O, J = 2.3 Hz), 3.16 (q, 2H, H-23, $J_{23,24}$ = 7.6 Hz), 2.60 (t, 1H, CH≡CCH_2O, J = 2.3 Hz), 1.99-1.85 (m, 2H, H-19), 1.41 (t,

3H, H-24, $J_{24,23}$ = 7.6 Hz), 1.01-0.95 (m, 12H, H-18, CH_3CH$_2$Si×3), 0.79-0.71 (m, 6H, CH$_3$CH_2Si×3); LRMS-ESI 545.5, calcd. 545.2 [(M+H)$^+$].

Deprotection [13-43-1] **Compound 3**

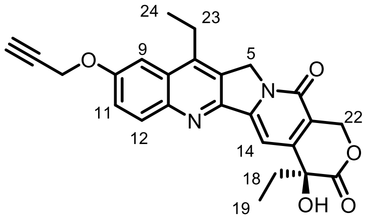

A solution of **2** (33.4 mg, 61.3 μmol, 1.0 equiv.) in THF (1 mL) was treated with AcOH (5.26 μL, 92.0 μmol, 1.5 equiv.) and TBAF (1 M in THF, 73.6 μL, 73.6 μmol, 1.2 equiv.) at room temperature for 30 min. To the reaction mixture was added THF (1 mL). After 1.5 h, the reaction mixture was concentrated *in vacuo*, and the residue was partitioned between 10% MeOH/CH$_2$Cl$_2$ and *sat. aq.* NaHCO$_3$. The organic layer was washed with brine×2, dried (Na$_2$SO$_4$), filtered, and concentrated *in vacuo*. The residue was purified by silica gel column chromatography (ϕ1.1×9 cm; MeOH/CHCl$_3$ = 1% → 2%) to afford **3** (24.0 mg, 55.8 μmol, 91%) as a pale yellow solid.

^{1}H NMR (400 MHz, DMSO-d_6) δ 8.10 (d, 1H, H-12, $J_{12,11}$ = 9.2 Hz), 7.62 (d, 1H, H-9, $J_{9.11}$ = 2.7 Hz), 7.54 (dd, 1H, H-11, $J_{11,12}$ = 9.2, $J_{11,9}$ = 2.7 Hz), 7.28 (s, 1H, H-14), 6.50 (s, 1H, OH), 5.43 (s, 2H, H-5 or H-22), 5.31 (s, 2H, H-5 or H-22), 5.08 (d, 2H, C≡CCH_2O, J = 2.3 Hz), 3.66 (t, 1H, CH≡CCH$_2$O, J = 2.3 Hz), 3.19 (q, 2H, H-23, $J_{23,24}$ = 7.6 Hz), 1.94-1.79 (m, 2H, H-19), 1.33 (t, 3H, H-24, $J_{24,23}$ = 7.6 Hz), 0.88 (t, 3H, H-18, $J_{18,19}$ = 7.3 Hz); LRMS-ESI 431.2, calcd. 431.2 [(M+H)$^+$].

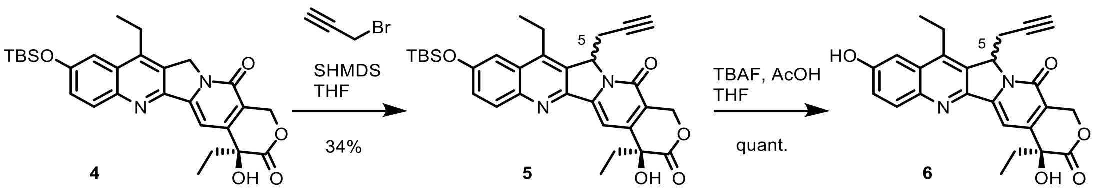

Scheme 2. Synthesis of 5-*C*-propargyl SN-38 (**6**)

Propargylation [13-82-1] **Compound 5**

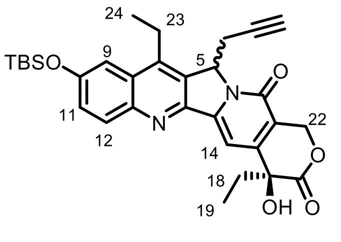

A solution of **4** [Moon, S.-J. *et al. J. Med. Chem.* **2008**, *51*, 6916-6926] (50 mg, 98.7 μmol, 1.0 equiv.) in THF (1 mL) was treated with SHMDS (1.9 M in THF, 57.1 μL, 109 μmol, 1.1 equiv.) at 0 °C for 5 min. Propargyl bromide (12.9 μL, 118 μmol, 1.2 equiv.) was added to the reaction mixture at 0 °C, and then the reaction mixture was warmed at room temperature and stirred for 5 h. The reaction mixture was partitioned between EtOAc and 0.1 M *aq*. HCl. The organic layer was washed with brine, dried (Na$_2$SO$_4$), filtered, and concentrated *in vacuo*. The residue was purified by Hi-Flash silica gel column chromatography (S size: ϕ1.4×6.5 cm (tandem); EtOAc/hexane = 67% → 87%) to afford **5** (18.1 mg, 33.2 μmol, 34%) as a pale yellow solid.

^{1}H NMR (400 MHz, $CDCl_3$) δ 8.11 (d, 1H, H-12, $J_{12,11}$ = 9.1 Hz), 7.56, 7.54 (each s, 1H, H-9), 7.40-7.36 (m, 2H, H-11, H-14), 5.88, 5.86 (each t, 1H, H-5, J = 3.4 Hz), 5.72, 5.71 (each d, 1H, H-22, J_{gem} = 16.3 Hz), 5.31, 5.26 (each d, 1H, H-22, J_{gem} = 16.3 Hz), 4.13-4.04 (m, 1H, C≡CCH_2), 3.24-3.14 (m, 1H, H-23), 3.11-2.97 (m, 2H, H-23, C≡CCH_2), 1.99-1.82 (m, 2H, H-19), 1.55, 1.54 (each t, 1H, CH≡CH_2, J = 2.4 Hz), 1.44 (t, 3H, H-24, $J_{24,23}$ = 7.5 Hz), 1.05 (s, 9H, ^{t}BuSi), 1.05-1.01 (m, 3H, H-18), 0.32 (s, 6H, MeSi×2); LRMS-ESI 545.3, calcd. 545.2 [$(M+H)^+$].

Deprotection [13-86-1] **Compound 6**

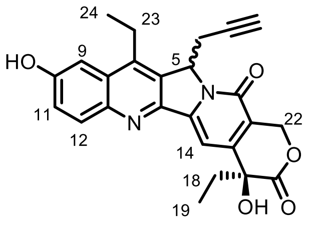

A solution of **5** (18.0 mg, 33.0 μmol, 1.0 equiv.) in THF (1 mL) was treated with AcOH (2.83 μL, 49.6 μmol, 1.5 equiv.) and TBAF (1 M in THF, 39.7 μL, 39.7 μmol, 1.2 equiv.) at room temperature for 15 min. The reaction mixture was concentrated *in vacuo*, and the residue was purified by silica gel column chromatography (ϕ1.1×7 cm; MeOH/$CHCl_3$ = 1% → 2% → 3%) to afford **6** (14.2 mg, 33.0 mmol, quant.) as a pale yellow solid.
^{1}H NMR (400 MHz, DMSO-d_6) δ 10.32 (s, 1H, OH), 8.01 (d, 1H, H-12, $J_{12,11}$ = 9.9 Hz), 7.43-7.04 (m, 2H, H-9, H-11), 7.21, 7.20 (each s, 1H, H-14), 6.51, 6.49 (each s, 1H, OH), 6.07, 6.05 (each t, 1H, H-5, J = 3.4 Hz), 5.49-5.35 (m, 2H, H-22), 3.92, 3.88 (each dt, 1H, C≡CCH_2, J_{gem} = 11.9, J = 3.1 Hz), 3.19-3.05 (m, 3H, H-23, C≡CCH_2), 2.46, 2.45 (each t, 1H, CH≡CH_2, J = 3.1 Hz), 1.96-1.79 (m, 2H, H-19), 1.31 (t, 3H, H-24, $J_{24,23}$ = 7.4 Hz), 0.86 (t, 3H, H-18, $J_{18,19}$ = 7.2 Hz); LRMS-ESI 431.2, calcd. 431.2 [$(M+H)^+$].

General Procedure (CuAAC)

For example, scale-up synthesis of SN38-$(PEG)_2$-Pom-α was described. A solution of alkyne **3** (15.0 mg, 34.8 μmol), pomalidomide-PEG2-azide (17.0 mg, 38.3 μmol), and *i*-Pr_2NEt (11.8 μL, 69.6 μmol) in THF/DMSO (v/v 10/1, 1 mL) was treated with CuI (0.7 mg, 3.48 μmol) at room temperature for 18 h. The mixture was diluted with CH_2Cl_2, and the mixture was washed with *sat. aq*. NH_4Cl/*sat. aq*. $NaHCO_3$ (v/v 3/1) and brine. The organic layer was dried (Na_2SO_4), filtered, and concentrated *in vacuo*. The residue was purified by preparative TLC (8% MeOH/$CHCl_3$) to afford SN38-$(PEG)_2$-Pom-α (29.9 mg, 98%) as a pale yellow solid.

SN38-$(PEG)_1$-Pom-α

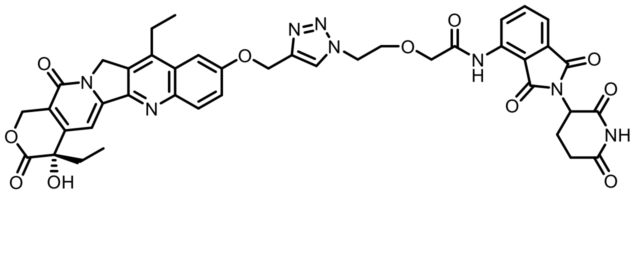

According to general procedure, alkyne **3** (5.0 mg, 11.6 μmol) and pomalidomide-PEG1-azide (6.0 mg) were used to afford SN38-(PEG)$_1$-Pom-α (1.9 mg, 20%) as a pale yellow solid.

^{1}H NMR (400 MHz, $CDCl_3$) δ 10.36, 10.35 (each s, 1H, amide-N*H*), 8.79, 8.78 (each d, 1H, H-7′, $J_{7',6'}$ = 8.4 Hz), 8.54 (br s, 1H, imide-N*H*), 8.17, 8.16 (each s, 1H, triazole-C*H*), 8.04 (d, 1H, H-12, $J_{12,11}$ = 9.2 Hz), 7.72 (dd, 1H, H-6′, $J_{6',5'}$ = $J_{6',7'}$ = 8.0 Hz), 7.66 (t, 1H, H-9, $J_{9,11}$ = $J_{9,12}$ = 2.8 Hz), 7.59 (d, 1H, H-5′, $J_{5',6'}$ = 7.6), 7.56 (s, 1H, H-14), 7.52 (dd, 1H, H-11, $J_{11,12}$ = 9.2, $J_{11,9}$ = 2.8 Hz), 5.75 (d, 1H, H-22, J_{gem} = 16.4 Hz), 5.30 (s, 2H, H-5), 5.29 (d, 1H, H-22, J_{gem} = 16.4 Hz), 5.20 (s, 2H, triazole-CH_2-O), 5.00-4.95 (m, 1H, H-11′), 4.81-4.69 (m, 2H, O-CH_2-CON), 4.15-4.10 (m, 2H, CH_2), 4.07-4.00 (m, 2H, CH_2), 3.77, 3.76 (each s, 1H, OH), 3.12 (q, 2H, H-23, $J_{23,24}$ = 7.6 Hz), 2.94-2.74 (m, 3H, H-12′, H-13′), 2.26-2.18 (m, 1H, H-12′), 1.95-1.82 (m, 2H, H-19), 1.37 (t, 3H, H-24, $J_{24,23}$ = 7.6 Hz), 1.03 (t, 3H, H-18, $J_{18,19}$ = 7.4 Hz); LCMS-ESI 831, calcd. 831.3 [$(M+H)^+$].

SN38-(PEG)$_2$-Pom-α

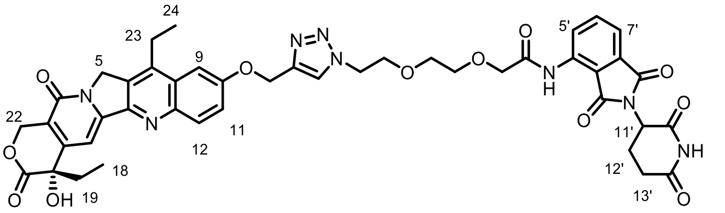

Mentioned at general procedure.

^{1}H NMR (400 MHz, $CDCl_3$) δ 10.37, 10.36 (each s, 1H, amide-N*H*), 8.80 (d, 1H, H-7′, $J_{7',6'}$ = 8.4 Hz), 8.55 (br s, 1H, imide-N*H*), 8.07 (d, 1H, H-12, $J_{12,11}$ = 9.5 Hz), 7.86 (s, 1H, triazole-C*H*), 7.66 (dd, 1H, H-6′, $J_{6',5'}$ = $J_{6',7'}$ = 7.7 Hz), 7.57 (d, 1H, H-9, $J_{9,11}$ = 0.9 Hz), 7.51 (dd, 1H, H-5′, $J_{5',6'}$ = 8.4, $J_{5',7'}$ = 0.9 Hz), 7.47 (br s, 1H, H-14), 7.41 (dd, 1H, H-11, $J_{11,12}$ = 9.1, $J_{11,9}$ = 2.7 Hz), 5.73 (d, 1H, H-22, J_{gem} = 16.1 Hz), 5.32 (s, 2H, H-5), 5.28 (d, 1H, H-22, J_{gem} = 16.1 Hz), 5.20 (s, 2H, triazole-CH_2-O), 4.96 (dd, 1H, H-11′, $J_{11',12'}$ = 12.7, $J_{11',12'}$ = 5.4 Hz), 4.64-4.52 (m, 2H, O-CH_2-CON), 4.13 (d, 1H, CH_2, J_{gem} = 15.4 Hz), 4.09 (d, 1H, CH_2, J_{gem} = 15.4 Hz), 4.02-3.93 (m, 2H, CH_2), 3.87 (s, 1H, OH), 3.77-3.72 (m, 4H, CH_2×2), 3.12 (q, 2H, H-23, $J_{23,24}$ = 7.7 Hz), 2.94-2.70 (m, 3H, H-12′, H-13′), 2.23-2.16 (m, 1H, H-12′), 1.94-1.82 (m, 2H, H-19), 1.36 (t, 3H, H-24, $J_{24,23}$ = 7.7 Hz), 1.03 (t, 3H, H-18, $J_{18,19}$ = 7.3 Hz); LCMS-ESI 875, calcd. 875.3 [$(M+H)^+$].

SN38-(PEG)$_3$-Pom-α

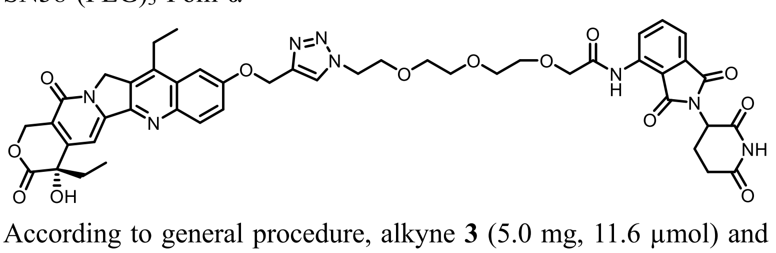

According to general procedure, alkyne **3** (5.0 mg, 11.6 μmol) and pomalidomide-PEG3-azide (6.0

mg) were used to afford SN38-(PEG)$_3$-Pom-α (1.7 mg, 16%) as a pale yellow solid.

^{1}H NMR (400 MHz, $CDCl_3$) δ 10.36, 10.34 (each d, 1H, amide-N*H*), 8.79. 8.78 (each d, 1H, H-7′, $J_{7',6'}$ = 8.6 Hz), 8.37 (br s, 1H, imide-N*H*), 8.10 (dd, 1H, H-12, $J_{12,11}$ = 9.4, $J_{12,9}$ = 1.4 Hz), 7.92, 7.91 (each s, 1H, triazole-C*H*), 7.69-7.64 (m, 1H, H-6′), 7.57 (m, 4H, H-9, H-11, H-14, H-5′), 5.74 (d, 1H, H-22, J_{gem} = 16.4 Hz), 5.40 (s, 2H, H-5), 5.30 (d, 1H, H-22, J_{gem} = 16.4 Hz), 5.22, 5.21 (each s, 2H, triazole-C*H*$_2$-O), 4.97-4.92 (m, 1H, H-11′), 4.56 (t, 2H, O-C*H*$_2$-CON, *J* = 4.8 Hz), 4.17 (s, 2H, C*H*$_2$), 3.88 (t, 2H, C*H*$_2$, *J* = 5.0 Hz), 3.80-3.70 (m, 4H, C*H*$_2$×2), 3.59 (s, 2H, C*H*$_2$×2), 3.12 (q, 2H, H-23, $J_{23,24}$ = 7.8 Hz), 2.94-2.74 (m, 3H, H-12′, H-13′), 2.24-2.17 (m, 1H, H-12′), 1.95-1.84 (m, 2H, H-19), 1.37 (t, 3H, H-24, $J_{24,23}$ = 7.8 Hz), 1.04 (t, 3H, H-18, $J_{18,19}$ = 7.4 Hz); LCMS-ESI 919, calcd. 919.3 [$(M+H)^+$].

SN38-(PEG)$_4$-Pom-α

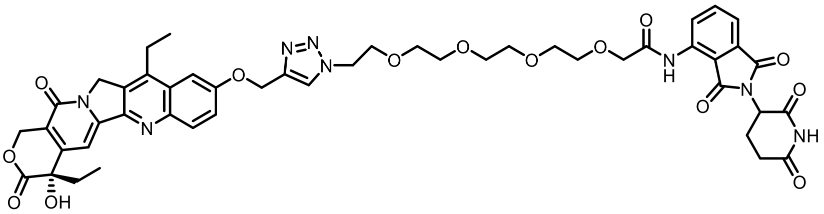

According to general procedure, alkyne **3** (20 mg, 45.3 μmol) and pomalidomide-PEG4-azide (50 mg) were used to afford SN38-(PEG)$_4$-Pom-α (18.0 mg, 41%) as a pale yellow solid.

^{1}H NMR (400 MHz, $CDCl_3$) δ 10.42 (br s, 1H, amide-N*H*), 8.86-8.77 (br s, 1H, imide-N*H*), 8.80 (d, 1H, H-7′, $J_{7',6'}$ = 8.7 Hz), 8.07 (dd, 1H, H-12, $J_{12,11}$ = 9.6, $J_{12,9}$ = 1.4 Hz), 7.92 (s, 1H, triazole-C*H*), 7.69 (dd, 1H, H-6′, $J_{6',5'}$ = $J_{6',7'}$ = 8.2 Hz), 7.57-7.52 (m, 3H, H-9, H-14, H-5′), 7.48 (dd, 1H, H-11, $J_{11,12}$ = 9.2, $J_{11,9}$ = 1.4 Hz), 5.74 (d, 1H, H-22, J_{gem} = 16.5 Hz), 5.40 (s, 2H, H-5), 5.29 (d, 1H, H-22, J_{gem} = 16.5 Hz), 5.21 (s, 2H, triazole-C*H*$_2$-O), 4.98-4.92 (m, 1H, H-11′), 4.57 (t, 2H, O-C*H*$_2$-CON, *J* = 5.0 Hz), 4.18 (d, 1H, C*H*$_2$, J_{gem} = 16.0 Hz), 4.13 (d, 1H, C*H*$_2$, J_{gem} = 16.0 Hz), 3.92-3.85 (m, 3H, C*H*$_2$, OH), 3.79-3.73 (m, 4H, C*H*$_2$×2), 3.68-3.54 (m, 8H, C*H*$_2$×4), 3.13 (q, 2H, H-23, $J_{23,24}$ = 7.8 Hz), 2.92-2.74 (m, 3H, H-12′, H-13′), 2.22-2.15 (m, 1H, H-12′), 1.95-1.82 (m, 2H, H-19), 1.36 (t, 3H, H-24, $J_{24,23}$ = 7.8 Hz), 1.03 (t, 3H, H-18, $J_{18,19}$ = 7.5 Hz); LCMS-ESI 963, calcd. 963.4 [$(M+H)^+$].

SN38-(PEG)$_5$-Pom-α

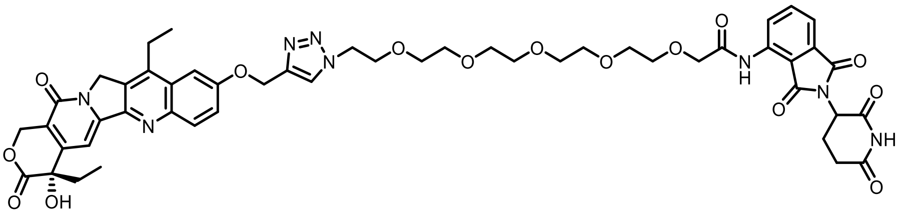

According to general procedure, alkyne **3** (20 mg, 45.3 μmol) and pomalidomide-PEG5-azide (50 mg) were used to afford SN38-(PEG)$_5$-Pom-α (9.4 mg, 21%) as a pale yellow solid.

^{1}H NMR (400 MHz, CDCl$_3$) δ 10.45 (br s, 1H, amide-N*H*), 9.00-8.90 (br s, 1H, imide-N*H*), 8.81 (d, 1H, H-7′, $J_{7',6'}$ = 8.7 Hz), 8.11 (d, 1H, H-12, $J_{12,11}$ = 9.6 Hz), 7.92 (s, 1H, triazole-C*H*), 7.70 (dd, 1H, H-6′, $J_{6',5'}$ = $J_{6',7'}$ = 8.0 Hz), 7.59-7.47 (m, 4H, H-9, H-11, H-14, H-5′), 5.74 (d, 1H, H-22, J_{gem} = 16.5 Hz), 5.41 (s, 2H, H-5), 5.30 (d, 1H, H-22, J_{gem} = 16.5 Hz), 5.22 (s, 2H, triazole-CH_2-O), 5.00-4.95 (m, 1H, H-11′), 4.56 (t, 2H, O-CH_2-CON, *J* = 5.0 Hz), 4.18 (d, 1H, CH_2, J_{gem} = 16.5 Hz), 4.14 (d, 1H, CH_2, J_{gem} = 16.5 Hz), 3.90-3.56 (m, 19H, CH_2×9, OH), 3.14 (q, 2H, H-23, $J_{23,24}$ = 7.8 Hz), 2.91-2.75 (m, 3H, H-12′, H-13′), 2.21-2.14 (m, 1H, H-12′), 1.95-1.81 (m, 2H, H-19), 1.37 (t, 3H, H-24, $J_{24,23}$ = 7.8 Hz), 1.03 (t, 3H, H-18, $J_{18,19}$ = 7.6 Hz); LCMS-ESI 1007, calcd.1007.4 [(M+H)$^+$].

SN38-(PEG)$_6$-Pom-α

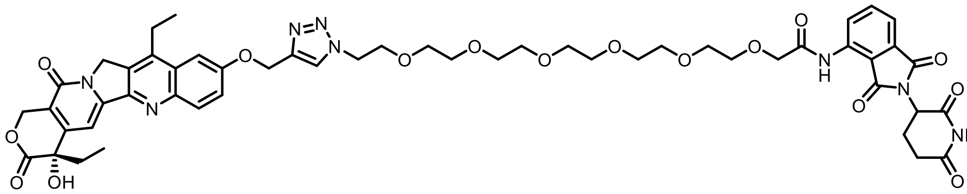

According to general procedure, alkyne **3** (20 mg, 45.3 μmol) and pomalidomide-PEG6-azide (50 mg) were used to afford SN38-(PEG)$_6$-Pom-α (5.3 mg, 11%) as a pale yellow solid.

^{1}H NMR (400 MHz, CDCl$_3$) δ 10.46 (br s, 1H, amide-N*H*), 9.06-8.98 (br s, 1H, imide-N*H*), 8.82 (d, 1H, H-7′, $J_{7',6'}$ = 8.4 Hz), 8.12 (d, 1H, H-12, $J_{12,11}$ = 9.6 Hz), 7.94 (s, 1H, triazole-C*H*), 7.70 (dd, 1H, H-6′, $J_{6',5'}$ = $J_{6',7'}$ = 8.0 Hz), 7.60-7.47 (m, 4H, H-9, H-11, H-14, H-5′), 5.74 (d, 1H, H-22, J_{gem} = 16.4 Hz), 5.40 (s, 2H, H-5), 5.30 (d, 1H, H-22, J_{gem} = 16.4 Hz), 5.22 (s, 2H, triazole-CH_2-O), 4.98-4.93 (m, 1H, H-11′), 4.57 (t, 2H, O-CH_2-CON, *J* = 5.0 Hz), 4.18 (d, 1H, CH_2, J_{gem} = 16.0 Hz), 4.14 (d, 1H, CH_2, J_{gem} = 16.0 Hz), 3.89-3.86 (m, 3H, CH_2, OH), 3.79-3.77 (m, 4H, CH_2×2), 3.75-3.55 (m, 18H, CH_2×9), 3.14 (q, 2H, H-23, $J_{23,24}$ = 778 Hz), 2.91-2.74 (m, 3H, H-12′, H-13′), 2.19-2.14 (m, 1H, H-12′), 1.94-1.82 (m, 2H, H-19), 1.37 (t, 3H, H-24, $J_{24,23}$ = 7.7 Hz), 1.03 (t, 3H, H-18, $J_{18,19}$ = 7.4 Hz); LCMS-ESI 1052, calcd. 1051.4 [(M+H)$^+$].

SN38-(PEG)$_1$-Pom-β

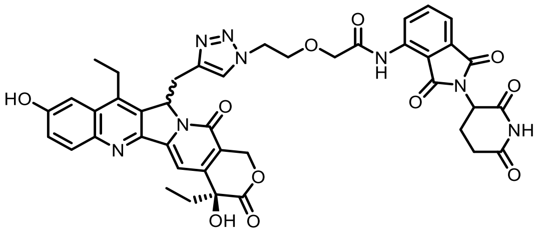

According to general procedure, alkyne **6** (5.0 mg, 11.6 μmol) and pomalidomide-PEG1-azide (6.0 mg) were used to afford SN38-(PEG)$_1$-Pom-β (5.8 mg, 60%) as a pale yellow solid.

^{1}H NMR (400 MHz, 10% CD$_3$OD/CDCl$_3$, The total amount of a mixture of stereoisomers is defined as 1, and the number of protons is specified.) δ 10.19-10.9.89 (m, 1H, amide-N*H*), 8.82-8.79 (m, 1H,

H-7′), 7.95-7.58 (m, 3H, H-12, H-5′, H-6′), 7.37-6.95 (m, 4H, H-9, H-11, H-14, triazole-C*H*), 6.11-6.04 (m, 1H, H-5), 5.83-5.65 (m, 1H, H-22), 5.42-5.21 (m, 1H, H-22), 5.08-4.90 (m, 1H, H-11′), 4.58-4.26 (m, 3H, triazole-CH_2, O-CH_2-CON), 4.10-3.86 (m, 2H, CH_2), 3.79-3.47 (m, 3H, triazole-CH_2, CH_2), 3.26-3.11 (m, 2H, H-23), 2.92-2.69 (m, 3H, H-12′, H-13′), 2.22-2.09 (m, 1H, H-12′), 1.93-1.82 (m, 2H, H-19), 1.46-1.35 (m, 3H, H-24), 1.01-0.92 (m, 3H, H-18); LCMS-ESI 831, calcd. 831.3 $[(M+H)^+]$.

SN38-(PEG)$_2$-Pom-β

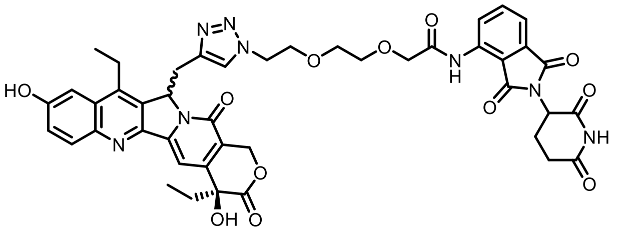

According to general procedure, alkyne **6** (5.0 mg, 11.6 μmol) and pomalidomide-PEG2-azide (6.0 mg) were used to afford SN38-(PEG)$_2$-Pom-β (1.8 mg, 18%) as a pale yellow solid.

^{1}H NMR (400 MHz, $CDCl_3$, The total amount of a mixture of stereoisomers is defined as 1, and the number of protons is specified.) δ 10.39-10.35 (m, 1H, amide-N*H*), 8.88-8.83 (m, 1H, H-7′), 7.78-7.57 (m, 3H, H-12, H-5′, H-6′), 7.30 (s, 1H, triazole-C*H*), 7.21-6.88 (m, 3H, H-9, H-11, H-14), 6.10-6.04 (m, 1H, H-5), 5.82-5.68 (m, 1H, H-22), 5.37, 5.36, 5.27 (each d, 1H, H-22, J_{gem} = 16.4 Hz), 4.99-4.90 (m, 1H, H-11′), 4.49-4.40 (m, 1H, triazole-CH_2), 4.38-4.07 (m, 4H, CH_2, O-CH_2-CON), 3.77-3.48 (m, 7H, triazole-CH_2, CH_2×3), 3.41-3.10 (m, 2H, H-23), 2.90-2.68 (m, 3H, H-12′, H-13′), 2.17-2.08 (m, 1H, H-12′), 1.92-1.80 (m, 2H, H-19), 1.46-1.38 (m, 3H, H-24), 1.01-0.92 (m, 3H, H-18); LCMS-ESI 875, calcd. 875.3 $[(M+H)^+]$.

SN38-(PEG)$_3$-Pom-β

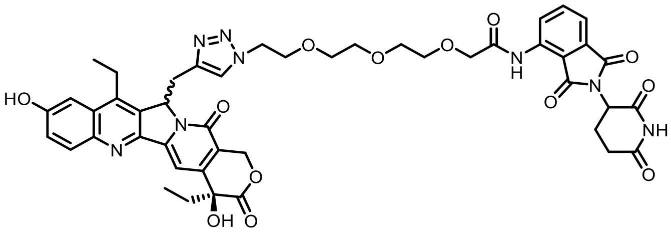

According to general procedure, alkyne **6** (5.0 mg, 11.6 μmol) and pomalidomide-PEG3-azide (6.0 mg) were used to afford SN38-(PEG)$_3$-Pom-β (3.7 mg, 35%) as a pale yellow solid.

^{1}H NMR (400 MHz, $CDCl_3$, The total amount of a mixture of stereoisomers is defined as 1, and the number of protons is specified.) δ 10.49-10.43 (m, 1H, amide-N*H*), 9.40-9.26 (m, 1H, imide-N*H*), 8.88-8.82 (m, 1H, H-7′), 7.76-7.57 (m, 3H, H-12, H-5′, H-6′), 7.28 (s, 1H, triazole-C*H*), 7.17-6.82 (m, 3H, H-9, H-11, H-14), 6.12-6.04 (m, 1H, H-5), 5.78, 5.73, 5.47, 5.27 (each d, 2H, H-22, J_{gem} = 16.4

Hz), 5.03-4.96 (m, 1H, H-11′), 4.50-4.40 (m, 1H, triazole-C*H*$_2$), 4.34-4.07 (m, 4H, C*H*$_2$, O-C*H*$_2$-CON), 3.85-3.31 (m, 11H, triazole-C*H*$_2$, C*H*$_2$×5), 3.31-3.10 (m, 2H, H-23), 2.93-2.74 (m, 3H, H-12′, H-13′), 2.22-2.14 (m, 1H, H-12′), 1.92-1.79 (m, 2H, H-19), 1.47-1.36 (m, 3H, H-24), 1.01-0.93 (m, 3H, H-18); LCMS-ESI 919, calcd. 919.3 [$(M+H)^+$].

SN38-(PEG)$_4$-Pom-β

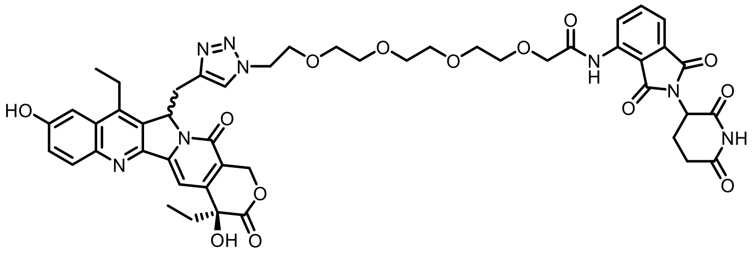

According to general procedure, alkyne **6** (5.0 mg, 11.6 μmol) and pomalidomide-PEG4-azide (6.0 mg) were used to afford SN38-(PEG)$_4$-Pom-β (5.5 mg, 49%) as a pale yellow solid.

^{1}H NMR (400 MHz, CDCl$_3$, The total amount of a mixture of stereoisomers is defined as 1, and the number of protons is specified.) δ 10.51-10.49 (m, 1H, amide-N*H*), 9.23-8.72 (m, 2H, imide-N*H*, OH), 8.85-8.82 (m, 1H, H-7′), 7.75-7.70 (m, 1.5H, H-12, H-6′), 7.63 (dd, 0.5H, H-12, $J_{12,11}$ = 9.2, $J_{12,9}$ = 1.4 Hz), 7.60-7.57 (m, 1H, H-5′), 7.28 (s, 1H, triazole-C*H*), 7.20 (t, 0.5H, H-9, $J_{9,11}$ = $J_{9,12}$ = 3.0 Hz), 7.17, 7.15, 6.99, 6.98 (each s, 1H, H-14), 7.12 (t, 0.5H, H-9, $J_{9,11}$ = $J_{9,12}$ = 2.3 Hz), 7.06-7.02 (m, 0.5H, H-11), 6.91-6.88 (m, 0.5H, H-11), 6.13-6.06 (m, 1H, H-5), 5.81-5.70 (m, 1H, H-22), 5.37 (d, 0.5H, H-22, J_{gem} = 16.0 Hz), 5.28 (d, 0.25H, H-22, J_{gem} = 16.0 Hz), 5.27 (d, 0.25H, H-22, J_{gem} = 16.0 Hz), 5.01-4.95 (m, 1H, H-11′), 4.48-4.42 (m, 1H, triazole-C*H*$_2$), 4.34-4.10 (m, 4H, C*H*$_2$, O-C*H*$_2$-CON), 3.84-3.33 (m, 15H, triazole-C*H*$_2$, C*H*$_2$×7), 3.30-3.12 (m, 2H, H-23), 2.94-2.73 (m, 3H, H-12′, H-13′), 2.20-2.14 (m, 1H, H-12′), 1.93-1.80 (m, 2H, H-19), 1.45-1.38 (m, 3H, H-24), 1.01-0.95 (m, 3H, H-18); LCMS-ESI 963, calcd. 963.4[$(M+H)^+$].

SN38-(PEG)$_5$-Pom-β

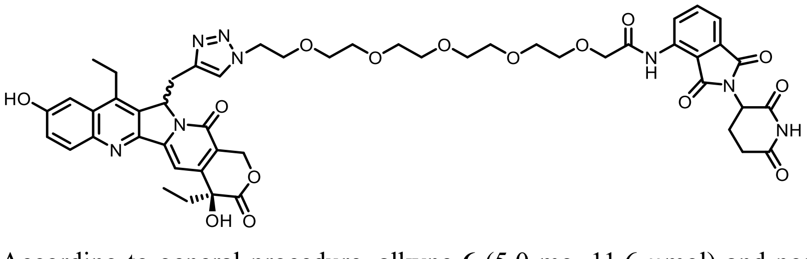

According to general procedure, alkyne **6** (5.0 mg, 11.6 μmol) and pomalidomide-PEG5-azide (4.7 mg) were used to afford SN38-(PEG)$_5$-Pom-β (2.2 mg, 19%) as a pale yellow solid.

^{1}H NMR (400 MHz, CDCl$_3$, The total amount of a mixture of stereoisomers is defined as 1, and the number of protons is specified.) δ 10.45, 10.33, 10.28 (each s, 1H, amide-N*H*), 8.83-8.72 (m, 1H, H-

7′), 7.85, 7.83 (each d, 0.5H, H-12, $J_{12,11}$ = 9.0 Hz), 7.72-7.61 (m, 1.5H, H-12, H-6′), 7.56-7.47 (m, 1H, H-5′), 7.32 (s, 0.5H, H-9), 7.28-7.24 (m, 0.75H, H-11), 7.22, 7.21 (each s, 1H, triazole-C*H*), 7.04-7.00 (m, 0.75H, H-11, H-14), 6.72 (s, 0.5H, H-14), 6.13-6.03 (m, 1H, H-5), 5.80-5.73 (m, 1H, H-22), 5.38 (d, 0.5H, H-22, J_{gem} = 16.2 Hz), 5.27 (d, 0.25H, H-22, J_{gem} = 16.5 Hz), 5.27 (d, 0.25H, H-22, J_{gem} = 16.2 Hz), 5.01-4.93 (m, 1H, H-11′), 4.49-4.44 (m, 1H, triazole-CH_2), 4.30-4.05 (m, 4H, CH_2, O-CH_2-CON), 3.86-3.14 (m, 21H, H-23, triazole-CH_2, CH_2×9), 2.97-2.74 (m, 3H, H-12′, H-13′), 2.23-2.15 (m, 1H, H-12′), 1.95-1.81 (m, 2H, H-19), 1.48-1.40 (m, 3H, H-24), 1.02-0.97 (m, 3H, H-18); LCMS-ESI 1007, calcd. 1007.4 [$(M+H)^+$].

SN38-(PEG)$_6$-Pom-β

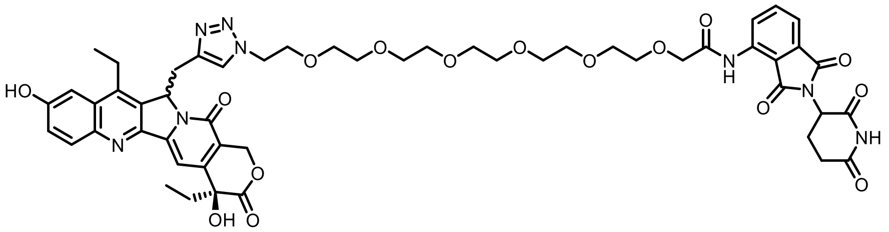

According to general procedure, alkyne **6** (5.0 mg, 11.6 μmol) and pomalidomide-PEG6-azide (6.0 mg) were used to afford SN38-(PEG)$_6$-Pom-β (2.5 mg, 21%) as a pale yellow solid.

^{1}H NMR (400 MHz, $CDCl_3$, The total amount of a mixture of stereoisomers is defined as 1, and the number of protons is specified.) δ 10.44, 10.30, 10.26 (each s, 1H, amide-N*H*), 8.83-8.74 (m, 1H, H-7′), 7.89, 7.88 (each d, 0.5H, H-12, $J_{12,11}$ = 9.2 Hz), 7.78-7.63 (m, 1.5H, H-12, H-6′), 7.57-7.46 (m, 1H, H-5′), 7.48-7.28 (m, 1.5H, H-9, H-11), 7.21, 7.20 (each s, 1H, triazole-C*H*), 7.13-7.06 (m, 0.5H, H-11), 6.93, 6.91, 6.66 (s, 1H, H-14), 6.13-6.01 (m, 1H, H-5), 5.78, 5.74, 5.37, 5.27 (each d, 1H, H-22, J_{gem} = 16.4 Hz), 5.01-4.92 (m, 1H, H-11′), 4.53-4.46 (m, 1H, triazole-CH_2), 4.27-4.04 (m, 4H, CH_2, O-CH_2-CON), 3.88-3.13 (m, 25H, H-23, triazole-CH_2, CH_2×11), 2.94-2.74 (m, 3H, H-12′, H-13′), 2.23-2.14 (m, 1H, H-12′), 1.94-1.80 (m, 2H, H-19), 1.48-1.42 (m, 3H, H-24), 1.02-0.95 (m, 3H, H-18); LCMS-ESI 1051, calcd. 1051.4 [$(M+H)^+$].

^{1}H NMR spectra of compounds

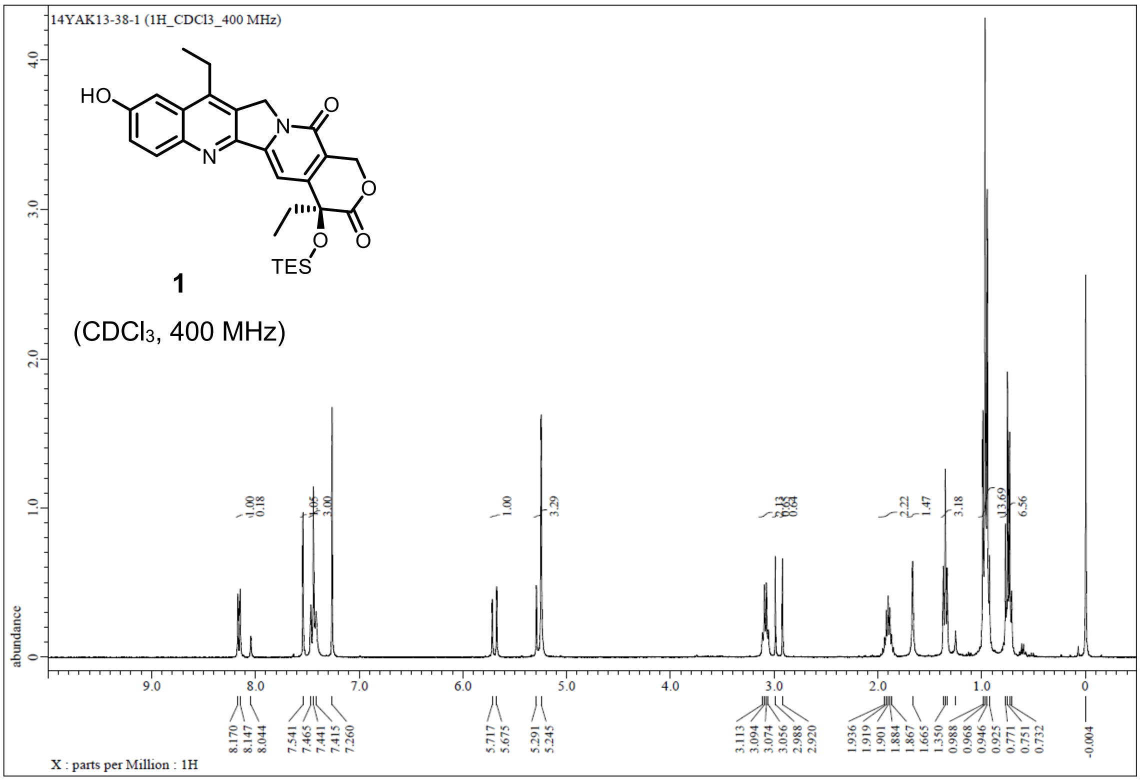

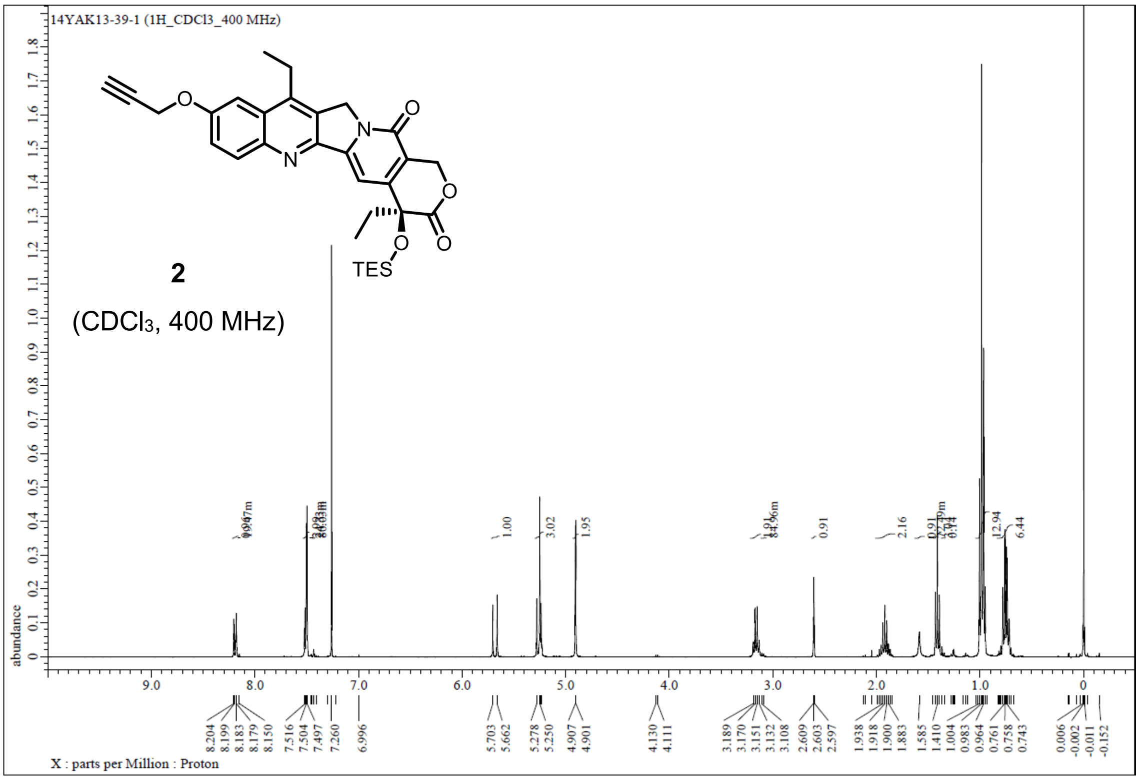

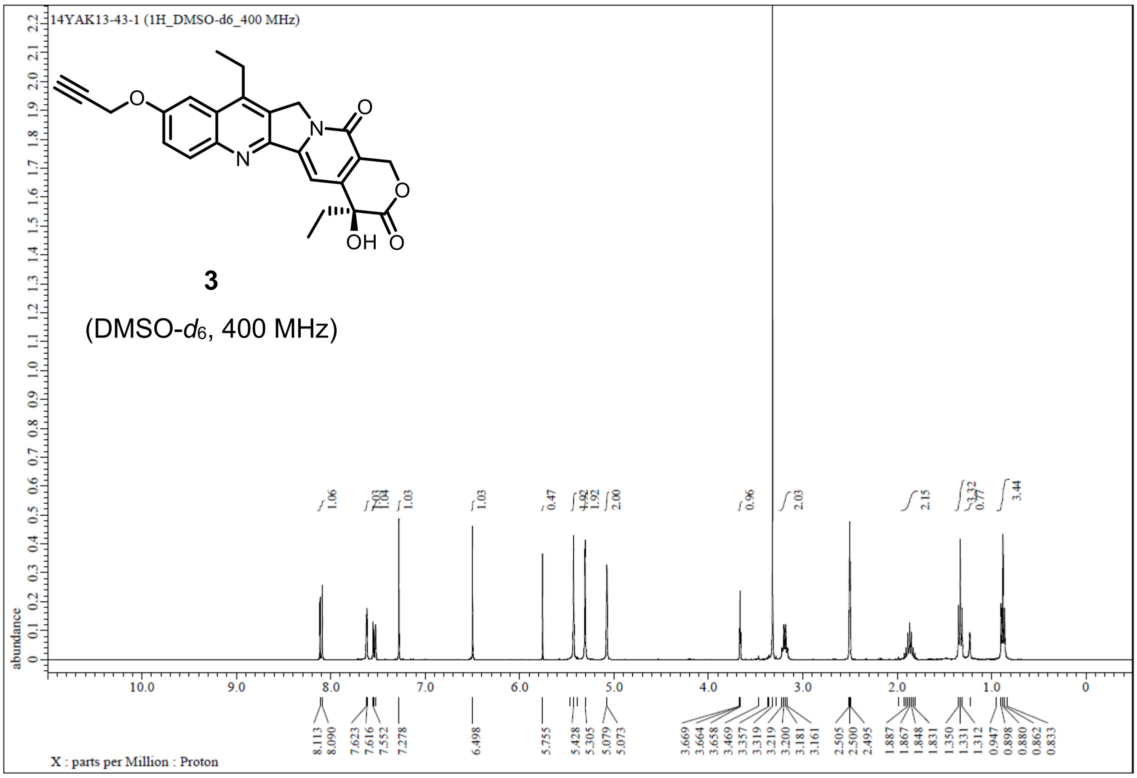
14YAK13-43-1 (1H_DMSO-d6_400 MHz)
3
(DMSO-d_6, 400 MHz)
abundance
X : parts per Million : Proton

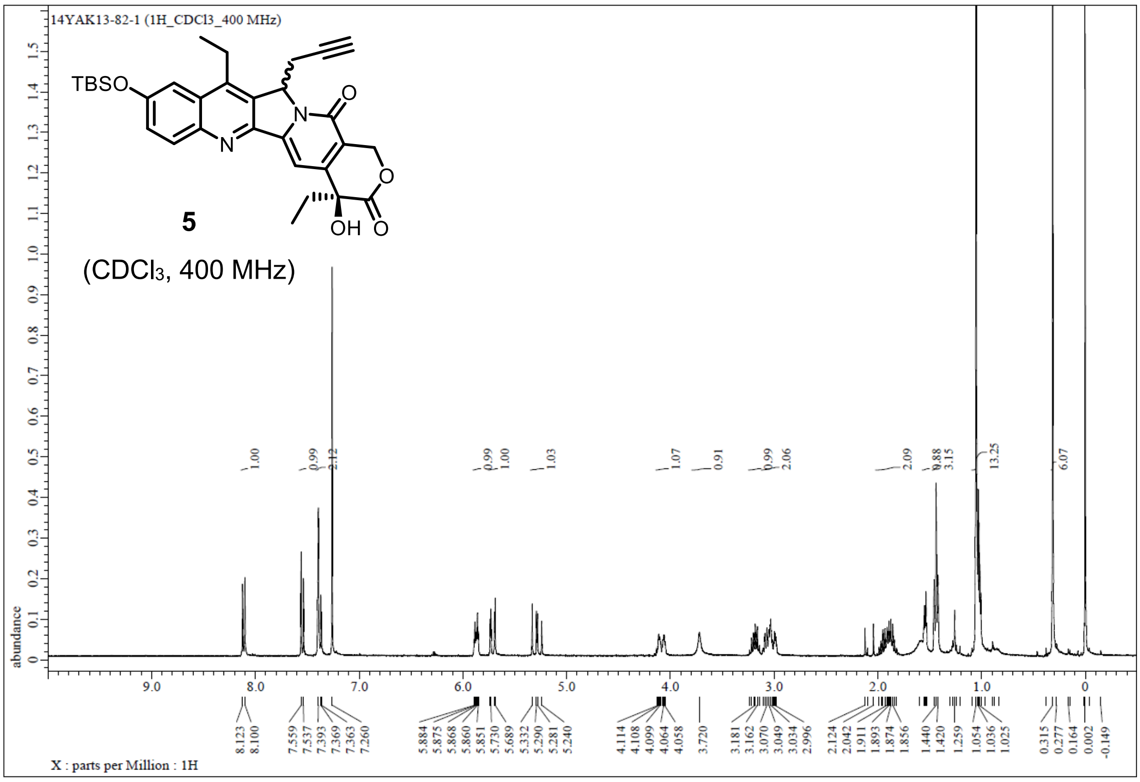
14YAK13-82-1 (1H_CDCl3_400 MHz)
TBSO
5
($CDCl_3$, 400 MHz)
abundance
X : parts per Million : 1H

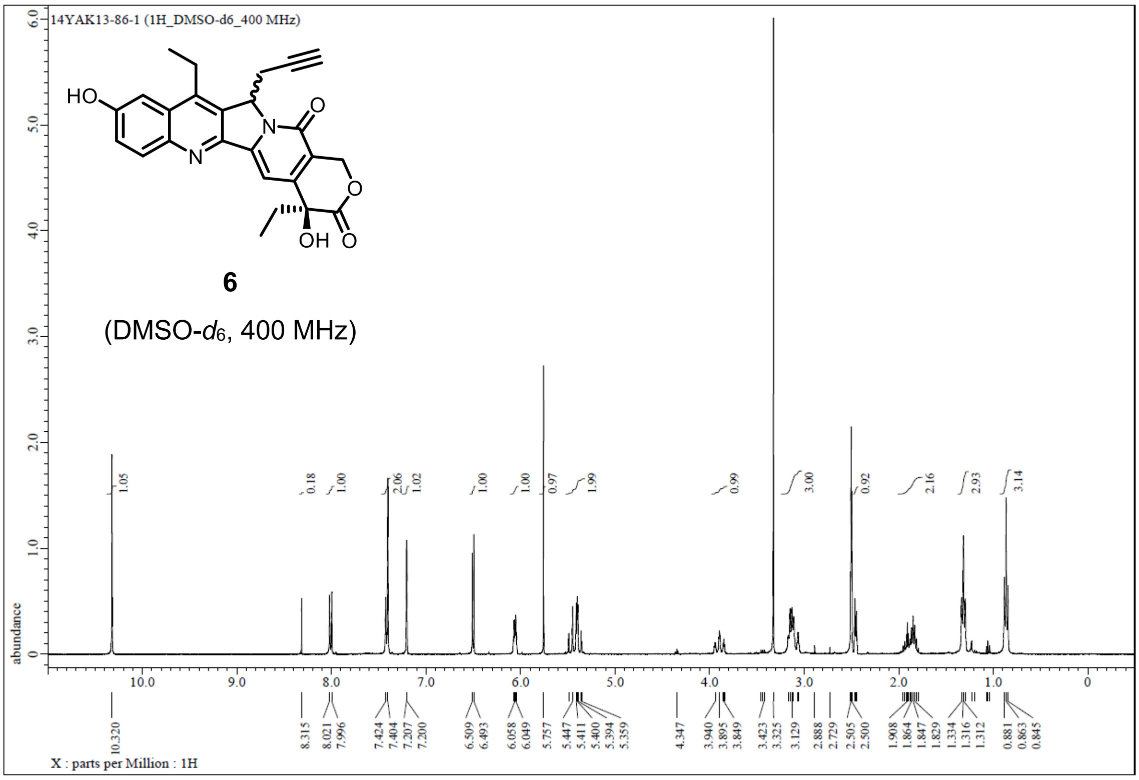
14YAK13-86-1 (1H_DMSO-d6_400 MHz)
6
(DMSO-d6, 400 MHz)
abundance
X : parts per Million : 1H

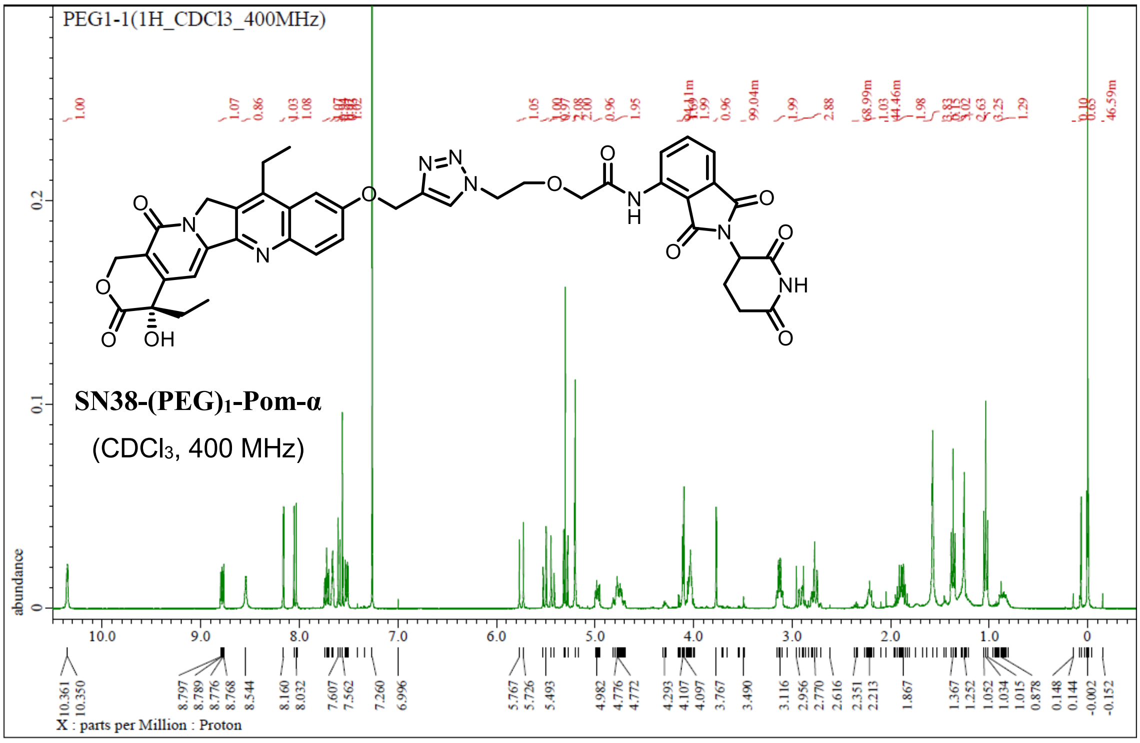
PEG1-1(1H_CDCl3_400MHz)
SN38-(PEG)1-Pom-α
(CDCl3, 400 MHz)
abundance
X : parts per Million : Proton

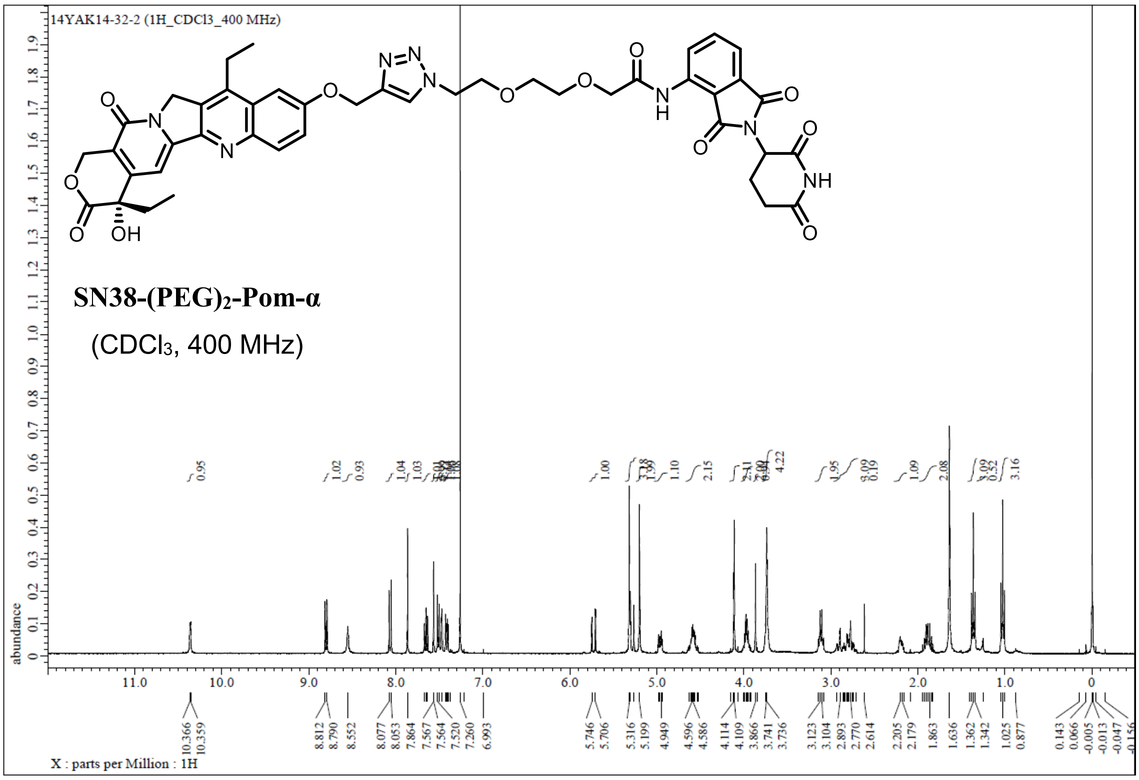
14YAK14-32-2 (1H_CDCl3_400 MHz)
SN38-(PEG)2-Pom-α
(CDCl3, 400 MHz)
abundance
X : parts per Million : 1H

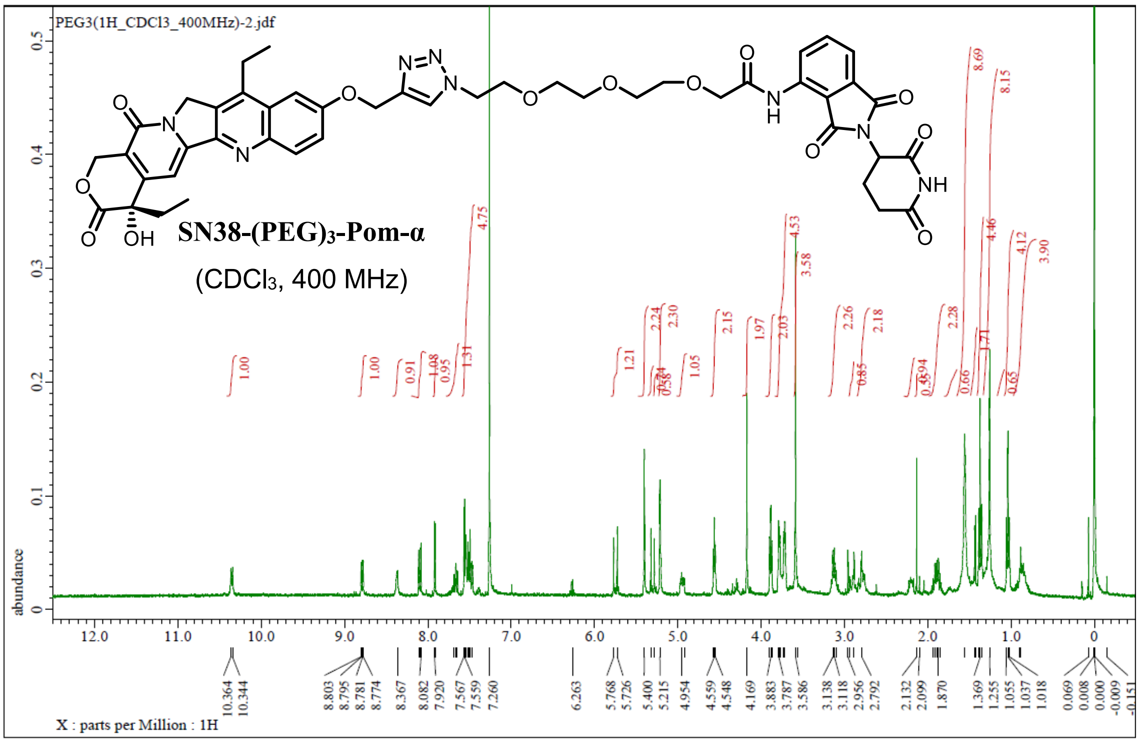
PEG3(1H_CDCl3_400MHz)-2.jdf
SN38-(PEG)3-Pom-α
(CDCl3, 400 MHz)
abundance
X : parts per Million : 1H

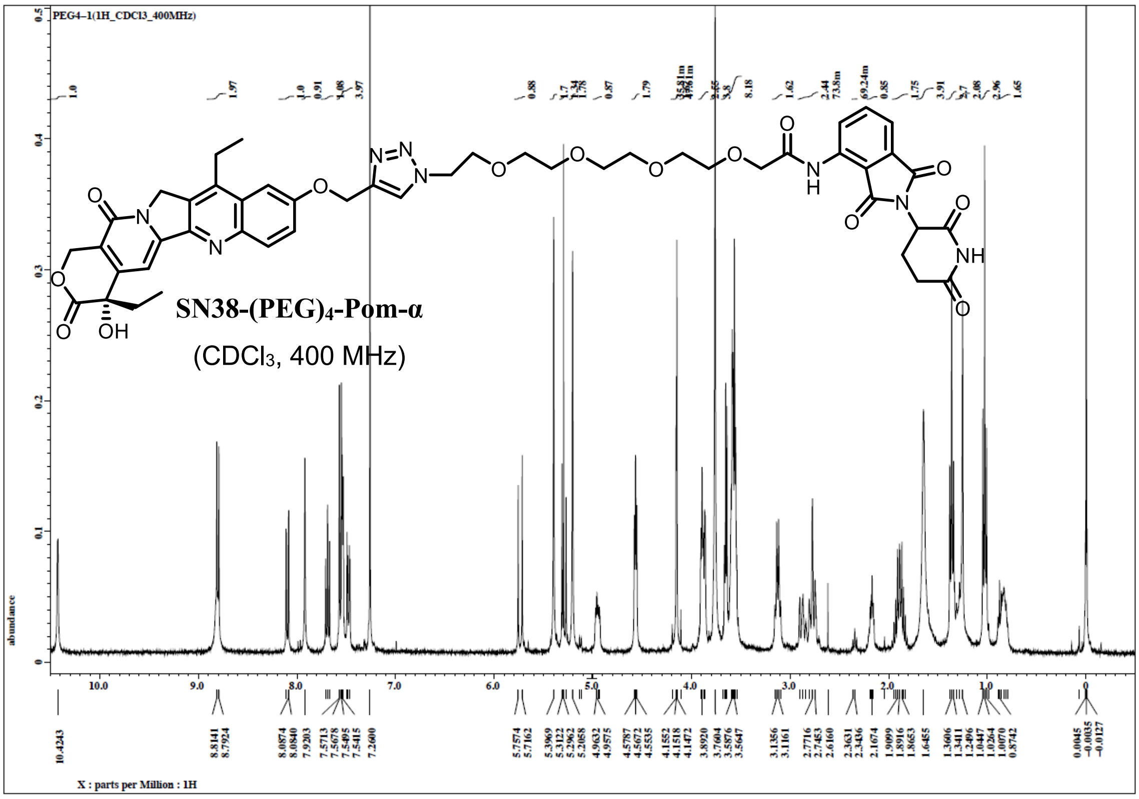
PEG4–1(1H_CDCl3_400MHz)
SN38-(PEG)4-Pom-α
(CDCl3, 400 MHz)
abundance
X : parts per Million : 1H

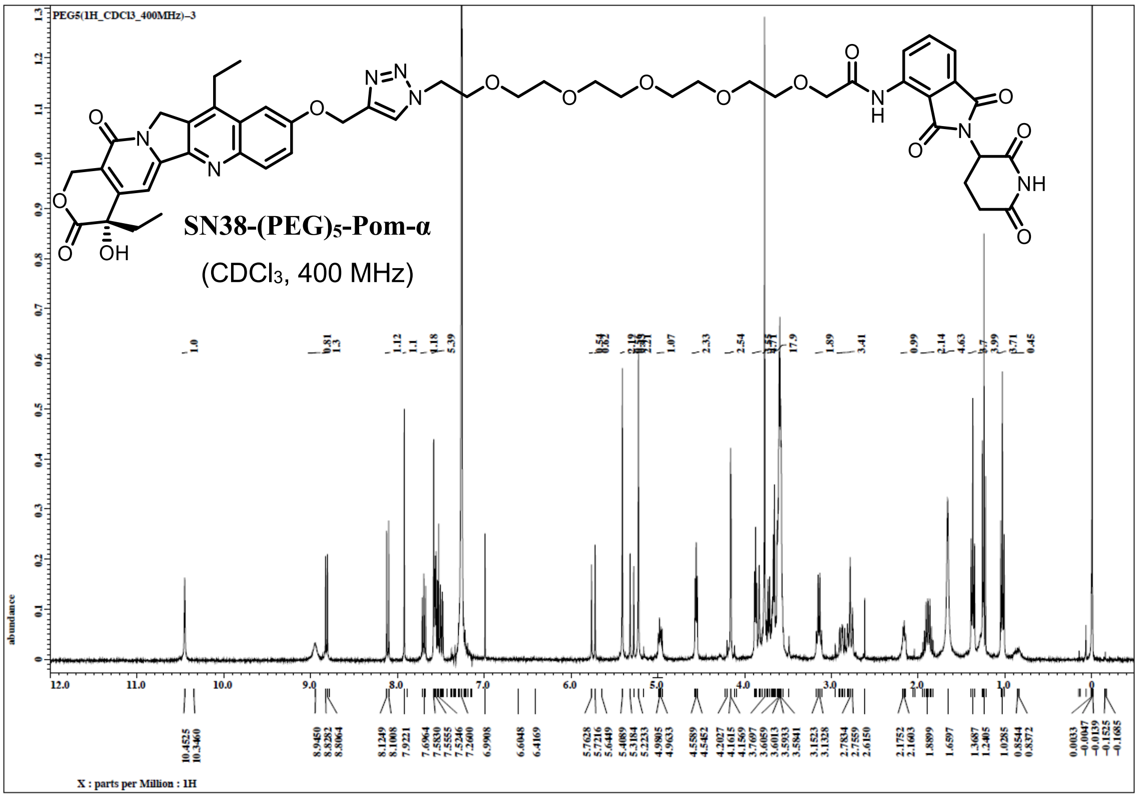
PEG5(1H_CDCl3_400MHz)–3
SN38-(PEG)5-Pom-α
(CDCl3, 400 MHz)
abundance
X : parts per Million : 1H

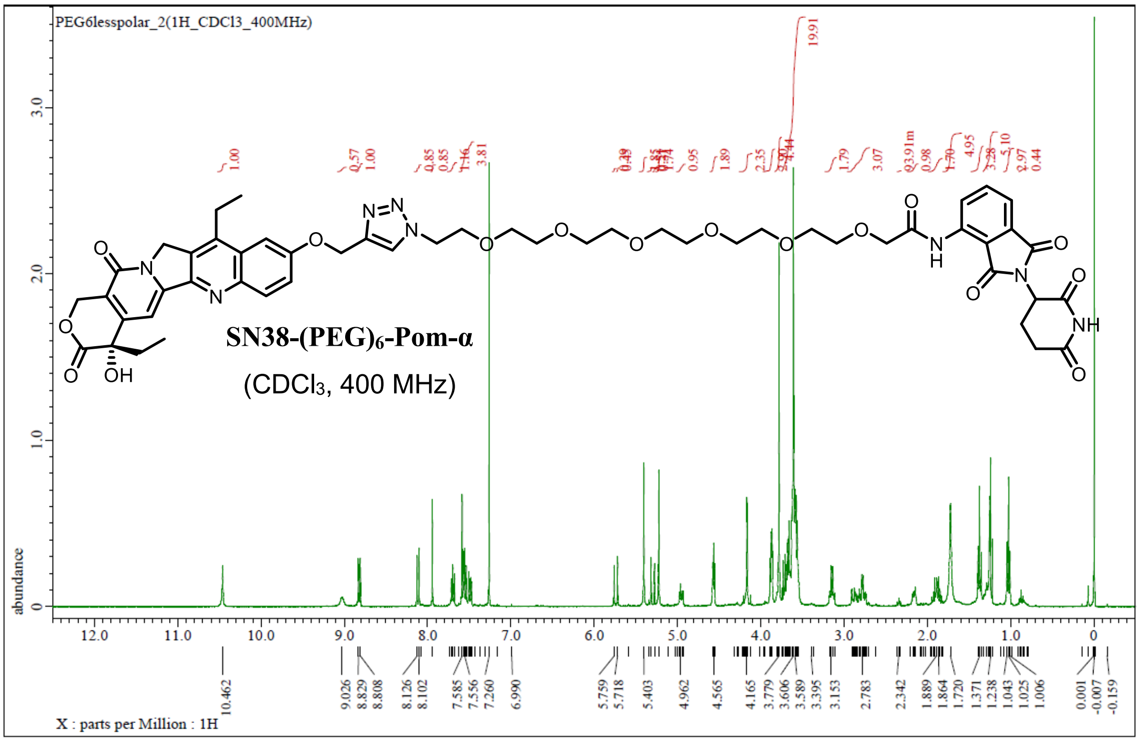
PEG6lesspolar_2(1H_CDCl3_400MHz)
SN38-(PEG)6-Pom-α
(CDCl3, 400 MHz)
abundance
X : parts per Million : 1H

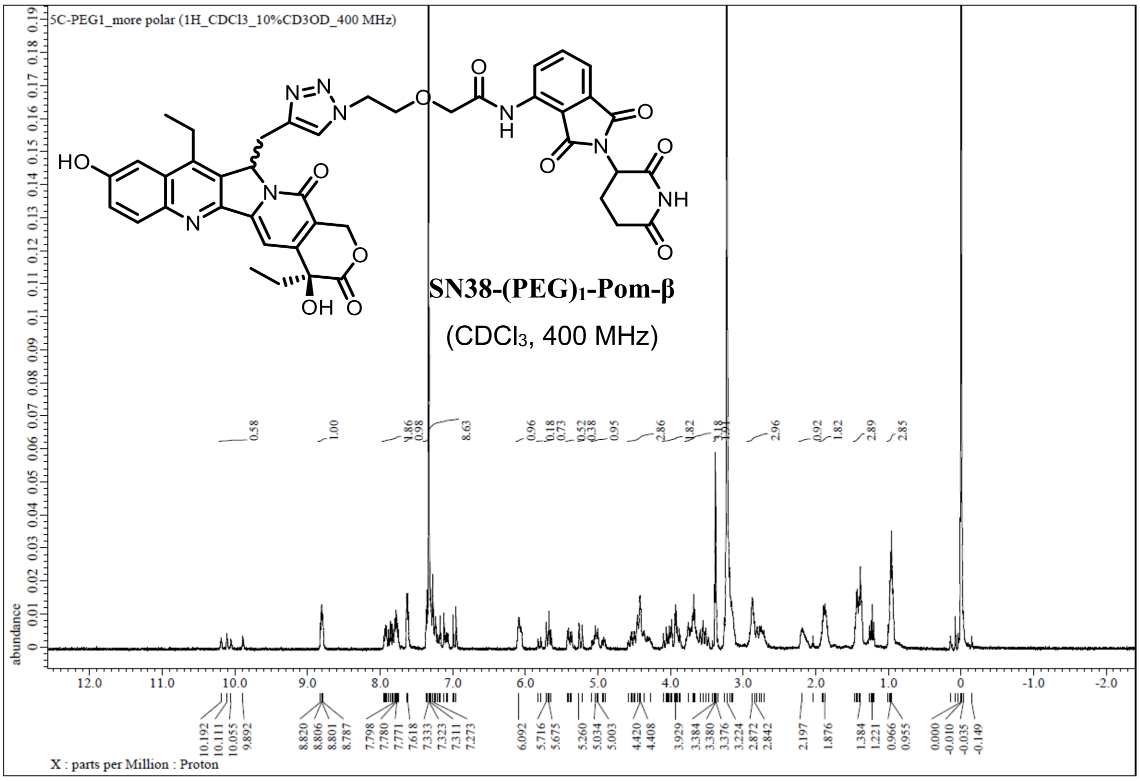
5C-PEG1_more polar (1H_CDCl3_10%CD3OD_400 MHz)
SN38-(PEG)1-Pom-β
(CDCl3, 400 MHz)
abundance
X : parts per Million : Proton

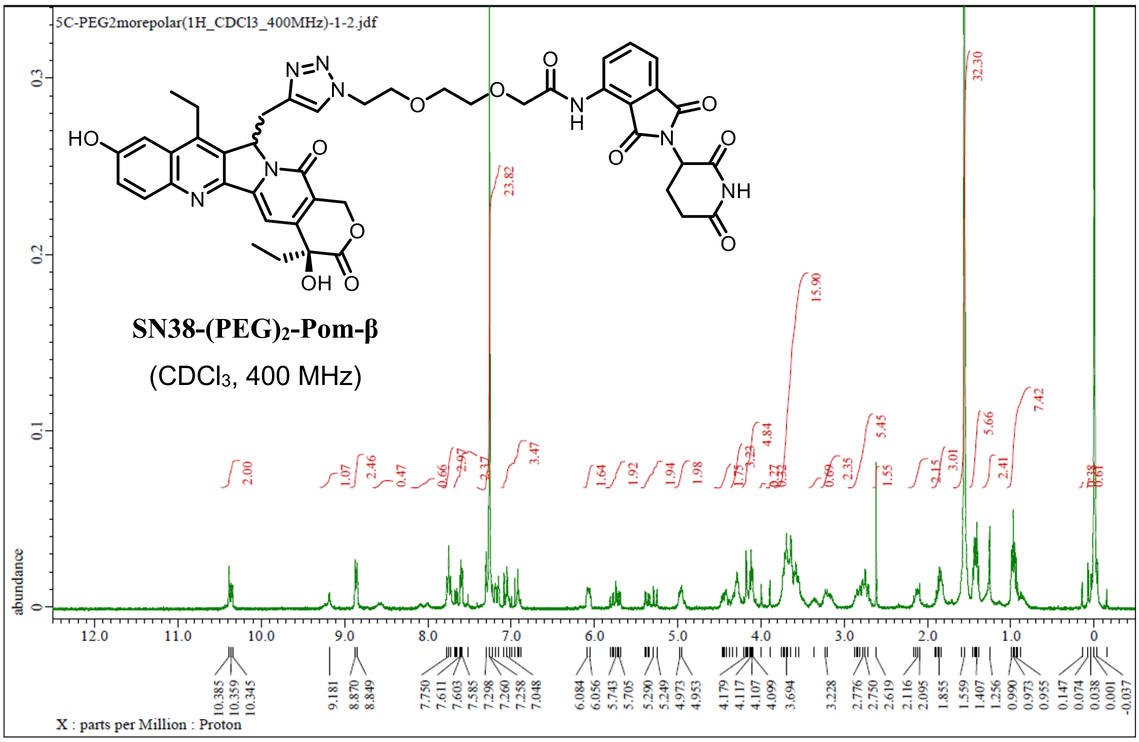
5C-PEG2morepolar(1H_CDCl3_400MHz)-1-2.jdf
SN38-(PEG)2-Pom-β
(CDCl3, 400 MHz)
abundance
X : parts per Million : Proton

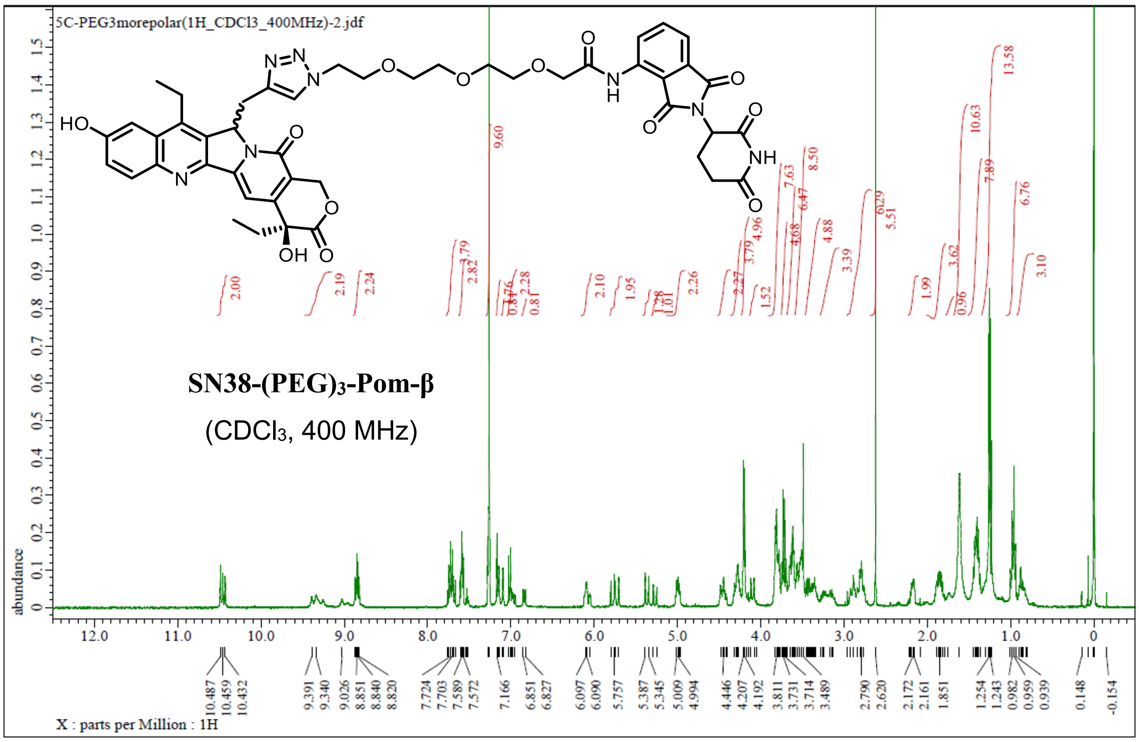
5C-PEG3morepolar(1H_CDCl3_400MHz)-2.jdf
SN38-(PEG)3-Pom-β
(CDCl3, 400 MHz)
abundance
X : parts per Million : 1H

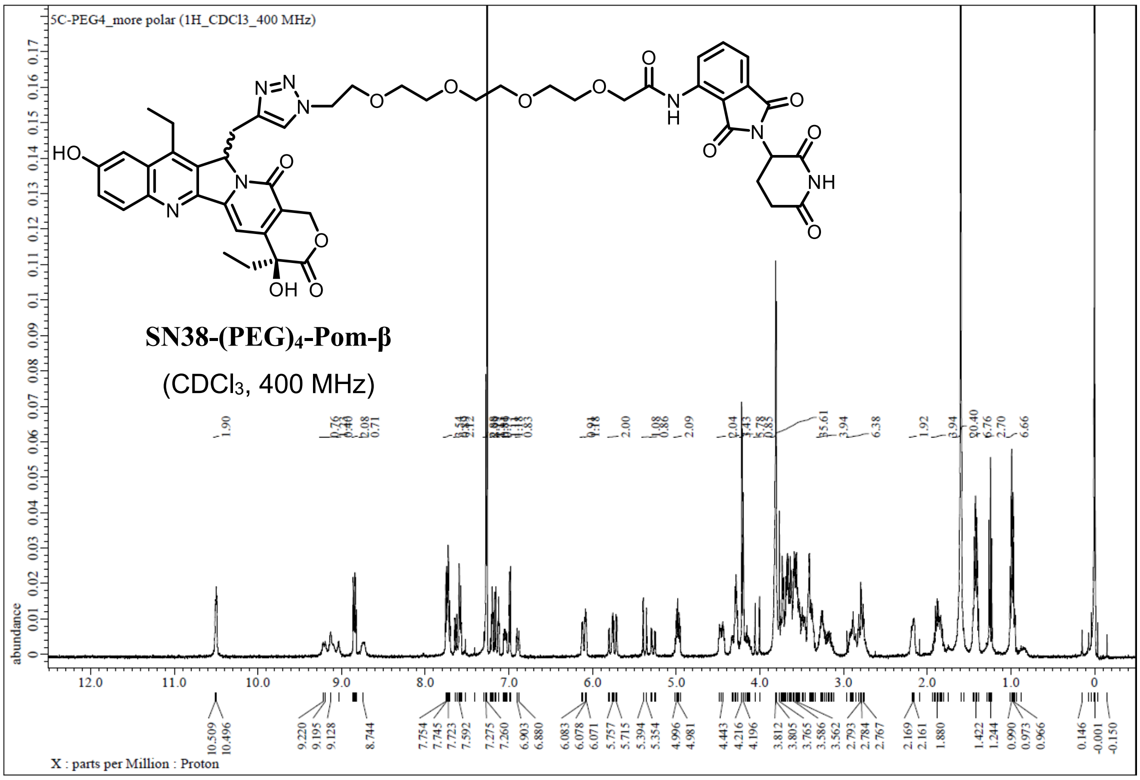
5C-PEG4_more polar (1H_CDCl3_400 MHz)
SN38-(PEG)4-Pom-β
(CDCl3, 400 MHz)
abundance
X : parts per Million : Proton

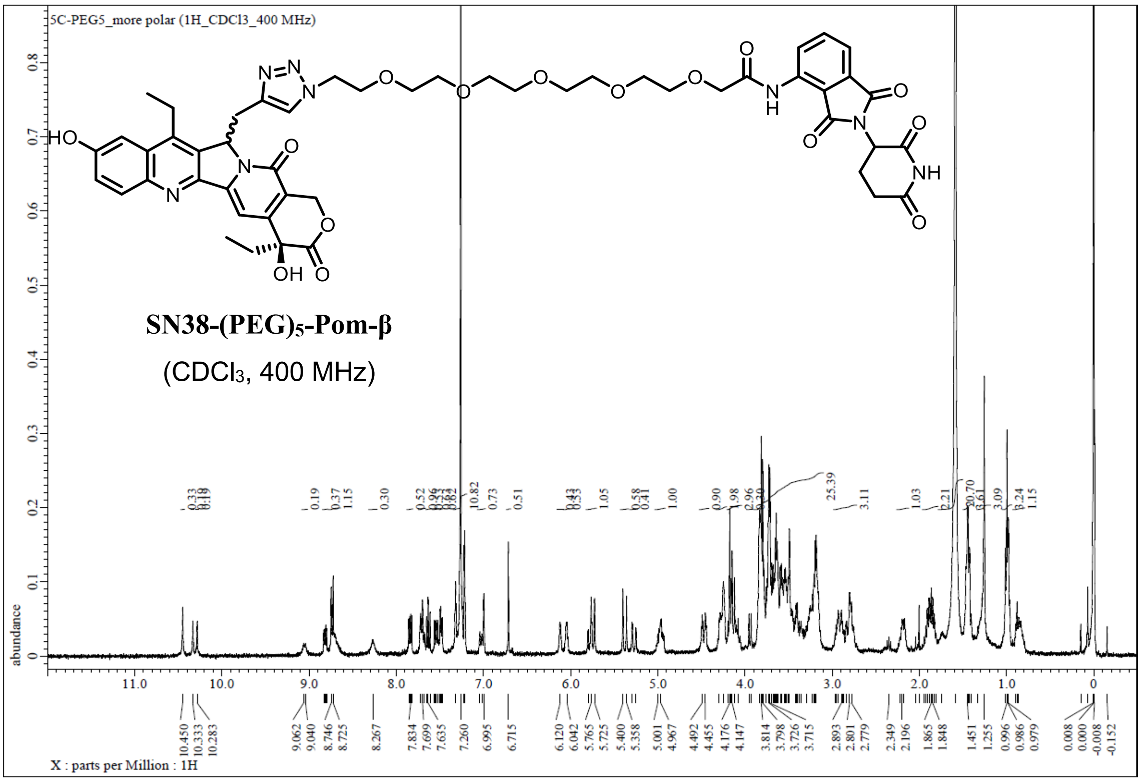
5C-PEG5_more polar (1H_CDCl3_400 MHz)
SN38-(PEG)5-Pom-β
(CDCl3, 400 MHz)
abundance
X : parts per Million : 1H

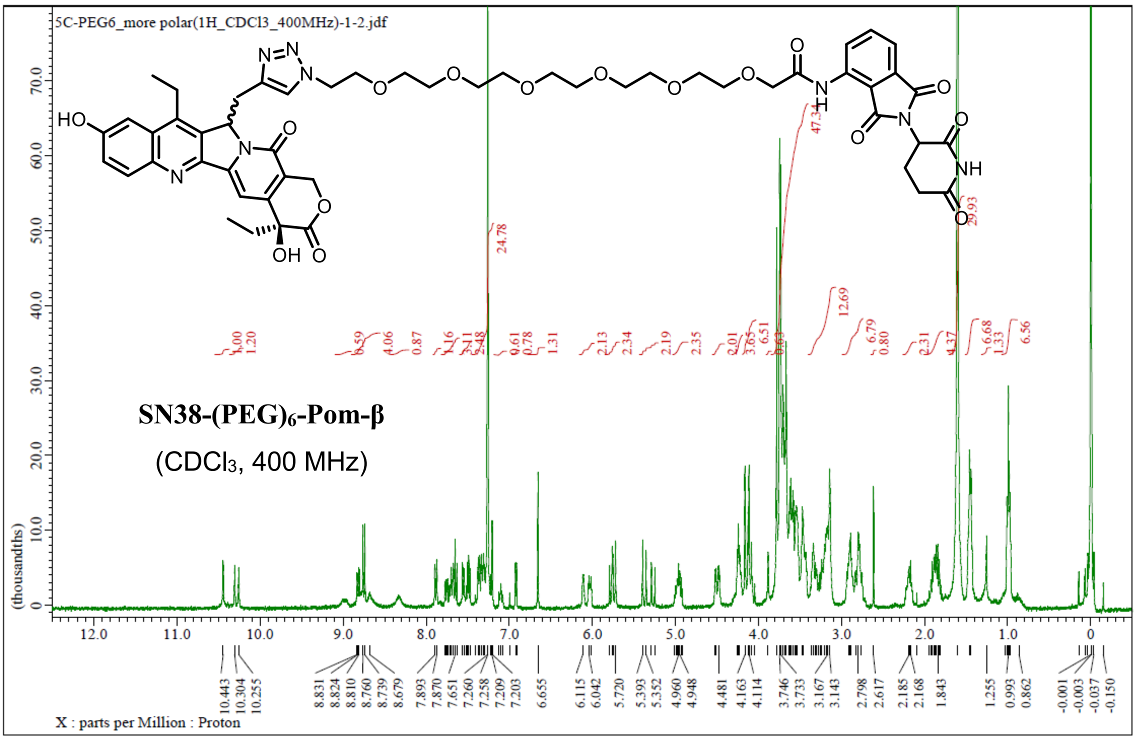

5C-PEG6_more polar(1H_CDCl3_400MHz)-1-2.jdf
SN38-(PEG)$_6$-Pom-β
(CDCl$_3$, 400 MHz)
(thousandths)
X : parts per Million : Proton
10.443
10.304
10.255
8.831
8.824
8.810
8.760
8.739
8.679
7.893
7.870
7.651
7.260
7.258
7.209
7.203
6.655
6.115
6.042
5.720
5.393
5.352
4.960
4.948
4.481
4.163
4.114
3.746
3.733
3.167
3.143
2.798
2.617
2.185
2.168
1.843
1.255
0.993
0.862
-0.001
-0.003
-0.037
-0.150

LC-MS chromatograms of compounds

SN38-(PEG)$_1$-Pom-α

Sample Information

Acquired by	: System Administrator
Date Acquired	: 2021/07/09 14:02:41
Sample Name	: PROTAC-PEG1
Tray#	: 2
Vial#	: 1
Injection Volume	: 10
Data File	: PROTAC_20210709_20210709_002.lcd
Method File	: default_posi_nega.lcm
Tuning File	: 20190726.lct
Date Processed	: 2021/07/09 14:32:42

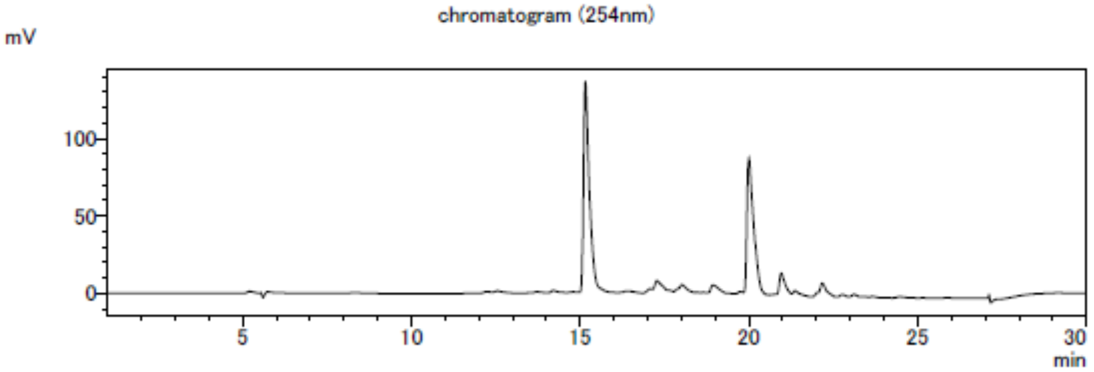

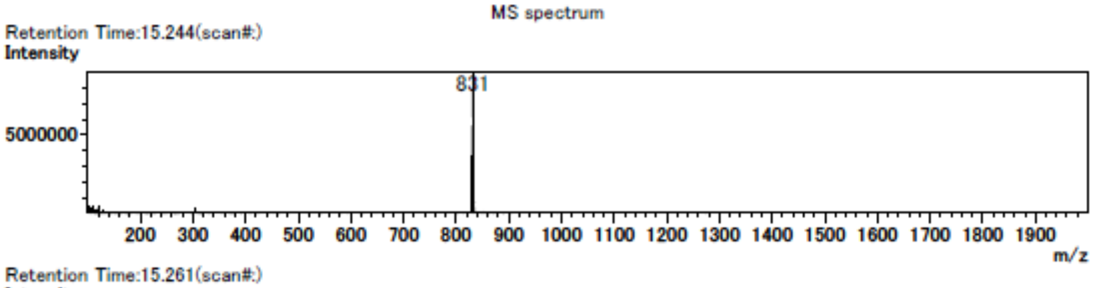

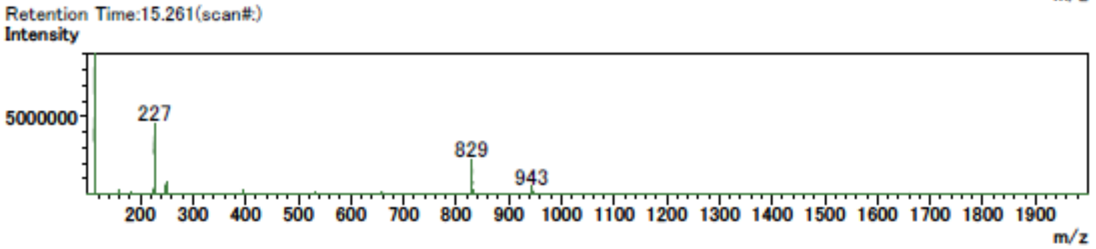

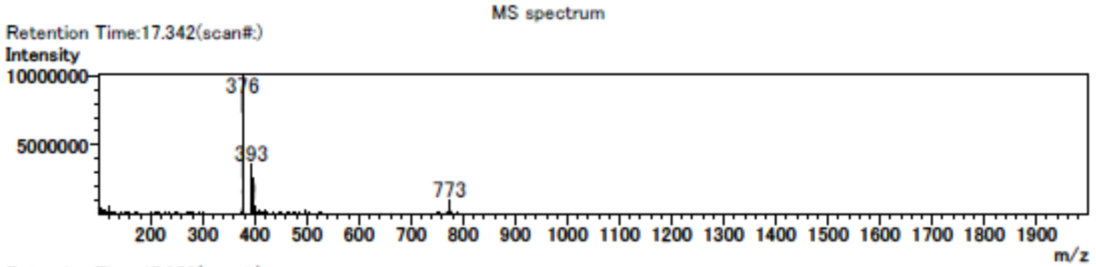

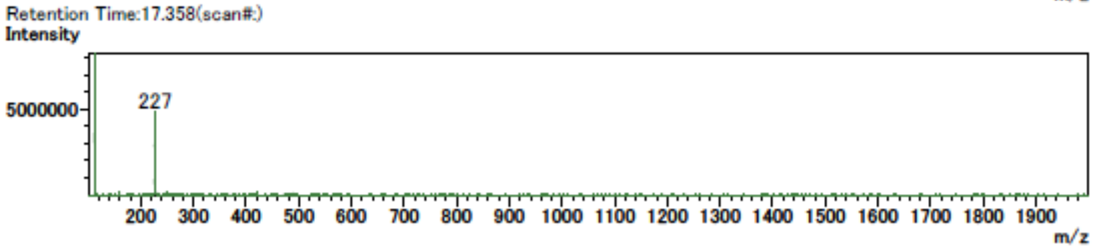

SN38-(PEG)$_2$-Pom-α

Sample Information

Acquired by	: System Administrator
Date Acquired	: 2021/07/09 14:33:05
Sample Name	: PROTAC-PEG2
Tray#	: 2
Vial#	: 2
Injection Volume	: 10
Data File	: PROTAC_20210709_20210709_003.lcd
Method File	: default_posi_nega.lcm
Tuning File	: 20190726.lct
Date Processed	: 2021/07/09 15:03:07

chromatogram (254nm)

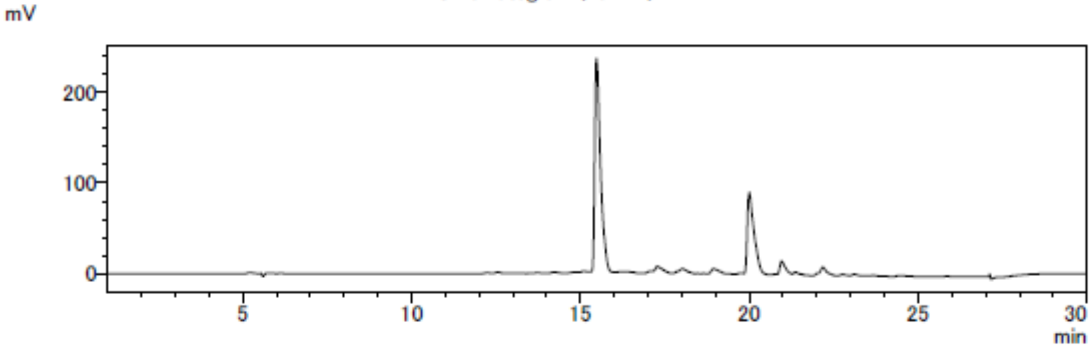

MS spectrum

Retention Time:15.515(scan#:)

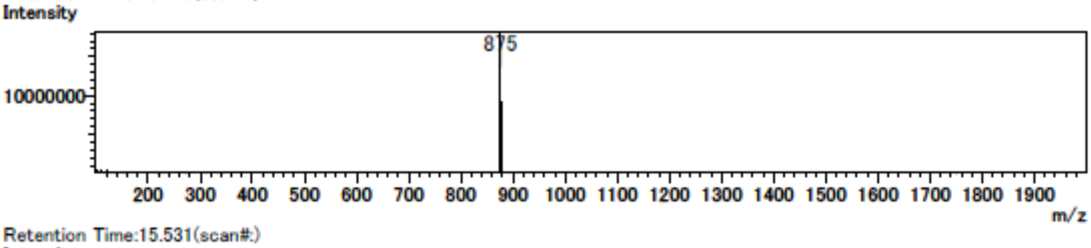

Retention Time:15.531(scan#:)

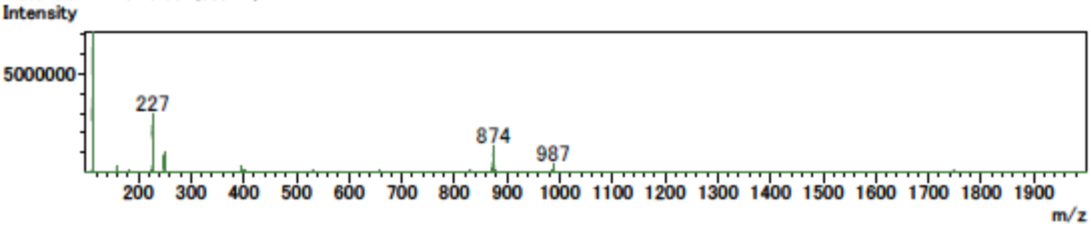

MS spectrum

Retention Time:17.375(scan#:)

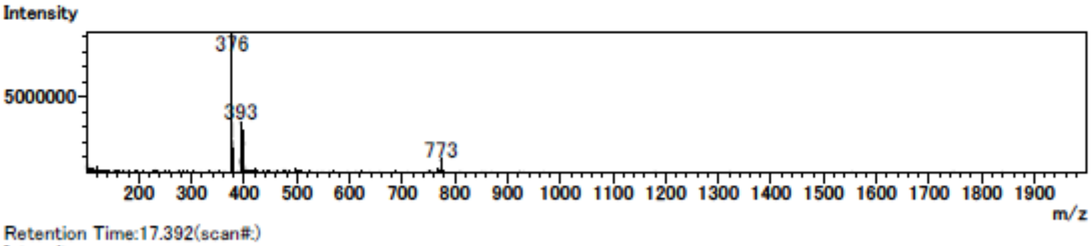

Retention Time:17.392(scan#:)

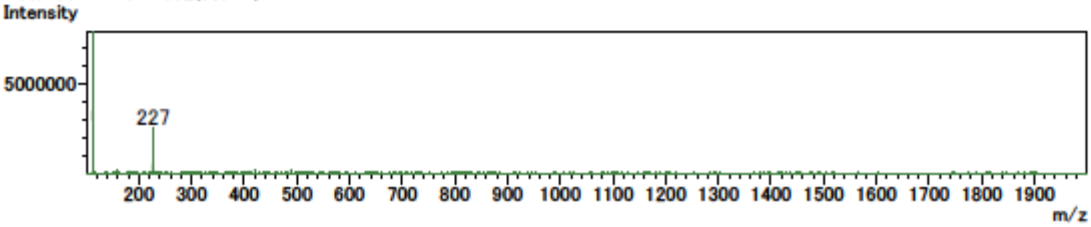

SN38-(PEG)$_3$-Pom-α

Sample Information

Acquired by	: System Administrator
Date Acquired	: 2021/07/09 15:03:31
Sample Name	: PROTAC-PEG3
Tray#	: 2
Vial#	: 3
Injection Volume	: 10
Data File	: PROTAC_20210709_20210709_004.lcd
Method File	: default_posi_nega.lcm
Tuning File	: 20190726.lct
Date Processed	: 2021/07/09 15:33:34

chromatogram (254nm)

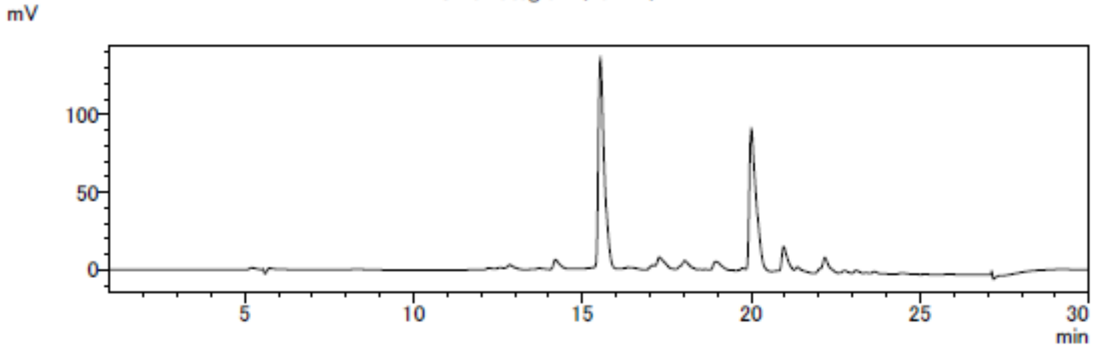

MS spectrum

Retention Time:15.616(scan#:)

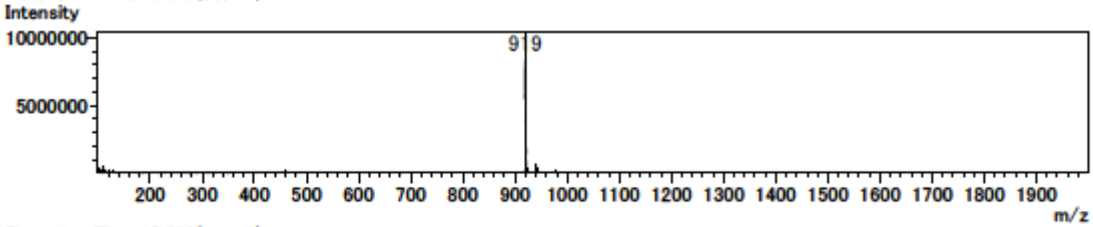

Retention Time:15.633(scan#:)

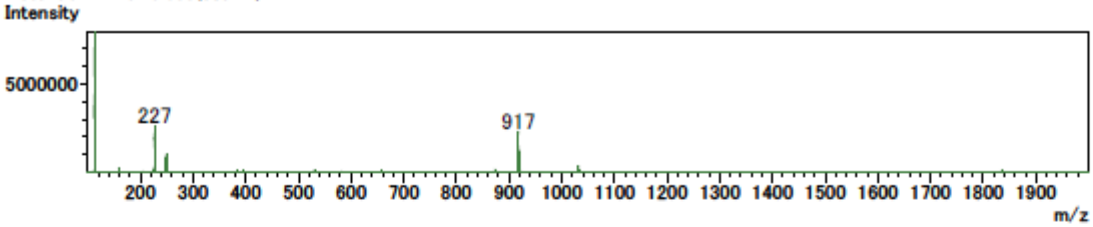

MS spectrum

Retention Time:17.342(scan#:)

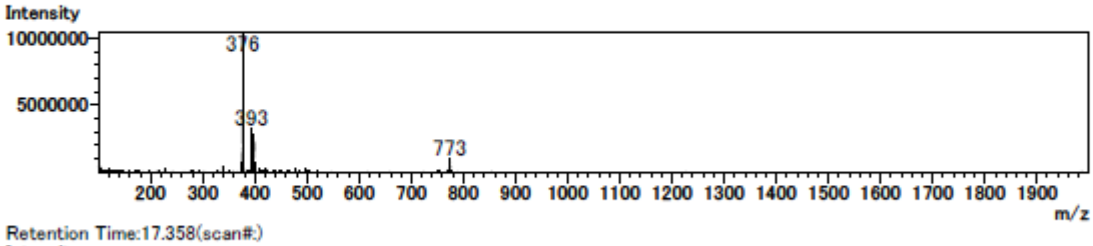

Retention Time:17.358(scan#:)

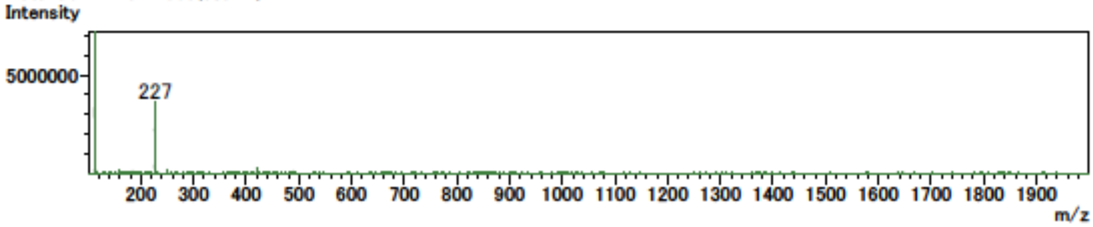

SN38-(PEG)$_4$-Pom-α

Sample Information

Acquired by	: System Administrator
Date Acquired	: 2020/07/01 8:56:28
Sample Name	: prptac_20200701_PEG4
Tray#	: 2
Vial#	: 1
Injection Volume	: 5
Data File	: prptac_20200701_PEG4_20200701_001.lcd
Method File	: default_posi_nega.lcm
Tuning File	: 20190726.lct
Date Processed	: 2020/07/01 9:26:29

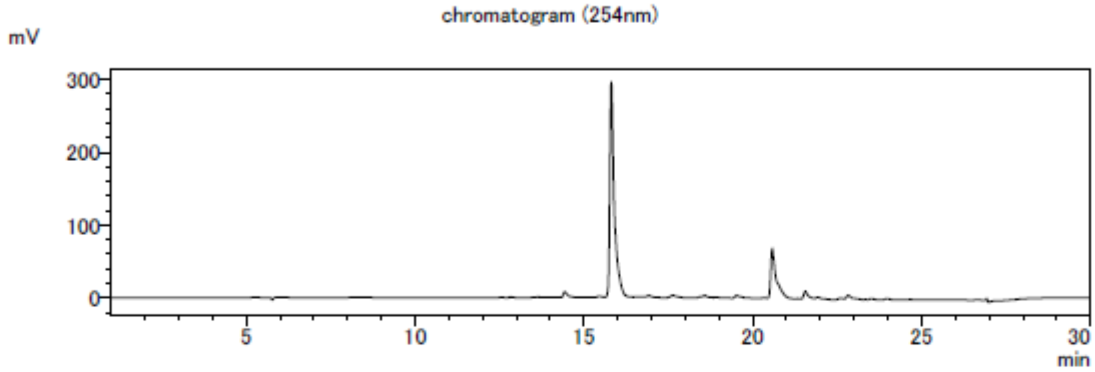

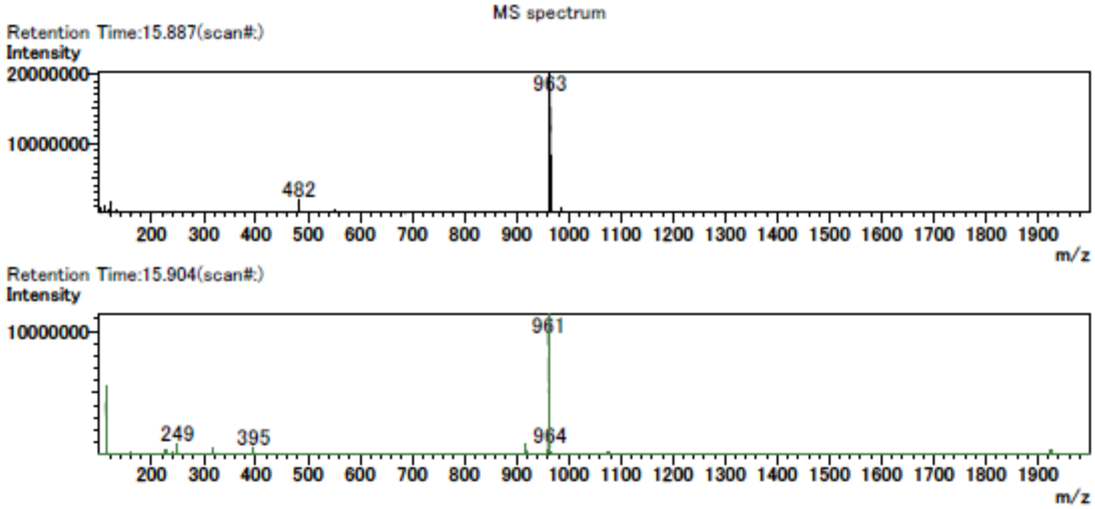

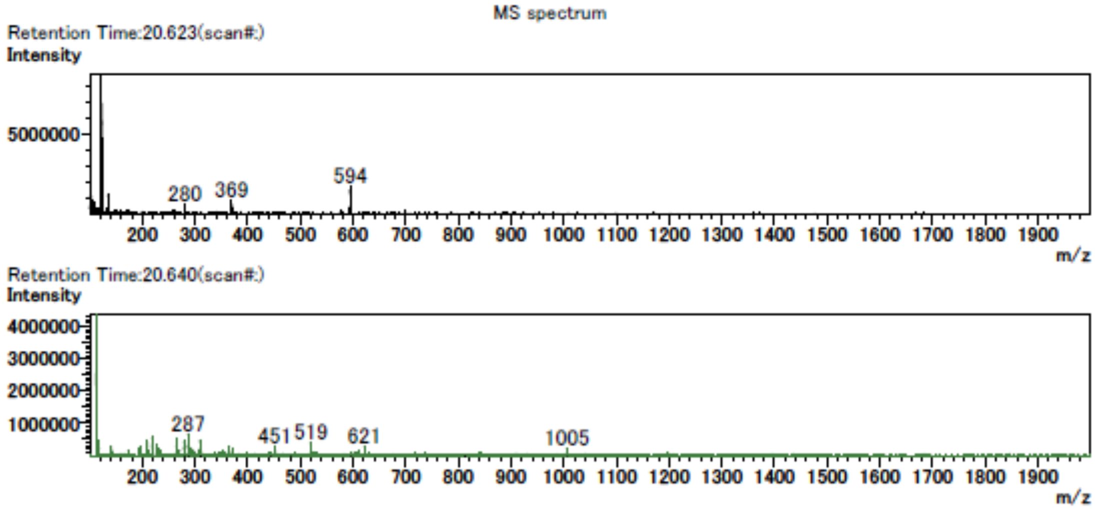

SN38-(PEG)$_5$-Pom-α

Sample Information

Acquired by	: System Administrator
Date Acquired	: 2020/07/01 9:26:52
Sample Name	: prptac_20200701_PEG5
Tray#	: 2
Vial#	: 2
Injection Volume	: 5
Data File	: prptac_20200701_PEG4_20200701_002.lcd
Method File	: default_posi_nega.lcm
Tuning File	: 20190726.lct
Date Processed	: 2020/07/01 9:56:54

chromatogram (254nm)

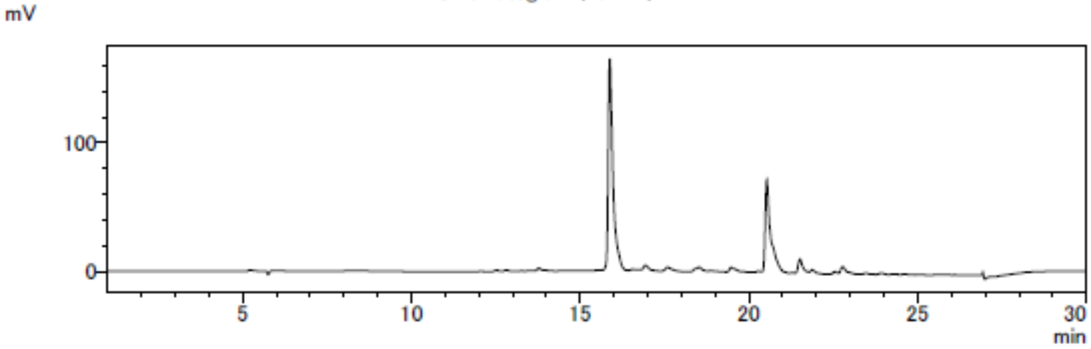

MS spectrum

Retention Time:15.954(scan#:)

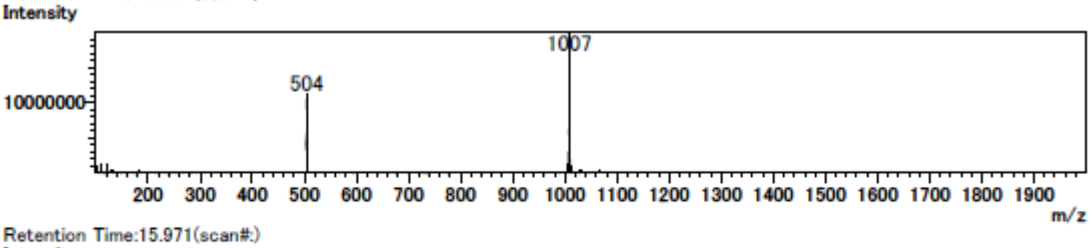

Retention Time:15.971(scan#:)

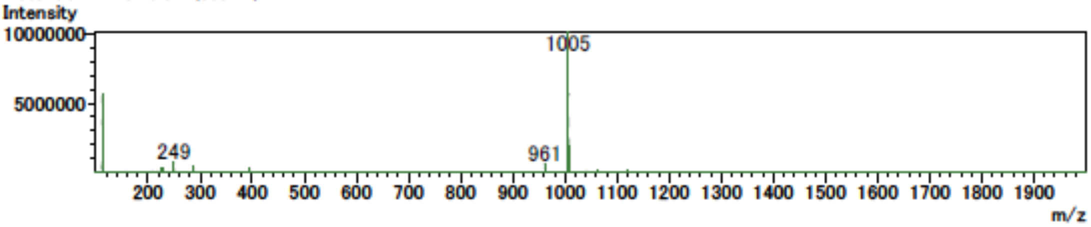

MS spectrum

Retention Time:20.623(scan#:)

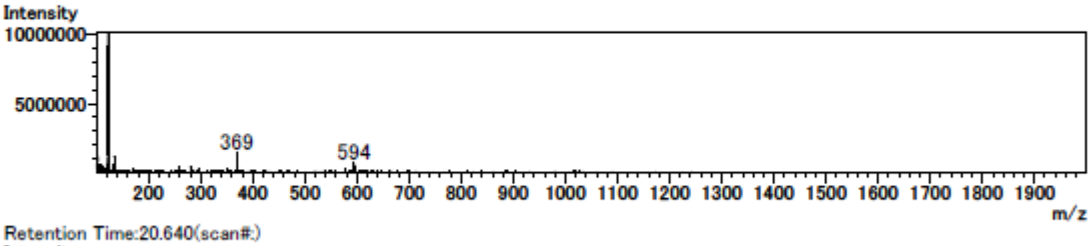

Retention Time:20.640(scan#:)

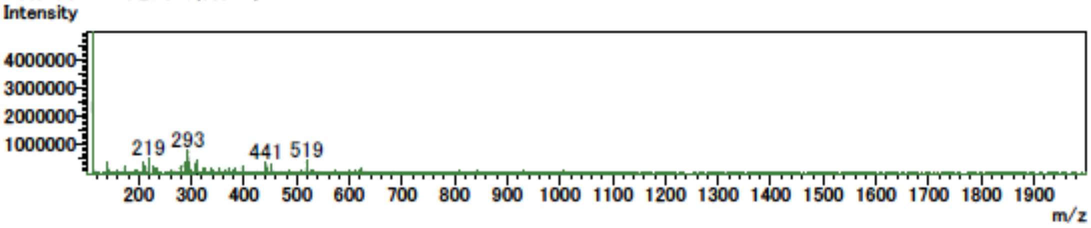

SN38-(PEG)$_6$-Pom-α

Sample Information

Acquired by	: System Administrator
Date Acquired	: 2020/07/01 9:57:16
Sample Name	: prptac_20200701_PEG6
Tray#	: 2
Vial#	: 3
Injection Volume	: 5
Data File	: prptac_20200701_PEG4_20200701_003.lcd
Method File	: default_posi_nega.lcm
Tuning File	: 20190726.lct
Date Processed	: 2020/07/01 10:27:17

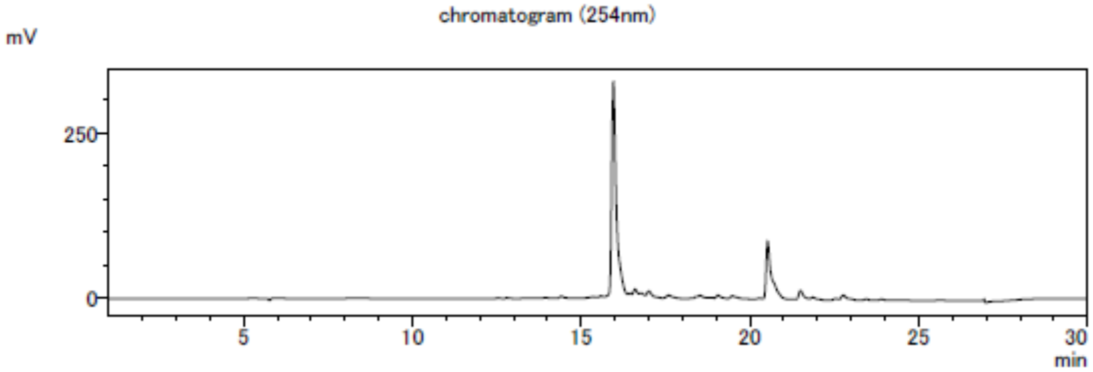

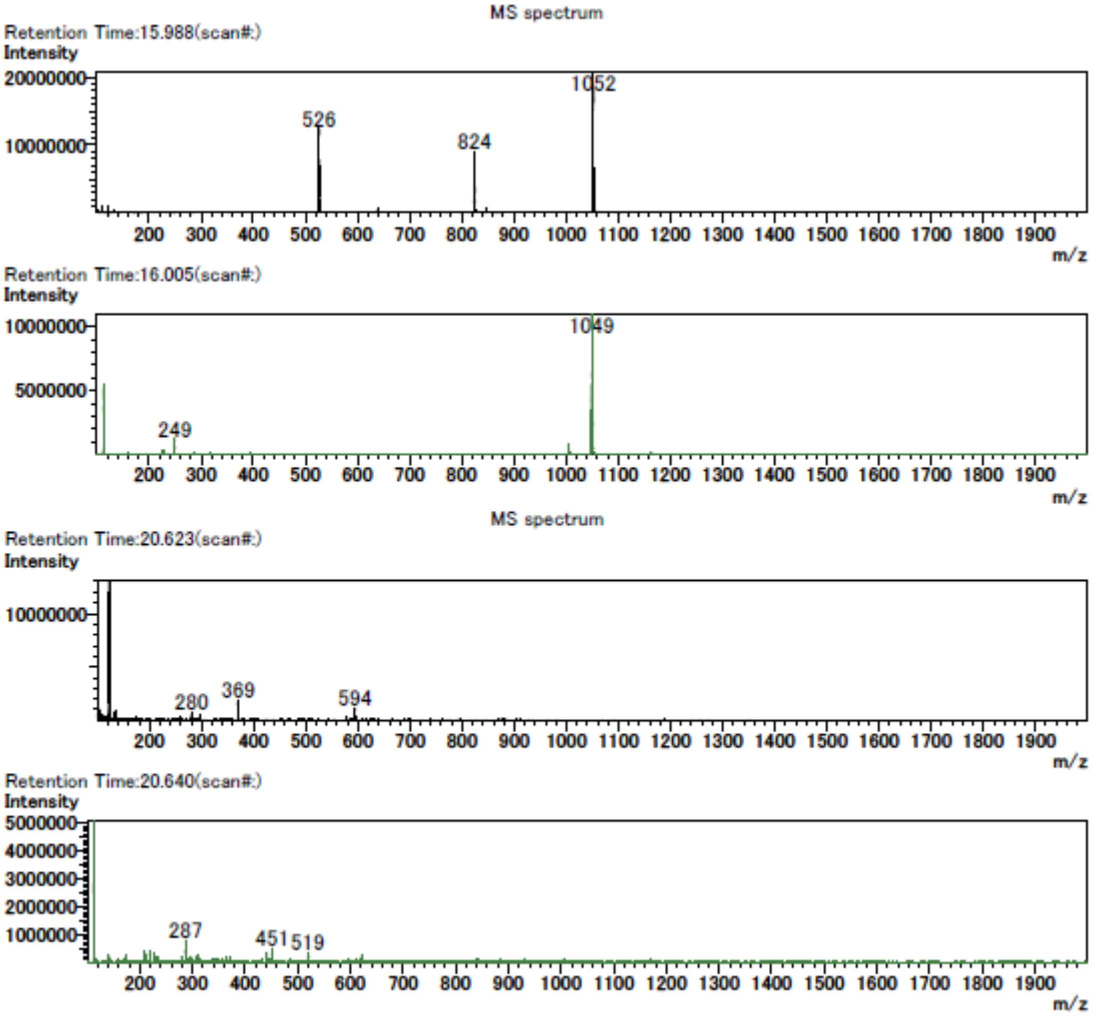

SN38-(PEG)$_1$-Pom-β

Sample Information

Acquired by : System Administrator
Date Acquired : 2021/05/19 10:27:17
Sample Name : 5C-PEG1_50ug/ml
Tray# : 1
Vial# : 1
Injection Volume : 5
Data File : 5C-PEG_20210519_20210519_001.lcd
Method File : default_posi_nega.lcm
Tuning File : 20190726.lct
Date Processed : 2021/05/19 10:57:19

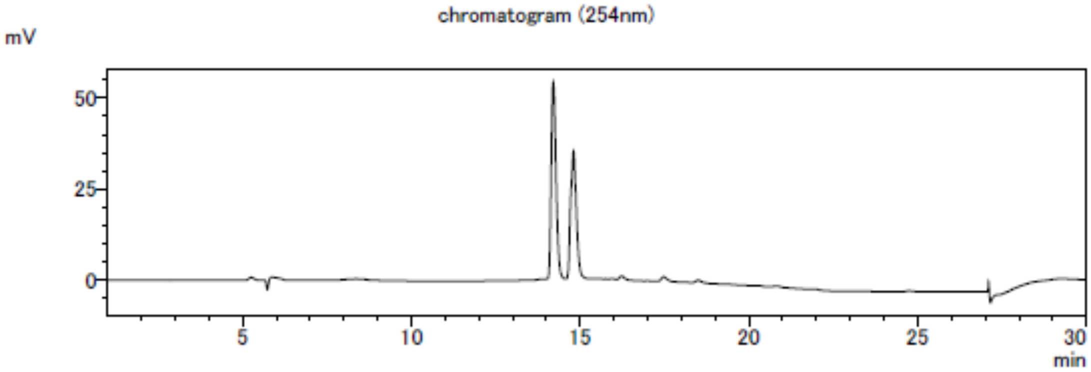

MS spectrum

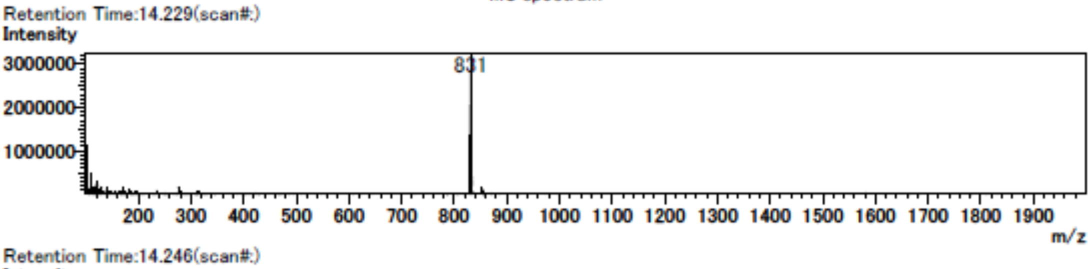

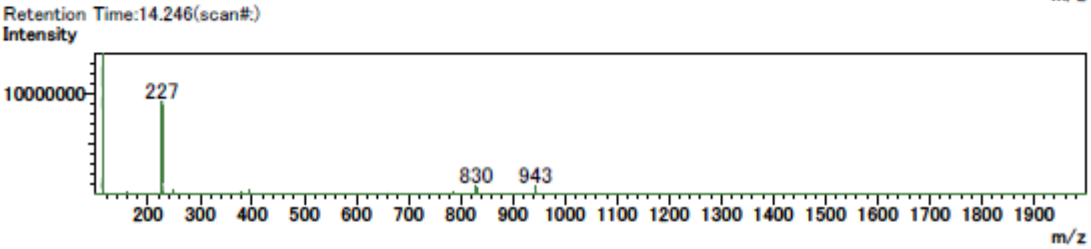

MS spectrum

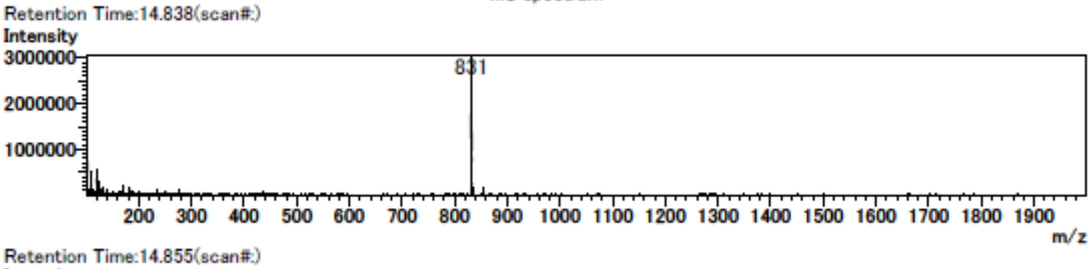

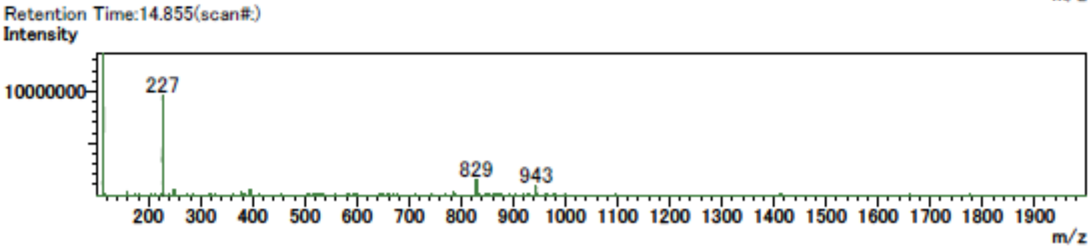

SN38-(PEG)$_2$-Pom-β

Sample Information

Acquired by : System Administrator
Date Acquired : 2021/05/19 10:57:40
Sample Name : 5C-PEG2_50ug/ml
Tray# : 1
Vial# : 2
Injection Volume : 5
Data File : 5C-PEG_20210519_20210519_002.lcd
Method File : default_posi_nega.lcm
Tuning File : 20190726.lct
Date Processed : 2021/05/19 11:27:43

chromatogram (254nm)

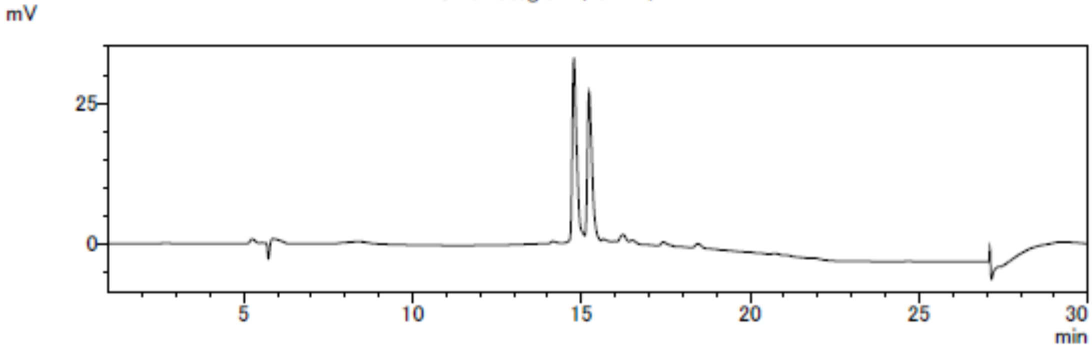

MS spectrum

Retention Time:14.838(scan#:)
Intensity

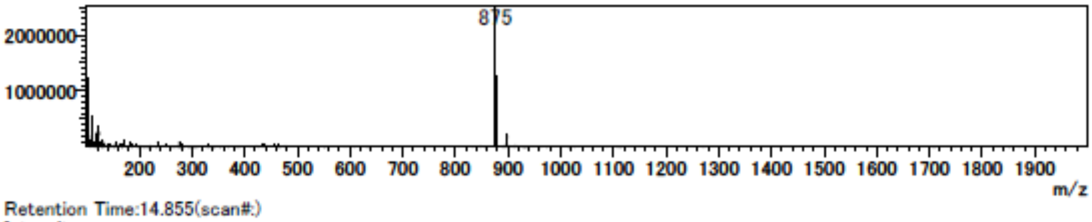

Retention Time:14.855(scan#:)
Intensity

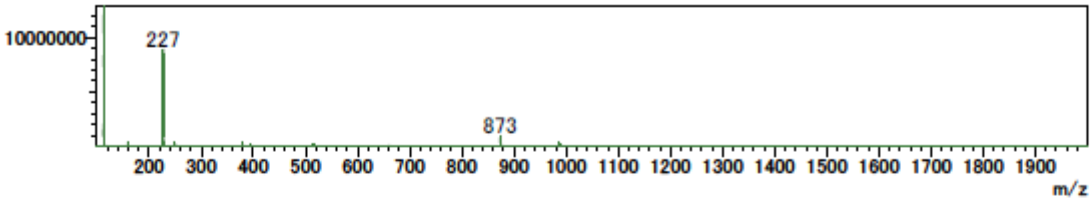

MS spectrum

Retention Time:15.278(scan#:)
Intensity

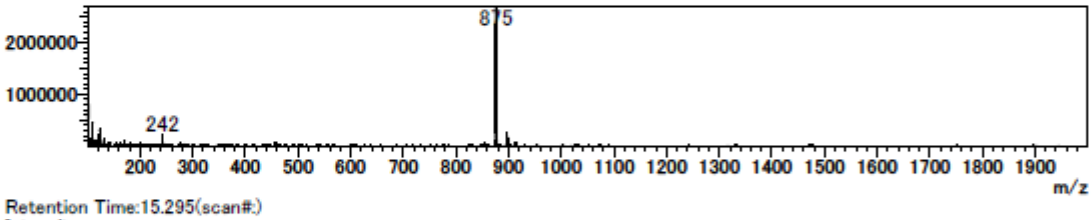

Retention Time:15.295(scan#:)
Intensity

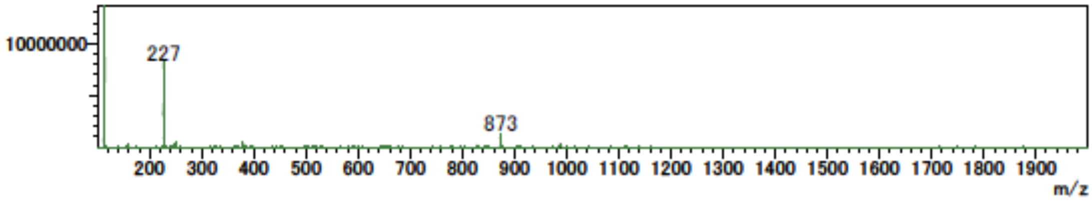

SN38-(PEG)$_3$-Pom-β

Sample Information

Acquired by	: System Administrator
Date Acquired	: 2021/05/19 11:28:05
Sample Name	: 5C-PEG3_50ug/ml
Tray#	: 1
Vial#	: 3
Injection Volume	: 5
Data File	: 5C-PEG_20210519_20210519_003.lcd
Method File	: default_posi_nega.lcm
Tuning File	: 20190726.lct
Date Processed	: 2021/05/19 11:58:06

chromatogram (254nm)

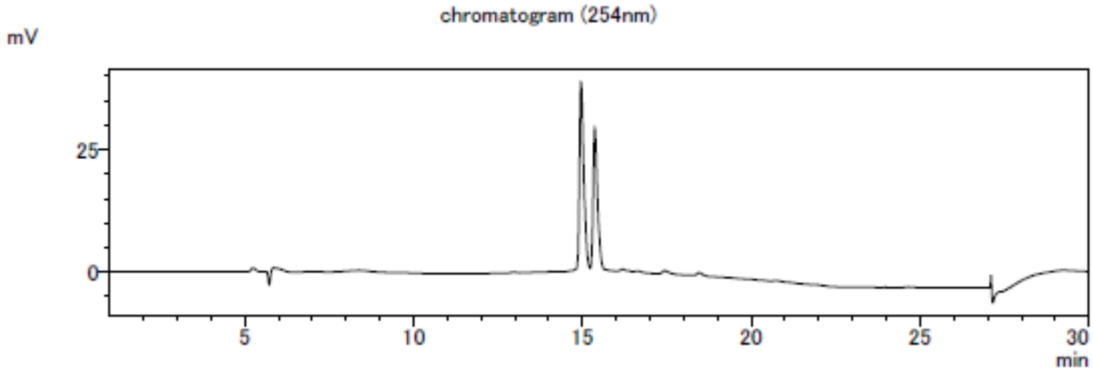

MS spectrum

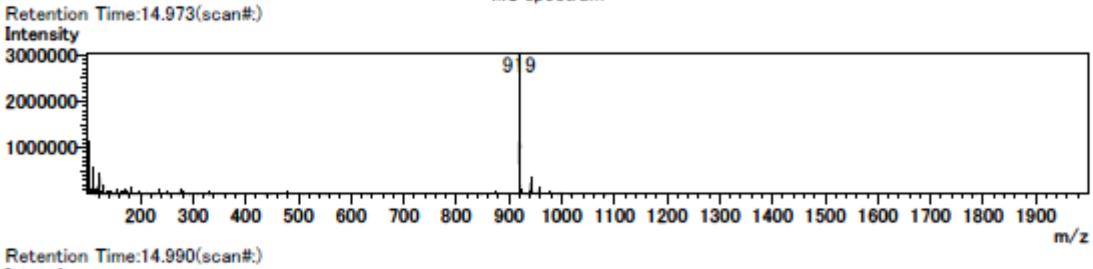

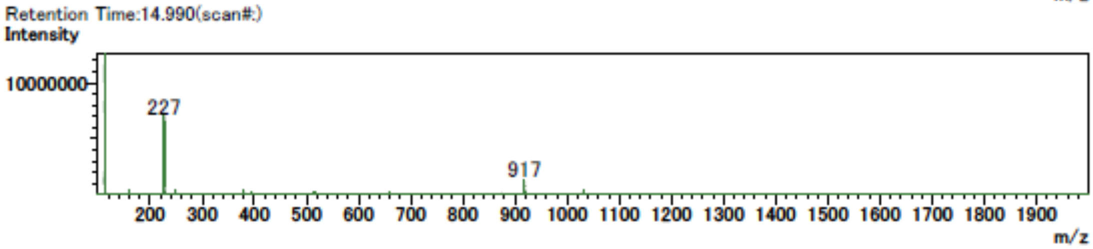

MS spectrum

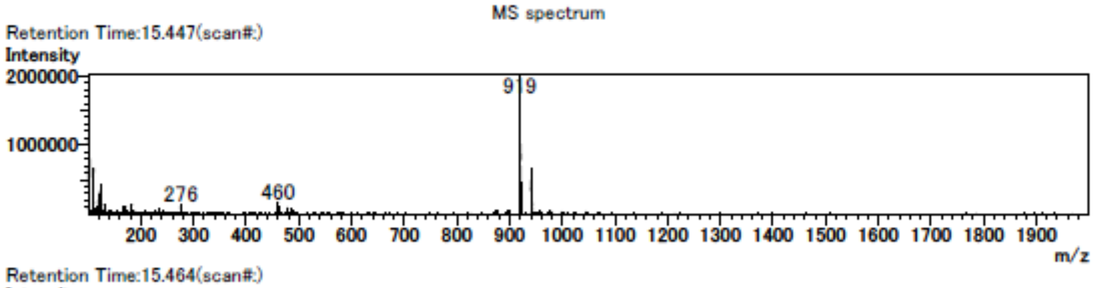

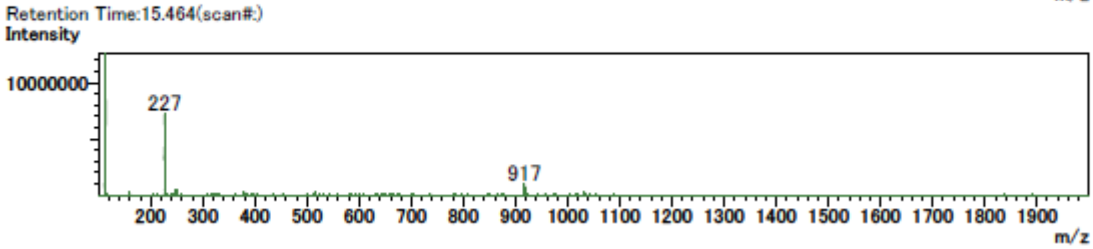

SN38-(PEG)$_4$-Pom-β

Sample Information

Acquired by	: System Administrator
Date Acquired	: 2021/05/19 11:58:28
Sample Name	: 5C-PEG4_50ug/ml
Tray#	: 1
Vial#	: 4
Injection Volume	: 5
Data File	: 5C-PEG_20210519_20210519_004.lcd
Method File	: default_posi_nega.lcm
Tuning File	: 20190726.lct
Date Processed	: 2021/05/19 12:28:30

chromatogram (254nm)

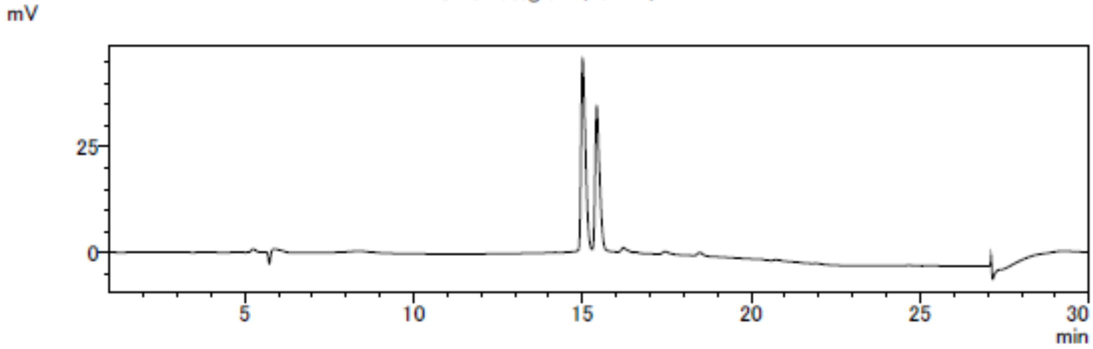

MS spectrum

Retention Time:15.075(scan#:)

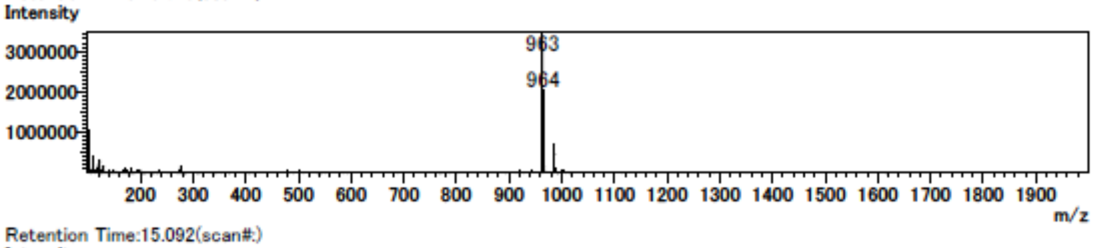

Retention Time:15.092(scan#:)

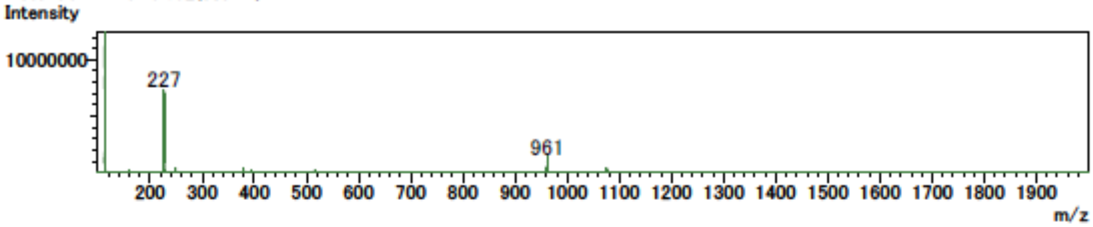

MS spectrum

Retention Time:15.447(scan#:)

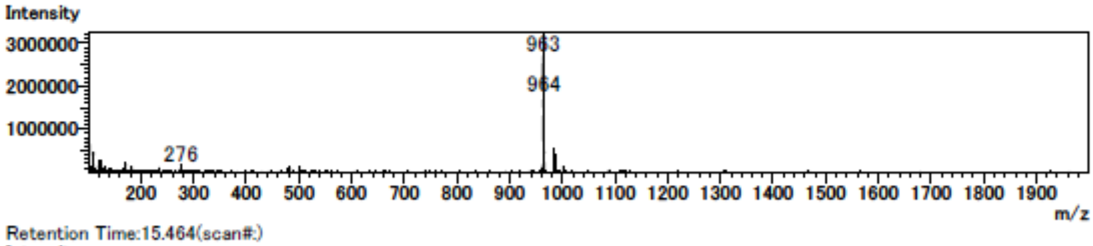

Retention Time:15.464(scan#:)

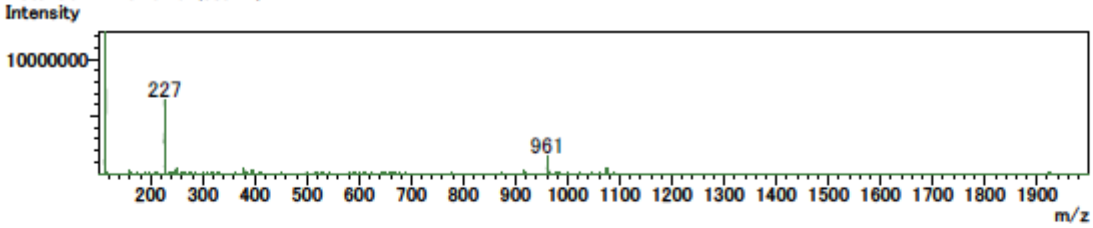

SN38-(PEG)$_5$-Pom-β

Sample Information

Acquired by	: System Administrator
Date Acquired	: 2021/05/19 12:28:52
Sample Name	: 5C-PEG5_50ug/ml
Tray#	: 1
Vial#	: 5
Injection Volume	: 5
Data File	: 5C-PEG_20210519_20210519_005.lcd
Method File	: default_posi_nega.lcm
Tuning File	: 20190726.lct
Date Processed	: 2021/05/19 12:58:53

chromatogram (254nm)

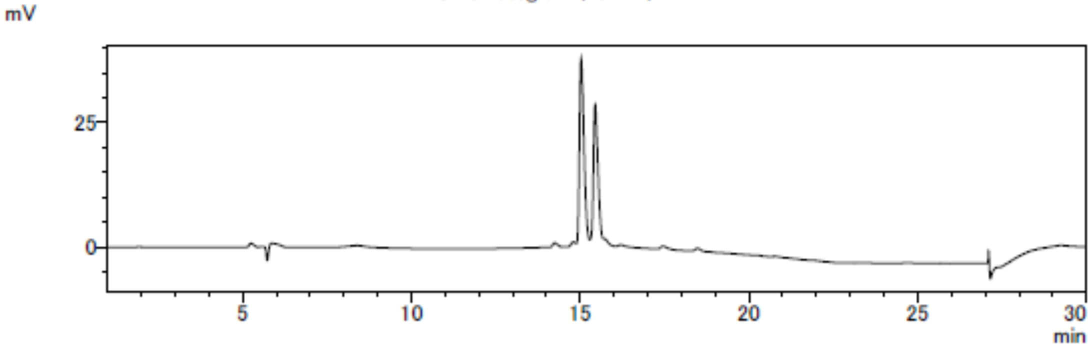

MS spectrum

Retention Time:15.075(scan#:)

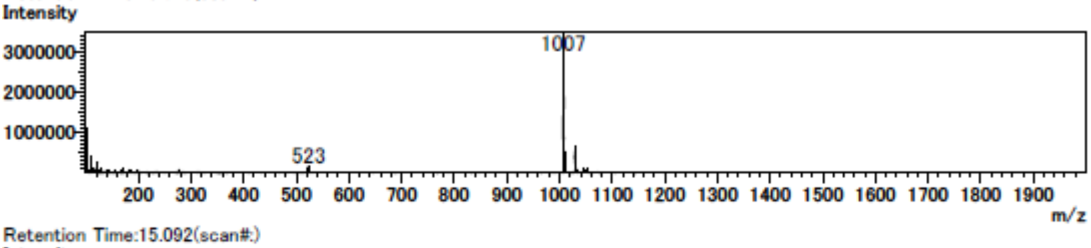

Retention Time:15.092(scan#:)

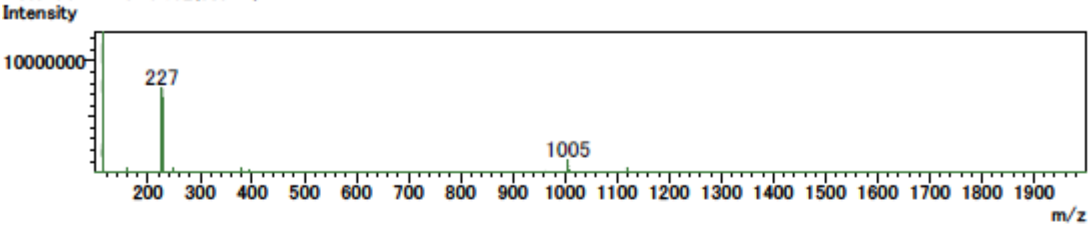

MS spectrum

Retention Time:15.515(scan#:)

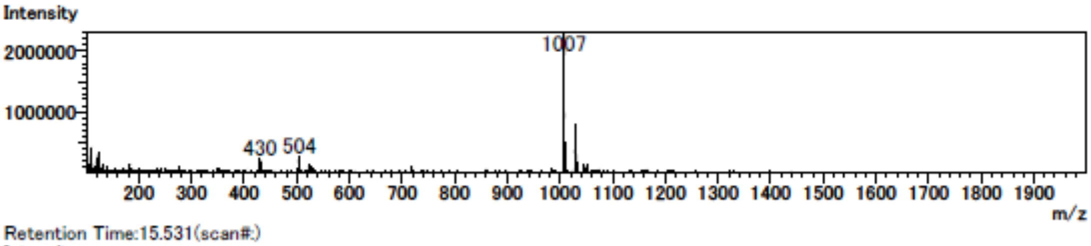

Retention Time:15.531(scan#:)

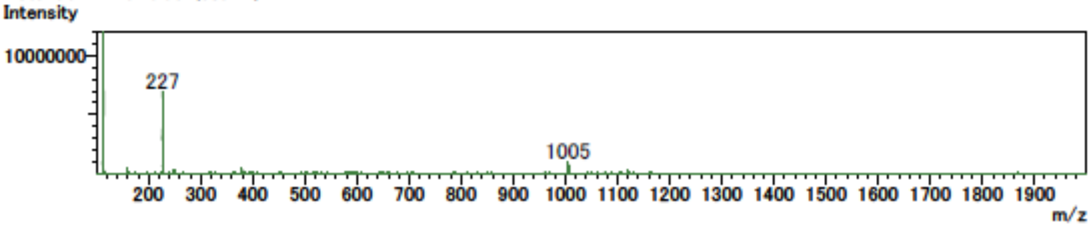

SN38-(PEG)$_6$-Pom-β

Sample Information

Acquired by	: System Administrator
Date Acquired	: 2021/05/19 12:59:15
Sample Name	: 5C-PEG6_50ug/ml
Tray#	: 1
Vial#	: 6
Injection Volume	: 5
Data File	: 5C-PEG_20210519_20210519_006.lcd
Method File	: default_posi_nega.lcm
Tuning File	: 20190726.lct
Date Processed	: 2021/05/19 13:29:17

chromatogram (254nm)

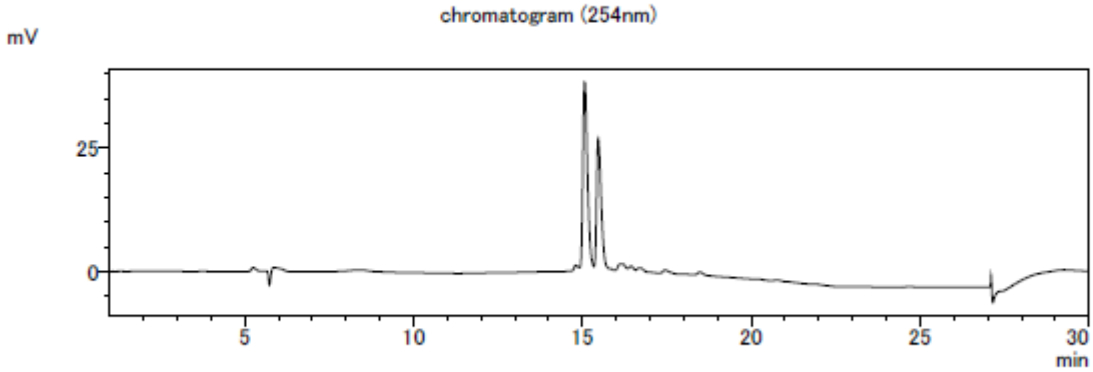

MS spectrum

Retention Time:15.109(scan#:)

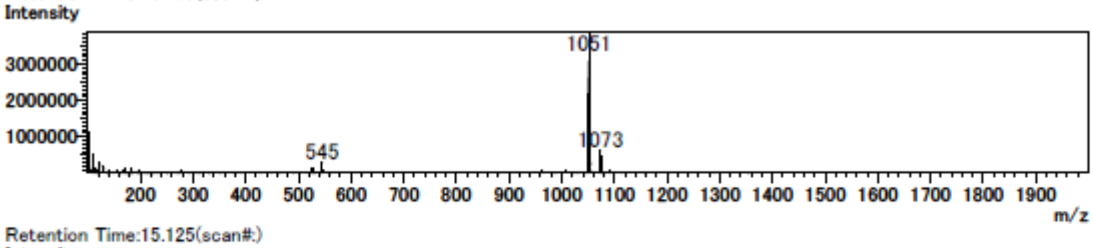

Retention Time:15.125(scan#:)

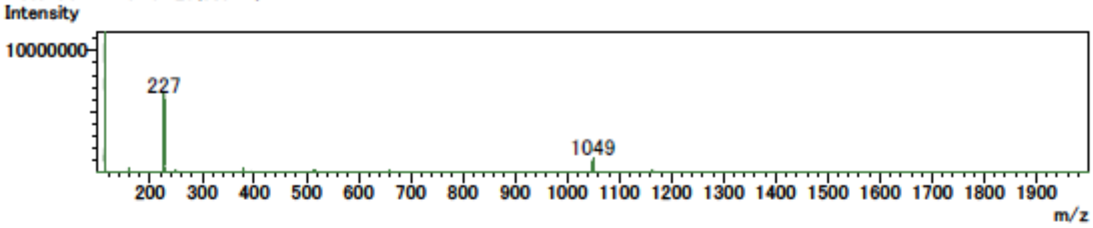

MS spectrum

Retention Time:15.548(scan#:)

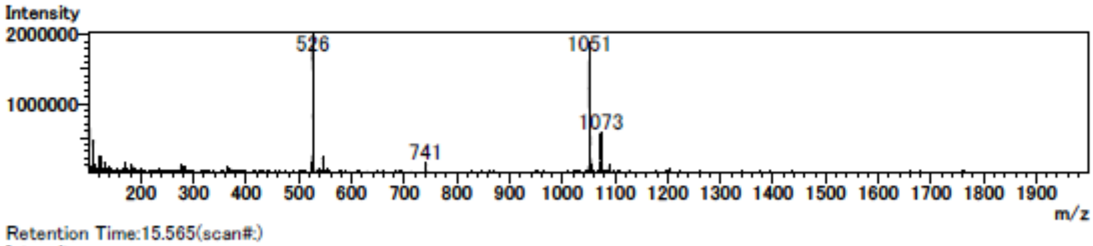

Retention Time:15.565(scan#:)

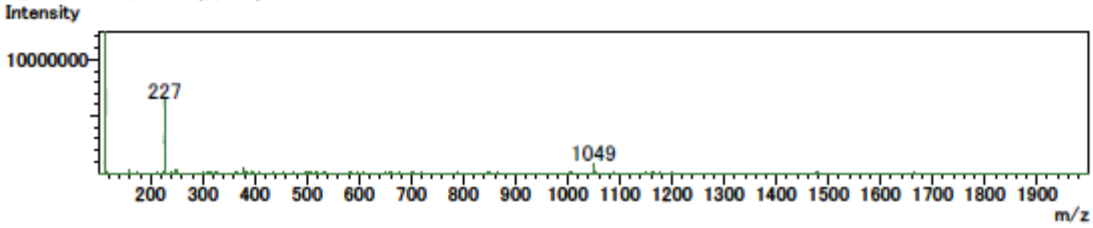

Supplement: Supplementary file 2 — Supplementary methods [file 41388_2025_3641_MOESM2_ESM.pdf]
